# AI for Detecting and Predicting Postpartum Depression: Scoping Review

**DOI:** 10.2196/77376

**Published:** 2026-01-08

**Authors:** Mais Alkhateeb, Ajisha Nayeem, Arfan Ahmed, Mohammed Alsahli, Javaid Sheikh, Alaa Abd-Alrazaq

**Affiliations:** 1 College of Education and Art, Lusail University, Doha, Qatar, +974440119502; 2 AI Center for Precision Health, Weill Cornell Medicine-Qatar, Doha, Qatar; 3 Computer Science and Engineering, Hamad bin Khalifa University, Qatar Foundation, Doha, Qatar; 4 Health Informatics Department, College of Health Science, Saudi Electronic University, Riyadh, Saudi Arabia

**Keywords:** postpartum depression, maternal mental health, perinatal depression, artificial intelligence, machine learning, deep learning, natural language processing, prediction models, computer-aided diagnosis

## Abstract

**Background:**

Postpartum depression (PPD) affects up to 20% of mothers globally. Early detection is vital for better outcomes, yet screening lacks scalability and predictive power. Artificial intelligence (AI)—through machine learning, deep learning, and natural language processing—enhances the early identification of mothers at risk with greater accuracy.

**Objective:**

This study aims to systematically map the existing literature on AI-based methods for detecting and predicting PPD.

**Methods:**

This scoping review was conducted in accordance with the PRISMA-ScR (Preferred Reporting Items for Systematic Reviews and Meta-Analyses Extension for Scoping Reviews) guidelines. We included empirical studies that applied AI techniques to detect or predict PPD and were published in peer-reviewed journals, conference proceedings, or dissertations. Studies were excluded if they were nonempirical (eg, reviews, editorials, and abstracts), not published in English, focused on general perinatal mental health without a specific emphasis on PPD, or used AI solely for monitoring or treatment rather than prediction or detection. We systematically searched 8 databases—MEDLINE, Embase, PsycINFO, CINAHL, Scopus, IEEE Xplore, ACM Digital Library, and Google Scholar—from inception through February 28, 2025. The search strategy was supplemented by backward and forward reference screening and biweekly alerts to capture newly published studies. Two independent (M [Alkhateeb] and A [Nayeem])reviewers (M [Alkhateeb] and A [Nayeem]) screened the retrieved studies, with disagreements resolved by a third reviewer (AA [Alrazaq]). Data were extracted by 2 independent reviewers using a standardized extraction form capturing study characteristics, AI model types, data sources, features, preprocessing, validation strategies, and performance metrics. A formal risk-of-bias assessment was not performed due to the scoping nature of the review. All extracted data were synthesized narratively.

**Results:**

Out of 503 retrieved studies, 65 met the inclusion criteria. The United States contributed the largest proportion of studies (18/65, 27.7%). The highest number of publications occurred in 2024 (17/65, 26%). Most included studies were journal articles (46/65, 71%). Short-term postpartum outcomes (≤12 weeks) were most frequently assessed (20/65, 30.8%). Most included studies (52/65, 80%) applied AI models for predicting PPD, while 14 of 65 (22%) studies used them for detection. Sociodemographic data were most frequently used (49/65, 75.4%), followed by psychological data (44/65, 68%) and obstetric data (35/65, 55%). Data preprocessing mostly relied on basic scaling (51/65, 79%) and some missing data imputation (29/65, 44.6%). Machine learning dominated (57/65, 87.7%), especially random forest, support vector machines, and logistic regression. Internal validation (k-fold, hold-out) was standard, while external validation was scarce. Ensemble-based boosting models consistently demonstrated superior performance across key metrics, highlighting their potential for accurate and scalable PPD prediction. Current studies suffer from limited sample sizes, geographic bias, lack of standardized feature sets, minimal external validation, and inconsistent reporting of comprehensive model metrics.

**Conclusions:**

This scoping review analyzes 65 studies on AI in PPD, highlighting dominant use of classical machine learning, limited deep learning adoption, underuse of advanced preprocessing, inconsistent validation, and reliance on structured, unimodal data—mainly sociodemographic, clinical, and obstetric features.

## Introduction

### Background

Postpartum depression (PPD) is a common mental health issue affecting new mothers after they give birth. Its salient features include feelings of enduring melancholy, losing interest in hobbies and their everyday life (and, potentially, their baby), and reduced feelings of pleasure in activities.

Traditionally referred to as the “baby blues,” PPD is a profound and serious condition undermining the activities of daily life and the psychosocial well-being of mothers. It affects up to a fifth of new mothers worldwide, albeit it is often undiagnosed; in any case, it is a major concern for public health [1].

Postpartum care is vital to ensure the best outcomes for both neonates and mothers. This includes creating a supportive environment with health-promoting activities and breastfeeding encouragement. It must also address each mother’s individual mental health needs. [2].

Worldwide, studies of perinatal mental health have noted that PPD is increasingly evident [3]. According to the estimations of the National Institute of Mental Health, up to 15% of all women who experience pregnancy also experience related depression, whether during or after pregnancy. Prevalence is typically higher (ie, 18%‐25%) in low- and middle-income countries, where it is associated with socioeconomic issues and health care resource availability and access, as well as sociocultural factors [4].

The great variety in PPD prevalence underscores the requirement for efficacious strategies for screening women and delivering interventions catering to various needs. The mainstay for PPD detection now is dependent on women reporting classic symptoms and completing self-reported tools, of which the Edinburgh Postnatal Depression Scale (EPDS) [5] and the Patient Health Questionnaire-9 (PHQ-9) [6] are common and effective. However, such tools for screening women during and after pregnancy are typically not administered consistently. For instance, women are often screened once during the early stages of pregnancy, such as the second trimester. However, the same tools are rarely reapplied in later or postpartum periods [7,8].

Furthermore, the tools detect current depression, with no scope to anticipate future risk (based on current symptoms and feelings) [9]. Prediction and early diagnosis of PPD remain challenging, largely because qualitative narrative data are difficult to interpret and integrate alongside quantitative clinical metrics.

A detailed professional analysis is necessary to interpret data appropriately, which is costly, time-consuming, and potentially subjective [10]. PPD prevention and treatment interventions require improved screening solutions that can be delivered during early pregnancy and throughout the pregnancy journey and postpartum period.

Artificial intelligence (AI) can potentially address this impasse, with its capability to handle and process vast volumes of complex, high-dimensional, nonlinear data. Using machine learning (ML), large language models, and natural language processing (NLP) techniques, AI can detect subtle patterns inherent within data that could otherwise evade human analysis [10,11].

AI can enhance prediction accuracy by incorporating diverse data sources. These include electronic health records (EHRs), diagnostic indicators, self-reported feelings, and behavioral cues gathered from digital platforms, with appropriate safeguards [1,12]. Such possibilities render AI a highly useful clinical tool, offering real-time decision-making input for digital care delivery.

### Research Problem and Aim

Many studies have developed AI models for detecting and predicting PPD, yet these studies offer fragmented insights into the full potential of AI methodologies. Several previous reviews attempted to summarize these insights [10,13-19], but they have notable limitations. Specifically, some prior reviews were traditional narrative reviews rather than systematic or scoping reviews and thus lacked rigorous, structured methodologies [13,14,16].

In addition, many earlier reviews used narrow search queries or omitted critical databases (eg, PsycINFO, ACM Digital Library, IEEE Xplore, Scopus, and Embase), potentially excluding relevant studies [10,13-19]. Furthermore, past reviews often broadly addressed general depression or women’s mental health instead of specifically targeting PPD, limiting their direct relevance [10,15]. Also, the bibliographic searches of previous reviews mostly concluded before September 2022, omitting recent advancements in AI methodologies and multimodal data integration techniques. Importantly, most prior research emphasized traditional clinical and survey-based data, neglecting innovative data sources such as social media and wearable sensors. These novel data sources represent a promising opportunity to enhance AI model accuracy and predictive capabilities for PPD [10,13,14,16-19].

The primary aim of this review is to map the landscape of AI methodologies used in PPD detection and prediction and to identify key research trends, methodological features, and evidence gaps. Specifically, this review is guided by the following research subquestions:

- What types of AI models have been used to detect or predict PPD, and how do they differ in approach and complexity?
- What data modalities (eg, structured, unstructured, physiological, and digital) have been used in these studies?
- How have studies handled model development processes such as feature selection, validation, and interpretability?
- What are the key limitations, challenges, and future opportunities for applying AI to PPD detection in real-world clinical and community settings?

By addressing these questions, this review provides a structured, up-to-date, and integrative overview of AI in postpartum mental health—highlighting opportunities for innovation, responsible deployment, and policy translation in maternal care.

## Methods

We conducted a scoping review in accordance with the PRISMA-ScR (Preferred Reporting Items for Systematic Reviews and Meta-Analyses Extension for Scoping Reviews) guidelines (see PRISMA-ScR checklist). The following sections detail the specific methods we used in this review.

### Search Strategy

All-inclusive searches were done across the following 8 major electronic databases on November 18, 2024, to determine relevant studies: MEDLINE (via Ovid), PsycINFO (via Ovid), Embase (via Ovid), CINAHL (via EBSCO), IEEE Xplore, ACM Digital Library, Scopus, and Google Scholar. To keep our search up to date, we set up an automatic biweekly search alert for 24 weeks, ending on February 28, 2025. Given the vast number of results generated by Google Scholar, we focused on the first 100 results (10 pages), as they are ranked by relevance. In addition to database searches, we expanded our review by manually screening reference lists of included studies (backward reference checking) and identifying studies that cited them (forward reference checking). We also collected additional papers through automatic email alerts. To ensure that the search query was well-structured and effective, 3 experts in digital mental health were consulted and previous relevant literature was reviewed. Two main categories of terms were included in the final search query: AI-related terms (eg, artificial intelligence, machine learning, and deep learning) and PPD-related terms (eg, postpartum depression, postpartum depression, and postnatal depression). A detailed search query used for each database is shown in Multimedia Appendix 1.

### Study Eligibility Criteria

This scoping review targeted studies that specifically applied AI to the detection or prediction of PPD. Eligible studies were empirical in nature, used AI methodologies, and were published in peer-reviewed journals, dissertations, or conference proceedings. There were no restrictions regarding publication year, country of origin, data type, study design, population, or outcome type.

Exclusion criteria encompassed nonempirical works such as reviews, abstracts, commentaries, and proposals, as well as studies lacking a specific focus on PPD (eg, addressing broader maternal or perinatal mental health). Studies that used AI solely for managing or monitoring PPD, rather than detecting or predicting it, were also excluded. In addition, only those papers published in English were considered.

### Study Selection

The study selection process in this review involved 3 main steps. First, we used EndNote to remove any duplicate studies from our search results. Then, the titles and abstracts of the remaining studies were screened to determine their relevance. For studies that passed this initial screening, a full-text review was conducted, during which the entire paper, including any supplementary materials, was carefully read. To ensure accuracy, 2 independent reviewers (MA and AN) conducted the study selection process. In cases of disagreement during title or abstract screening or full-text review, a third reviewer (AAA) was consulted to resolve the conflict. In addition, we calculated Cohen κ statistic to assess interreviewer agreement, which yielded a value of 0.78-0.83 by title or abstract screening or full-text review—indicating a high level of consistency and reliability in the data selection process [20].

### Data Extraction

To ensure a structured and consistent approach to data extraction, we developed a data extraction form, which was pilot-tested using 5 selected studies before full implementation. This form was designed to capture key details related to the study characteristics, datasets, features, and AI methodologies. The finalized data extraction form used in this review is shown in Multimedia Appendix 2. Two independent reviewers (MA and AN) used Microsoft Excel to extract data systematically. Any discrepancies between them were resolved through discussion.

### Data Synthesis

We analyzed the extracted data using a narrative synthesis approach, summarizing key findings in descriptive text and tables to provide a clear overview of the research. First, we outlined the basic details of each study, including the year of publication and the country where the research was conducted. Subsequently, we characterized the datasets underpinning AI model development, cataloged the AI methodologies used in each study, and detailed the feature attributes used in model construction. To keep the process structured and ensure accuracy, we used Microsoft Excel to organize and synthesize the extracted data efficiently.

## Results

### Search Results

**Figure 1.**
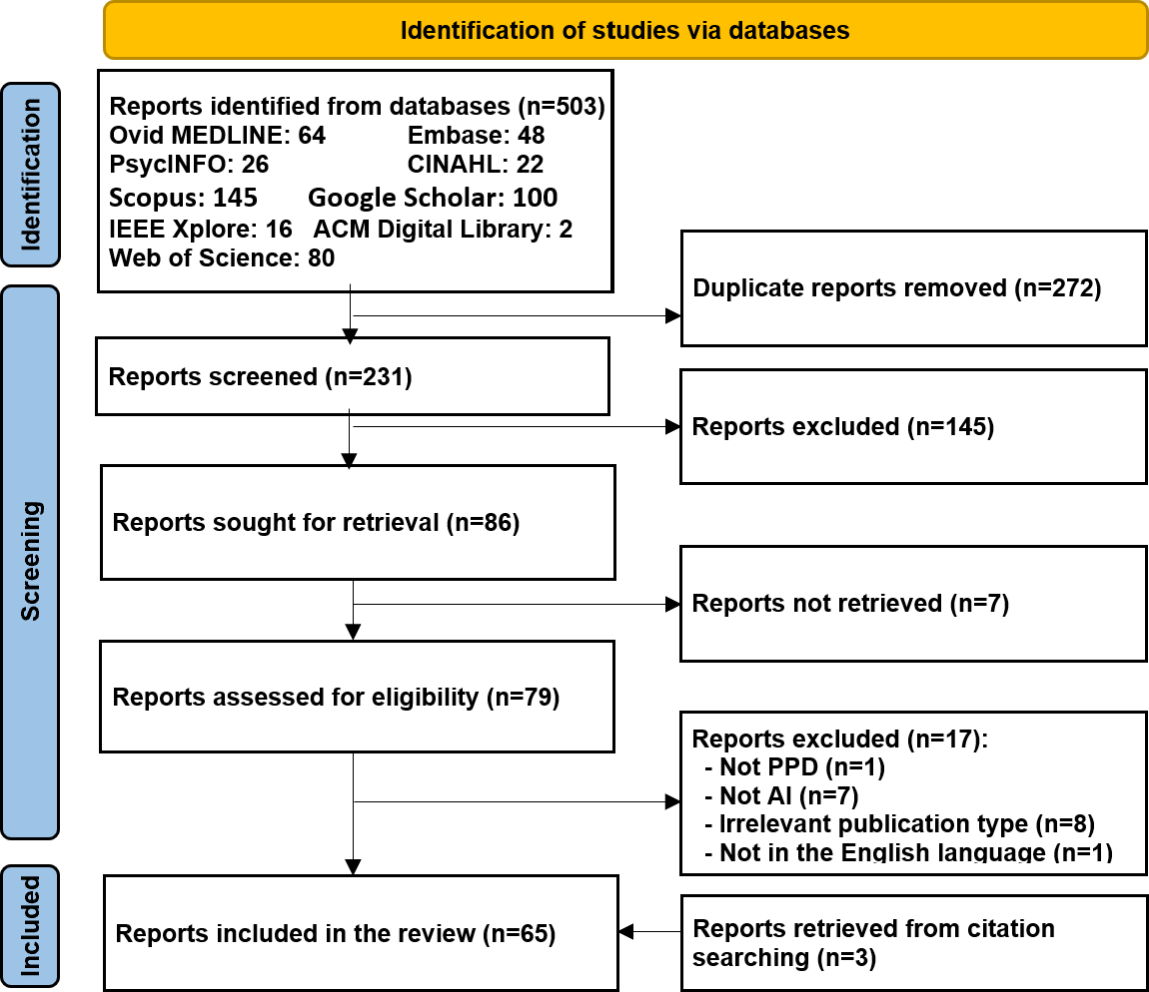
PRISMA (Preferred Reporting Items for Systematic Reviews and Meta-Analyses) 2020 flow diagram illustrating the study selection process. AI: artificial intelligence; PPD: postpartum depression.

As illustrated in Figure 1, a total of 503 records were retrieved through searches across 9 databases: Ovid MEDLINE (n=64), Embase (n=48), PsycINFO (n=26), CINAHL (n=22), IEEE Xplore (n=16), ACM Digital Library (n=2), Scopus (n=145), Web of Science (n=80), and Google Scholar (n=100, limited to the top 100 results ranked by relevance). After removing 272 duplicate records using reference management software, 231 unique reports remained for screening. After reviewing the titles and abstracts, 145 records were excluded. The full texts of the remaining 86 records were retrieved for further assessment. Of these, 7 full-text papers were not available. After evaluating the 79 available full-text papers, 17 studies were excluded for the following reasons: they did not use AI (n=7); did not focus on PPD (n=1); were not journal papers, conference papers, or dissertations (n=8); or were not written in English (n=1). Three additional relevant studies were identified through both backward and forward reference list screening. Ultimately, 65 studies were included in this review [21-85].

### Characteristics of the Included Studies

As shown in Table 1, the included studies were published between 2009 and 2025, with the highest number of publications occurring in 2024 (26.1%). Regarding publication types, the majority were journal papers (70.8%). The United States contributed the largest proportion of studies (27.7%), followed by China (15.4%) and Bangladesh (13.9%). This review included 39 out of 65 (60%) retrospective studies and 27 out of 65 (41.5%) prospective studies. The number of participants in the included studies ranged from 11 to 573,634, with a mean of 18,187.4 (SD 72,933.8). Participant distribution was as follows: 23 studies had fewer than 500 participants, 27 studies included between 500 and 5000 participants, 8 studies had between 5001 and 50,000 participants, and 7 studies involved more than 50,000 participants. Among the 26 studies that reported mean participant age, values ranged from 26 to 44.5 years, with an overall mean average of 31.08 (SD 3.42) years. See Multimedia Appendix 3 for overall information about included studies.

**Table 1.** Characteristics of the included studies.

Key aspects	Studies	References
Year of publication, n/N (%)		
2025	3/65 (4.6)	[30,46,77]
2024	17/65 (26.1)	[21,24,35,37,40,41,43,45,51,52,65-67,69,78,83,85]
2023	14/65 (21.5)	[28,32,33,42,44,50,56,63,68,72-74,79,80]
2022	10/65 (15.4)	[34,36,39,47,58-62,81]
2021	5/65 (7.7)	[22,26,48,55,84]
2020	6/65 (9.2)	[23,31,57,64,71,82]
2019	4/65 (6.2)	[25,29,49,76]
2018	3/65 (4.6)	[27,54,75]
≤2017	3/65 (4.6)	[38,53,70]
Publication type, n/N (%)		
Journal paper	46/65 (70.8)	[22,23,25,28-31,33,34,37-44,46,47,49-52,54,55,57,60,62-65,67-70,74,76-85]
Conference paper	18/65 (27.7)	[21,24,26,27,32,35,45,48,53,56,58,59,61,66,71-73,75]
Dissertation	1/65 (1.5)	[36]
Country of publication, n/N (%)		
United States	18/65 (27.7)	[24,25,31,37,39,41,43,51,53,55,57,62,64,74,76,80,83,84]
China	10/65 (15.4)	[27,42,63,69,75,77-79,82,85]
Bangladesh	9/65 (13.9)	[21,35,40,45,52,61,65,67,73]
India	6/65 (9.2)	[32-34,48,56,66]
Japan	3/65 (4.6)	[46,47,81]
United Kingdom	2/65 (3.1)	[44,71]
Spain	2/65 (3.1)	[38,70]
Sri Lanka	2/65 (3.1)	[58,59]
Others^a^	13/65 (1 each) (20)	[22,23,26,28-30,36,49,50,54,60,68,72]
Research design^b^, n/N (%)		
Retrospective	39/65 (60)	[21,23-27,30,32,35,37,40-45,47-49,51-56,60,61,64-66,71-76,80,83,84]
Prospective	27/65 (41.5)	[22,28,29,31,33,34,36,38,39,46,50,51,57-59,62,63,67-70,77-79,81,82,85]
Number of participants		
Mean (SD)	18,187.4 (72,933.8)	[21-85]
Range	11‐573,634	[21-85]
<500	23/65 (35.4)	[24,26-28,30,31,33,34,37,48,49,53,54,57,61,66,68,71,72,77,79,81,85]
500‐5000	27/65 (41.5)	[21,22,25,29,35,38-42,45,50,52,58-60,62,63,65,67,69,70,73,75,78,80,82]
5001‐50,000	8/65 (12.3)	[32,36,47,51,56,64,74,76]
>50,000	7/65 (10.8)	[23,43,44,46,55,83,84]
Mean age (years)^c^		
Mean (SD)	31.08 (3.42)	[21-85]
Range	25.99‐44.5	[21-85]

a Others include Australia, Brazil, Indonesia, Italy, Mexico, Nigeria, Norway, Pakistan, Palestine, Portugal, Saudi Arabia, Slovenia, and Sweden.

b The number of studies does not add up as one study used both retrospective and prospective designs.

c Mean age not reported: 39 studies (60%).

### Characteristics of Datasets

As depicted in Table 2, the average dataset size was 37,338.5 (SD 160,309.6), with a range spanning from 16 to 1,170,446. The dataset size fell between 500 and 5000 in 28 out of 65 (43.1%) studies. Most studies (49/65, 75.4%) used closed-source data, while the remaining (16/65, 24.6%) relied on open-source datasets. The studies included various data formats, including textual, tabular, audio, video, and images. The majority (57/65, 87.7%) used a single data format (unimodal), while the remaining studies (8/65, 12.3%) integrated multiple data formats (multimodal). The survey was the most common data collection approach (50/65, 76.9% of studies), followed by data sourced from EHRs (25/65, 38.5% of studies). Most studies (45/65, 69.2%) were conducted in health care settings. Regarding the timing of outcome measurement for PPD, 20 out of 65 (30.8%) studies assessed outcomes within 12 weeks of delivery (short term), 14 out of 65 (21.5%) studies between 12 and 36 weeks (medium term), and 17 out of 65 (26.2%) studies after more than 36 weeks (long term). The most common reference standard used for labeling the data (outcomes) was the EPDS (37/65, 56.9% of studies). Further details on the characteristics of the datasets used in the included studies are provided in Multimedia Appendix 4.

**Table 2.** Characteristics of datasets used in the included studies.

Data summary	Studies, n (%)	References
Dataset size^a^		
Mean (SD)	37,338.5 (160,309.6)	[21-85]
Range	16‐1,170,446	[21-85]
Dataset size categories, n/N (%)		
<500	20/65 (30.8)	[24,26,28,30,33,34,37,48,49,53,54,57,61,66,68,71,72,79,81,85]
500‐5000	28/65 (43.1)	[21,22,25,29,31,35,38-42,45,50,52,58-60,62,63,65,67,69,70,73,77,78,80,82]
5001‐50,000	8/65 (12.3)	[32,36,47,56,64,74-76]
>50,000	9/65 (13.9)	[23,27,43,44,46,51,55,83,84]
Data source		
Closed	49/65 (75.4)	[22-27,29-32,36-39,42-44,46-51,53-55,57-63,68-72,74-79,81-85]
Open	16/65 (24.6)	[21,28,33-35,40,41,45,52,56,64-67,73,80]
Data format^b^		
Unimodal	57/65 (87.7)	[21-26,29,30,32,34-40,42-50,52,54-66,68-76,79-85]
Multimodal	8/65 (12.3)	[27,28,31,33,41,51,53,67]
Data collection methodology^c^		
Survey	50/65 (76.9)	[21,22,24-28,30,31,33-36,38,40-42,44-46,50-54,56-65,67-73,75,78-82,85]
EHRs^d^	25/65 (38.5)	[23,37,41-44,46,47,49,50,55,67-70,73,74,76-79,81,83-85]
Social media	8/65 (12.3)	[27,29,32,33,51,53,66,67]
Sensor-based	5/65 (7.7)	[37-39,44,49]
Laboratory-based data	2/65 (3.1)	[79,81]
Setting^c^		
Health care	45/65 (69.2)	[21-23,28,30,33-35,37-43,45-47,49-52,54,55,57-59,63,65,67-71,73-79,82-85]
Community	18/65 (27.7)	[25,27,29,31,32,36,48,51,53,56,60-62,64,67,72,80,81]
Academic	5/65 (7.7)	[24,26,37,44,66]
Outcome measurement timing (weeks)^e^		
Short term (<12)	20/65 (30.8)	[34,38,42,46,47,50,52,55,57,62,63,67,68,71,74,77,78,81,82,85]
Medium term (12‐36)	14/65 (21.5)	[36,46,50,57-59,62,63,70,72,78,80,81,85]
Long term (>36)	17/65 (26.2)	[22-24,36,37,43,48,53,57,63,64,69,76,80,83-85]
Reference standard		
EPDS^f^	37/65 (56.9)	[22,24,26-30,34,38-40,42,46,47,50,52,53,55-59,61-63,67-72,76-78,80-82]
*ICD*^g^	8/65 (12.3)	[2,23,29,41,42,44,49,76,83]
PHQ^h^	7/65 (10.8)	[3,34,43,60,61,64,67]
PDSS^i^	3/65 (4.6)	[4,25,34,48]
PPDS^j^	2/65 (3.1)	[5,32,67]

a Mean (SD) is calculated.

b The number of studies does not add up as some studies used multiple data collection methodologies.

c The number of studies does not add up as some studies are conducted in multiple settings.

d EHRs: electronic health records.

e The number of studies does not add up as the timing of outcome measurement varied across studies. Outcome measurement timing not reported: 27 (41.5%).

f EPDS: Edinburgh Postnatal Depression Scale.

g *ICD*: *International Classification of Diseases*.

h PHQ: Patient Health Questionnaire.

i PDSS: Postpartum Depression Screening Scale.

j PPDS: Postpartum Depression Scale.

### Characteristics of Preprocessing Techniques

Table 3 summarizes the most frequently used preprocessing techniques identified across the reviewed studies. Across the reviewed literature, feature transformation overwhelmingly dominates preprocessing: Min-Max scaling and Z score standardization appear in 78.5% (51/65) of studies. In contrast, missing data strategies remain underutilized—only 44.6% (29/65) of papers applied any form of imputation, leaving 33.8% (22/65) to rely on case deletion or ignore the issue entirely. Class imbalance remedies are similarly rare: just 4.6% (3/65) of studies used stratified resampling or SMOTE variants, while cost-sensitive learning appeared in only 6.2% (4/65).

Categorical encoding methods vary in popularity: label encoding leads at 29.2% (19/65), one-hot encoding in 13.9% (9/65), binary encoding in 9.2% (6/65), dummy encoding in 6.2% (4/65), and target encoding in a mere 3.1% (2/65). For feature selection, tree-based importance (Gini impurity) featured in 18.5% (12/65) of studies and Pearson correlation filtering in 12.3%, with recursive feature elimination (5/65, 7.7%), information-gain ratio (6.2%), and SHAP-based methods (4/65, 6.2%) trailing behind.

Finally, dimensionality reduction and specialized feature extraction remain fringe techniques: sequential floating forward selection was used in only 7.7% (5/65) of papers and principal component analysis (PCA) in 6.2% (4/65), text vectorization methods (eg, N-grams, TF-IDF) in 4.6% (3/65), domain-specific statistical features in 3.1% (2/65), and acoustic‐signal processing (MFCC) in just 1 study (1/65, 1.5%). For a comprehensive overview of dataset characteristics used in the studies, refer to Multimedia Appendix 5.

**Table 3.** Characteristics of datasets used in the included studies.

Preprocessing techniques	Studies, n/N (%)	References
Dimensionality reduction techniques		
Sequential floating forward selection and SHAP^a^	5/65 (7.7)	[51,56,71,75,82]
Principal component analysis	4 /65 (6.2)	[36,52,77,81]
Others (each one 1)^b^	1/65 (1.5)	[33,57,64,66,67,72]
Feature extraction^c^		
Psycholinguistic and N-gram text vectorization	3/65 (4.6)	[29,33]
Domain-specific statistical	2/65 (3.1)	[51,56]
Acoustic signal feature extraction	1/65 (1.5)	[31]
Feature selection		
Regularization of the model		
Pearson correlation	8/65 (12.3)	[33,36,41,44,45,48,54,61]
Spearman rank filtering	2/65 (3.1)	[58,59]
Chi-square independence test	2/65 (3.1)	[21,65]
Cox proportional hazards and Kaplan-Meier survival analysis	1/65 (1.5)	[24]
Wrapper and tree-based selection		
Gini importance/mean decrease in impurity	12/65 (18.5)	[22,23,25,40,46,52,71,73,82]
Recursive feature elimination with cross-validation	5/65 (7.7)	[30,40,64,73,74]
Entropy-based information gain ratio	4/65 (6.2)	[25,30,41,46]
SHAP value-based importance via differential evolution	4/65 (6.2)	[37,43,62,63]
Other metaheuristic and ensemble filters^d^	1/65 (1.5)	[64,71,75,84]
Encoding approaches		
Label encoding	19/65 (29.2)	[23-25,29,31,33,38,42,48,50,51,53,61,63,64,66-68,73]
One-hot encoding	9/65 (13.9)	[26,27,32,36,41,43,52,56,65]
Binary encoding	6/65 (9.2)	[22,34,40,50,74,76]
Dummy encoding	4/65 (6.2)	[47,70,78,84]
Target encoding	2/65 (3.1)	[41,59]
Handling unbalanced data		
Manual resampling	15/65 (23.1)	[23,29,31,47,50,52,53,56,58,60,61,64,70,71,73]
Class weighting and cost-sensitive	4/65 (6.2)	[55,63,69]
Random oversampling (eg, SMOTE^e^ variants)	3/65 (4.6)	[36,56,76]
Handling missing data		
Statistical imputation (mean/median/KNN^f^/MICE^g^)	29/65 (44.6)	[22,24,31,33,34,36,38,41,43,44,46,51-54,58-60,62,65-67,70,73,78,80,82,84,85]
Complete case analysis (listwise deletion)	22/65 (33.8)	[23,27-29,34,36-38,40,45,47,50,51,54,64,74-79,81]
Feature transformation techniques		
Min–max scaling and Z-score standardization	51/65 (78.5)	[22-24,29-32,34,36-38,40,41,43,49-52,54,56,58,59,67-71,74-76,78-82,84,85]
Text tokenization, lemmatization, and stop-word removal	23/65 (35.4)	[26,27,29,32,33,36,40,41,47,50-52,64,65,67,72]
Log/power transforms (Box-Cox, Yeo-Johnson)	9/65 (13.8)	[25,31,40,43,45,46,66,70]
Polynomial and interaction feature generation	9/65 (13.8)	[25,31,40,43,45,46,66,70]

a SHAP: SHapley Additive exPlanations to interpret model.

b Others include linear discriminant analysis (LDA), t-SNE, latent semantic analysis (LSA), latent Dirichlet allocation (LDA), spatial feature extraction, and relief algorithm.

c Psycholinguistic text vectorization includes N-gram characteristics; linguistic inquiry and word count for emotion, cognition, social content; LDA topics; and TF-IDF. Acoustic signal feature extraction includes MFCC, spectral contrast, and chroma.

d Others include bagging-based selection-by-filter methods, sequential floating forward selection, sequential forward selection, relief algorithm, and Boruta algorithm.

e SMOTE: Synthetic Minority Oversampling Technique.

f KNN: K-Nearest neighbor algorithm.

g MICE: Multiple Imputation by Chained Equations to handle missing data.

### Characteristics of Features Used in Included Studies

The reviewed studies incorporated 9 distinct categories of data in AI model development (Table 4). Sociodemographic data were most frequently used (49/65, 75.4% of studies), followed by psychological data (44/65, 67.7% of studies), obstetric data (36/65, 55.4% of studies), and behavioral data (23/65, 35.4% of studies). The number of features used varied significantly across studies, ranging from 2 to 988, with a mean average of 44.88 (SD 129.72). Notably, nearly two-thirds of the studies (43/65, 66.2%) used fewer than 26 features.

Within each data type, the most commonly used individual features were age for sociodemographic data (37/65, 56.9% of studies), mode of delivery for obstetric data (15/65, 23.1% of studies), maternal anxiety for psychological data (13/65, 20% of studies), breastfeeding status for behavioral data (11/65, 16.9% of studies), linguistic inquiry and word count (LIWC) features—such as positive emotions (“happy”), cognitive processes (“think”), and personal pronouns (“I” and “we”)—for linguistic data (11/65, 16.9% of studies), metabolic pathways and circulating markers for biomarker data (4/65, 6.2% of studies), newborn gender for neonatal data (11/65, 16.9% of studies), hypertensive disorders for medical history data (11/65, 16.9% of studies), and tweet metadata for sensor-based data (3/65, 4.6% of studies). Additional characteristics of the datasets used in the reviewed studies are shown in Multimedia Appendix 6.

**Table 4.** Characteristics of features used in the included studies.

Features characteristics	Studies, n/N (%)	References
Data type^a^		
Sociodemographic	49/65 (75.4)	[21-24,28,30-32,34-36,38,40-50,52,53,55,56,58-65,67-71,74,76-78,80,82-85]
Psychological	44/65 (67.7)	[21-24,26,28-30,34-36,38,40-46,48,50-54,57,60-65,67,70-74,76,78,80,82-84]
Obstetric	36/65 (55.4)	[22-24,30,34,36,41,43,44,46-49,51,55-61,63,64,67-71,74,76-78,80,83-85]
Behavioral	23/65 (35.4)	[22,25,32,34,36,42-44,47,50,51,53,55,56,58-61,63,64,67,80,82]
Medical history	17/65 (26.2)	[22,23,43,46,47,49,56,63,67,68,71,76,78,80,83-85]
Neonatal	16/65 (24.6)	[22,28,30,38,47,50,53,56,61,63,64,70-72,78,85]
Linguistic	9/65 (13.9)	[26,27,29,31,33,39,66,67,75]
Biomarkers	7/65 (10.8)	[46,57,68,69,77,79,81]
Sensor-based	5/65 (7.7)	[37,44,51,60,66]
Number of features^b^		
Mean (SD)	44.9 (129.7)	[21-85]
Range	2‐988	[21-85]
Feature range		
≤25	43/65 (66.2)	[21,23-29,31-33,35-38,40-42,45,49-52,54-60,65-71,73,74,78,79,82,85]
26‐50	16/65 (24.6)	[22,30,34,43,47,48,53,61,63,64,72,76,77,80,83,84]
>50	6/65 (9.2)	[39,44,46,62,75,81]
Data input features sociodemographic data^a^		
Age	37/65 (56.9)	[21-24,28,30-32,34,35,38,40,41,43,45,47-50,52,55,58-61,63-65,68-71,74,77,80,82,85]
Education level	21/65 (32.3)	[22,24,28,30,32,34,36,41,46,48,58-61,63,64,68,74,80,82,85]
Marital status	20/65 (30.8)	[22,30,34,41,43,46-48,53,58,59,61,63,64,68,70,76,83-85]
Monthly income	13/65 (20)	[30,34,36,38,41,50,60,61,64,70,78,82,85]
Employment status	11/65 (16.9)	[22,28,30,43,46,48,61,63,64,70,85]
Obstetric data^a^		
Mode of delivery	15/65 (23.1)	[30,32,34,41,47,48,55,61,68,74,78,80,83-85]
Parity	11/65 (16.9)	[22,30,36,43,46,63,64,68,78,80,85]
Gestational age	9/65 (13.9)	[24,30,34,47,49,61,68,71,78]
Gravida	7/65 (10.8)	[24,30,49,60,68,83,84]
Obstetric complications	6/65 (9.2)	[23,34,43,61,69,85]
Psychological data^a^		
Maternal anxiety	13/65 (20)	[21,25,27,35,40,45,48,52,62,65,71,76,83]
Depression history	12/65 (18.5)	[22,30,34,41,43,44,48,53,55,63,69,82]
Feeling of guilt	9/65 (13.9)	[21,25,27,35,40,45,52,65,73]
Feeling sad	8/65 (12.3)	[21,27,35,40,45,52,54,65]
Sleeping disorders	7/65 (10.8)	[25,27,46,47,53,54,62]
Behavioral data^a^		
Breastfeeding status	11/65 (16.9)	[23,28,34,47,48,53,56,61,64,78,85]
Problems bonding with baby	9/65 (13.9)	[21,35,40,45,48,52,61,65,73]
Planned pregnancy	8/65 (12.3)	[32,34,48,53,61,74,80,85]
Smoking status	7/65 (10.8)	[22,23,46,47,60,63,64]
Alcohol use	6/65 (9.2)	[22,36,46,47,63,64]
Linguistic data^a^		
LIWC^c^ features	11/65 (16.9)	[27]
Speech and acoustic	8/65 (12.3)	[31]
Emotional and cognitive expression	4/65 (6.2)	[33]
Language models	2/65 (3.1)	[39]
Tweet attributes language	2/65 (3.1)	[66]
Biomarker data^a^		
Metabolic pathway	4/65 (6.2)	[81]
Circulating biomarkers	4/65 (6.2)	[46,57,68]
Neurological	3/65 (4.6)	[69,79]
Protein-related	2/65 (3.1)	[77]
Genetic/epigenetic	1/65 (1.5)	[57]
Neonatal data^a^		
Newborn gender	11/65 (16.9)	[30,31,38,47,50,58,59,61,64,70,85]
Birth weight	8/65 (12.3)	[30,34,41,47,64,71,77,78]
Preterm birth	6/65 (9.2)	[43,48,58,59,76,77]
Health of baby	4/65 (6.2)	[28,30,53,85]
Apgar scores	3/65 (4.6)	[47,71,78]
Medical history^a^		
Hypertension disorders	111/65 (16.9)	[22,43,47,49,56,63,76,78,80,83,84]
Gestational diabetes	44/65 (6.2)	[43,49,78,80]
Migraine	44/65 (6.2)	[22,63,83,84]
Preeclampsia	33/65 (4.6)	[43,83,84]
Hypothyroidism	33/65 (4.6)	[83-85]
Sensor-based^b^		
Tweet metadata	33/65 (4.6)	[66]
Activity intensity	33/65 (4.6)	[37,60]
Calories burned	11/65 (1.5)	[37]
Heart rate	11/65 (1.5)	[37]

a The number of studies does not add up as certain features are reported in multiple studies within each category, resulting in repeated counts.

b The sensor-based category includes only 4 features.

c LIWC: linguistic inquiry and word count.

### Characteristics of AI Techniques

As shown in Table 5, the most included studies (52/65, 80%) used AI models for predicting PPD (ie, identifying women at risk of developing PPD in the future), while 14 out of 65 (21.5%) studies used them for detection (ie, identifying whether a woman is currently experiencing PPD). Most studies leveraged ML techniques (57/65, 87.7%), whereas DL techniques were applied in 11 out of 65 (16.9%) studies. The predominant application of AI models was in classification tasks (eg, identifying the presence, absence, or severity level of PPD). In contrast, 5 out of 65 (7.7%) studies used AI models for regression tasks (eg, detecting EPDS score). Various AI algorithms were used in the included studies, with random forest (RF) being the most common (29/65, 44.6%), followed by support vector machine (26/65, 4%) and logistic regression (LogR) (23/65, 35.4%).

The most frequently used optimization strategy among the included studies was stochastic gradient descent (9/65, 13.9%), followed by Adam (7/65, 10.8%) and learning rate scheduling (6/65, 9.2%). The most applied regularization and model stabilization techniques were L1/L2 regularization (9/65, 13.9%) and grid search (9/65, 13.9%). To validate AI model performance, both k-fold cross-validation and holdout validation were the most widely adopted approaches (32/65, 49.2%). Accuracy was the most reported performance metric (49/65, 75.4%), followed by sensitivity (48/65, 73.9%) and area under the curve (AUC) (41/65, 63.1%). Additional characteristics of the Model of Characteristics of AI Techniques used in the reviewed studies are shown in Multimedia Appendix 7.

**Table 5.** Characteristics of artificial intelligence techniques used in the included studies.

Characteristics	Studies, n/N (%)	References
AI^a^ algorithm aim		
Prediction	52/65 (80)	[21-27,29,30,32-36,39-47,49,50,52-55,57-60,62,64-69,72-74,76-78,80-85]
Detection	14/65 (21.5)	[28,31,37,38,48,51,56,61,63,69-71,75,79]
AI category		
Machine learning	57/65 (87.7)	[21-25,29-39,41-62,64,66-70,73-85]
Deep learning	11/65 (16.9)	[26-28,32,40,41,58,59,63,65,71]
Transfer learning	3/65 (4.6)	[26,40,41]
Natural language processing	3/65 (4.6)	[33,39,72]
Reinforcement learning	2/65 (3.1)	[63,69]
Problem solving approach		
Classification	60/65 (92.3%)	[21-35,37-43,45-53,55-67,69-76,78-85]
Regression	5/65 (7.7%)	[36,44,54,68,77]
AI model type		
Ensemble methods		
Bagging		
Random forest	29/65 (44.6)	[21,24,33,34,37,41,43,47,48,51,52,55,56,58-61,64,65,71,73,74,76,78,80-82,84,85]
Bagging	1/65 (1.5)	[48]
Extreme random trees	1/65 (1.5)	[73]
Extra trees	1/65 (1.5)	[22]
Boosting		
XGBoost	15/65 (23.1)	[21,24,30,36,41,43,45,55,61,65,68,76,80,84,85]
Gradient boosting	10/65 (15.4)	[22,23,30,51,56,60-62,68,73]
AdaBoost	9/65 (13.9)	[33,41,45,48,53,56,64,65,73]
CatBoost	6/65 (9.2)	[35,41,45,65,68,73]
LightGBM	4/65 (6.2)	[35,45,65,68]
Stacking		
Stacking ensemble	1/65 (1.5)	[21]
Stacking model	1/65 (1.5)	[52]
Nested stacking	1/65 (1.5)	[73]
Neural networks		
Multilayer perceptron	11/65 (16.9)	[43,52,56,67,70,84]
Recurrent neural	6/65 (9.2)	[26,27,40,41,66]
Convolutional neural	5/65 (7.7)	[28,31,36,64,80]
Natural language processing	1/65 (1.5)	[72]
Classification models		
Support vector machine	26/65 (40)	[21,26,29,32,33,37,38,43,47,49-51,53,56-61,64,75,76,78,79,82,85]
Decision tree	18/65 (27.7)	[21,24,25,30,41,46,48,49,51-53,56,60,65,76,78,84,85]
K-Nearest neighbors	9/65 (13.9)	[21,37,49,51,52,56,64,65,78]
Recursive partitioning	1/65 (1.5)	[64]
Probabilistic classification		
Logistic regression	23/65 (35.4)	[21,23,29,33,38,42-44,47,50,53,55,56,61,64,65,70,74,76,77,83-85]
Naive Bayes	7/65 (10.8)	[32,34,38,50,53,60,64]
Linear regression models		
Ridge regression	6/65 (9.2)	[22,34,44,47,52,68]
LASSO regression	5/65 (7.7)	[22,34,39,44,77]
Elastic net	5/65 (7.7)	[23,36,44,47,68]
Support vector regression	2/65 (3)	[68]
Kernel regression	1/65 (1.55)	[68]
Optimization strategies (gradient-based optimization)		
Stochastic gradient descent	9/65 (13.9)	[27-29,32,36,40,56,64,66]
Adam	7/65 (10.8)	[27,32,36,41,48,56,66]
Learning rate scheduling	6/65 (9.2)	[23,27,29,32,41,56]
AdamW	2/65 (3.1)	[28,40]
Cosine Annealing	2/65 (3.1)	[40,48]
Momentum	1/65 (1.5)	[36]
Regularization and model stabilization		
L1/l2 regularization	9/65 (13.9)	[23,28,29,36,42,44,76,77,83]
Grid search	4/65 (6.2)	[43,65,79,81]
Dropout	8/65 (12.3)	[27,36,40,41,48,56,59,64]
Batch normalization	3/65 (4.6)	[36,40,41]
Early stopping	2/65 (3.1)	[28,36]
Weight decay	2/65 (3.1)	[36,56]
Osprey optimization	1/65 (1.5)	[67]
Validation techniques		
K-fold cross-validation	32/65 (49.2)	[22,23,25,29,30,35,37,39,43,47-49,52,53,61-65,68,69,71,73-77,79,82-84]
Holdout validation	32/65 (49.2)	[21,24,26-29,31-34,36,38-43,45-47,50,51,53-56,58-60,70,78,81]
Leave-one-out cross-validation	1/65 (1.5)	[57]
Nested cross-validation	1/65 (1.5)	[44]
ML^b^ performance measures		
Accuracy	49/65 (75.4)	[21,22,24,26-35,38,40,41,43-46,48,49,52,54-67,69-71,73,74,76-80,82,85]
Sensitivity	48/65 (73.9)	[21,22,24-26,29-35,37,38,40-46,49-65,67,70,71,73,75,76,79,82-84]
AUC^c^	41/65 (63.1)	[22-25,31,34,37-51,53,55-57,59-61,64,70,74-84]
Precision	36/65 (55.4)	[21,22,24-26,29-35,37,40,41,43-45,50,53,56,57,59-62,64,65,67,73,78,82-85]
Specificity	23/65 (35.4)	[22,30,31,34,37,38,42,44-46,48,51,55,63,64,70,71,73,75,76,79,82,84]
Geometric mean	7/65 (10.8)	[38,50,51,61,62,69,70]
Negative predictive value	7/65 (10.8)	[22,32,34,44,45,82,84]
*F*_1_-score	5/65 (7.7)	[21,35,58,69,73]
Root mean squared error/mean squared error	3/65 (4.6)	[36,58,68]

a AI: artificial intelligence.

b ML: machine learning.

c AUC: area under the curve, ROC-AUC (receiver operating characteristic) that plots true-positive rate against false-positive rate at different threshold settings.

Among the AI models evaluated in Table 6, ensemble methods emerged as the top performers, with an average accuracy of 93.4%, an *F*_1_-score of 92.5%, and an AUC of 89.4%. Among gradient boosting techniques, CatBoost achieved the highest AUC of 98.6%, alongside robust accuracy and *F*_1_-score metrics. LightGBM also demonstrated strong performance, recording 92.6% accuracy, an *F*_1_-score of 87.8%, and an AUC of 91.1%, highlighting its scalability and effectiveness. XGBoost delivered competitive results, with an accuracy of 89.1% and an AUC of 86.8%. Convolutional neural networks (CNNs) showed excellent performance as well—particularly in accuracy (92%) and *F*_1_-score (95.1%)—although they were evaluated in a smaller subset of studies.

Traditional tree-based models, including RFs and recursive partitioning, also showed moderate to strong performance. RFs achieved an average accuracy of 80.5% and an AUC of 82.4%, while broader tree-based classifiers averaged 82.8% accuracy and 82.6% AUC. Recursive partitioning, however, showed lower accuracy (71.8%) and AUC (74.7%) across the few studies assessed.

Across all models included, the mean performance was 81.7% (SD 11.05) for accuracy, 80.51% (SD 15.44) for *F*_1_-score, and 81.0% (SD 12.0) for AUC. Collectively, these findings underscore the strong predictive capabilities of DL architectures and ensemble-based approaches—especially boosting models—in detecting PPD, consistently outperforming conventional ML algorithms across most evaluation metrics or detailed information on performance metrics (accuracy, *F*_1_-score, and AUC) (Multimedia Appendix 8).

**Table 6.** Accuracy, *F*_1_-score, and area under the curve of artificial intelligence models used in postpartum depression prediction.

Metrics^a^	Accuracy			*F*_1_-score			AUC^b^		
Model	Studies, n	Mean (SD)	Range	Studies, n	Mean (SD)	Range	Studies, n	Mean (SD)	Range
Random forest	23	80.5 (9.2)	59-96	19	80.9 (13.7)	39.3-95	25	82.4 (9.2)	65.1-98
Decision trees	16	82.8 (9.1)	68.8-98.1	12	83.9 (10.5)	66-98.58	13	82.6 (7.9)	69-97.6
XGBoost	13	89.1 (10.3)	67.6-100	8	91.8 (11.4)	66-100	7	86.8 (11.1)	73-100
LightGBM	6	92.6 (11)	70.2-98.4	6	87.8 (22.7)	41.6-98.6	4	91.1 (12.3)	72.7-98
AdaBoost	7	78.7 (9.6)	66-94	6	75.4 (11.6)	58-89	6	78.5 (5.7)	69-85.7
CatBoost	5	93.6 (9.5)	77-99.46	5	90.5 (11.1)	72-99.1	3	98.6 (0.5)	98-99
Gradient Boosting	8	79.2 (9.5)	67-79	7	76.7 (15.6)	45-92	10	86.8 (10.4)	70-97.3
Linear regressions	8	70.9 (4.9)	67-79	2	76.5 (0.7)	76-77	14	77.2 (5.3)	67-87
Logistic regressions	16	75.3 (7.3)	65.5-94.3	9	72.1 (16)	38.2-94.1	21	81.9 (9.6)	69.6-97
Naive Bayes	9	74 (6.6)	67.5-86.4	18	73.4 (13.2)	56-88.7	10	76.8 (7.1)	65.6-92
SVMs^c^	18	80 (8.4)	64-94.9	13	77.5 (14.1)	42.2‐94	18	78.7 (6.9)	64.2-90.3
KNNs^d^	8	79.5 (13)	61.5‐97	8	78.3 (13.9)	57 - 97	7	79.3 (9.5)	61.5-88.2
Neural networks	3	79.6 (14.4)	65-93.75	4	80.1 (15.1)	65.1-95.2	6	64.8 (24.8)	31.2-90.8
MLPs^e^	9	81.7 (8.3)	68-92	3	73.6 (24)	40.6-91.7	12	74.9 (17.3)	31.2-91.2
ANNs^f^	6	85.3 (10.8)	70.7-97.1	3	85.9 (12.3)	71.7-93	3	70.6 (6.3)	66-77.79
CNNs^g^	5	92 (8.1)	77.3-100	4	95.1 (3.9)	91.1-100	N/A^h^	N/A	N/A
Reinforcement learning	3	85.4 (4.1)	81-89.07	3	84.2 (4.7)	79.2-88.4	7	86.6 (3.8)	83-90.66
Ensemble models	9	93.4 (10.8)	65-99.84	9	92.5 (15.3)	52-99.21	7	89.4 (16)	56.5-98.95
Overall	174	81.7 (11.1)	59-100	141	80.5 (15.4)	38.2-100	176	81.0 (12)	31.2-100

a Models are grouped into tree-based, boosting, probabilistic, traditional machine learning, neural networks, and ensembles. Metrics are mean (SD). Study counts refer to the number of models reporting accuracy, *F*_1_-score, or AUC—not the number of references.

b AUC:area under the curve.

c SVMs: Support Vector Machines.

d KNNs: K-Nearest Neighbor algorithm.

e MLPs: multilayer perceptrons.

f ANNs: Artificial Neural Network.

g CNNs: Convolutional Neural Networks.

h N/A: not applicable.

## Discussion

### Principal Findings

This scoping review examines the evolving application of AI in PPD research, with approximately 80% of studies prioritizing early prediction over detection. This reflects a growing awareness of AI’s potential to enable proactive mental health interventions.

ML algorithms dominated (87.7%), suggesting a preference for structured data handling and model interpretability. Classical models such as RF (44.6%), LogR (35.4%), and XGBoost (23.1%) were especially prevalent, likely due to their ease of implementation, strong performance on tabular datasets, and alignment with the interpretability demands in health care as these models are well suited for structured and tabular clinical data (eg, demographics, EPDS scores, and EHR) and offer high interpretability, a core requirement in health care settings for clinical transparency and trust. In contrast, DL approaches—while more capable of handling complex, high-dimensional inputs—were used in only 16.9% of studies, indicating underutilization of architectures such as convolutional or recurrent neural networks (RNNs). This aligns with the nature of DL methods (CNNs, RNNs, and transformers) that require large, high-dimensional datasets (eg, text, electroencephalogram, and sensor signals), which were rare in the reviewed studies; also, DL models are less interpretable, a major limitation in mental health where explainability is critical for clinician adoption. NLP and reinforcement learning (RL) were rarely used, despite their potential for analyzing unstructured clinical notes and dynamic decision-making, respectively. This aligns with the fact that NLP is suitable for analyzing unstructured clinical notes, patient narratives, or social media data, and few studies had access to such datasets. In addition, RL’s strength in dynamic decision-making (eg, treatment adjustment over time) is difficult to apply in static, retrospective datasets common in PPD research.

More than 90% of studies focused on classification tasks—categorizing individuals as at risk or not for PPD—while only a few adopted regression models to estimate continuous risk levels. Although classification supports clinical decision-making, regression can offer more granular risk assessments, useful for personalized interventions and monitoring symptom trajectories. This aligns with real-world clinical workflows—binary screening tools are common. However, the underuse of regression models (predicting continuous risk) limits personalization and longitudinal risk tracking.

The optimization strategies such as Adam or stochastic gradient descent optimizers, learning rate scheduling, and dropout were rarely reported across the literature. This may reflect that most studies used classical ML, which does not require such parameters or limited technical expertise or a focus on classical methods over deep architectures. Regularization practices such as L1/L2 penalties, batch normalization, and early stopping were underused, despite their importance for model generalizability and performance stabilization. These techniques help prevent overfitting and improve generalizability. Their absence may reflect limited ML maturity or reliance on default model settings without tuning. Moreover, model validation techniques varied considerably. Although nearly half of the reviewed studies used k-fold cross-validation or holdout validation, external validation was seldom implemented. External validation requires access to independent datasets, which are often unavailable due to privacy constraints in mental health and raising concerns about generalizability. Performance evaluation also lacked consistency, with accuracy (75.4%) and sensitivity (73.9%) reported most frequently, while key metrics such as specificity, AUC, and *F*_1_-score were less commonly disclosed, which are essential for imbalanced datasets such as PPD.

By considering the results of performance evaluation across accuracy, *F*_1_-score, and AUC, they indicate that ensemble models, especially boosting techniques such as CatBoost, LightGBM, and XGBoost, consistently outperformed other AI methods in predicting PPD. Their high accuracy and AUC reflect strong generalization and robustness, owing to their ability to iteratively correct misclassifications and capture complex, nonlinear patterns—particularly valuable in noisy, imbalanced health care datasets.

CatBoost led with an AUC of 98.6%, benefiting from its advanced handling of categorical variables and built-in overfitting control, making it highly suited for structured health data. LightGBM followed closely, offering high accuracy (92.6%) and efficiency due to its gradient-based sampling and fast training, making it ideal for large-scale or real-time applications. XGBoost also performed competitively (89.1% accuracy and 86.8%) and remains popular for its transparency and feature importance tools.

In contrast, traditional decision tree–based classifiers such as RFs and generic tree–based models achieved moderate performance, with accuracy ranging from 80.5% to 82.8% and AUC values near 82%. While interpretable and computationally efficient, these models lacked the advanced learning mechanisms of boosting methods. Recursive partitioning methods, evaluated in only 2 studies, performed the weakest among tree-based approaches.

Overall, the mean model performance—accuracy: 81.7% (SD 11.05), *F*_1_-score: 80.51% (SD 15.44), and AUC: 81.0% (SD 12.0)—shows variability likely due to differences in datasets, preprocessing, and validation strategies. This underscores the need for standardized evaluation and external validation to ensure model reproducibility and clinical reliability. Also, this suggests limited awareness or inconsistent standards in performance reporting and hinders meaningful comparisons and meta-analysis across studies.

This inconsistency complicates comparative assessments across models and highlights the need for standardized evaluation frameworks.

This scoping review offers the first comprehensive synthesis of both foundational and advanced preprocessing techniques used in AI-driven PPD studies. The high prevalence of basic normalization methods—such as Min-Max scaling and Z-score standardization, reported in 78.5% of studies—demonstrates a broad consensus on the need to standardize input features, particularly in ML models sensitive to feature magnitude. These practices are foundational for ensuring convergence stability and improving model performance, especially in algorithms such as LogR and k-nearest neighbors. However, more advanced preprocessing techniques were markedly underutilized, limiting the full potential of AI in PPD prediction.

For example, although missing data are ubiquitous in real-world health care datasets, only 44.6% of studies applied imputation techniques to address it. The remaining studies either dropped missing values or excluded incomplete cases—approaches that risk reducing sample size and introducing systematic bias, particularly in psychiatric populations where follow-up and self-report compliance can vary. Likewise, class imbalance, a well-documented issue in mental health data (eg, more controls than PPD cases), was insufficiently addressed: only 23.1% of studies used resampling methods such as SMOTE, and an even smaller fraction (6.2%) incorporated cost-sensitive learning, which could improve model fairness and reduce false negatives—an important consideration in screening contexts.

In terms of categorical variable processing, label encoding (29.2%) and one-hot encoding (13.9%) were commonly used. While these methods are simple to implement, they may introduce ordinal bias or dimensionality explosion, respectively. More efficient encoding schemes (eg, target encoding and frequency encoding) that better preserve categorical relationships were rarely used, reflecting either limited awareness or concerns over interpretability.

Advanced feature engineering and selection techniques were also underexploited. Tree-based feature selection was used in only 18.5% of studies, despite its use in identifying nonlinear relationships and reducing overfitting. Dimensionality reduction methods such as PCA (6.2%) and interpretability tools such as SHAP (6.2%) were seldom implemented, limiting transparency and the ability to uncover key risk factors. Furthermore, multimodal or unstructured data processing techniques—including text or acoustic feature extraction—were applied in fewer than 5% of studies, despite their relevance in analyzing patient interviews, social media posts, or voice biomarkers.

In summary, while basic preprocessing steps have become standard practice, the limited adoption of more sophisticated strategies reflects a missed opportunity to enhance model robustness, generalizability, and interpretability. These gaps underscore the need for broader methodological literacy and the integration of more nuanced preprocessing pipelines tailored to the complexity and heterogeneity of PPD data.

Geographically, North America led the research landscape, with the United States contributing the largest share—just less than two-thirds. Asia also featured prominently, especially China, Bangladesh, and India. This wide participation demonstrates the global relevance and flexibility of AI solutions in diverse health care systems. However, it also reveals disparities in research capacity, underscoring the need for more contributions from underrepresented regions to ensure global equity. Bangladesh’s notable presence is largely due to the use of public datasets, showing how open access data can significantly influence research output. The included studies span from 2009 to 2025, with publication volume rising steadily. A quarter of the studies were published in 2024 alone, reflecting growing global interest, better access to digital health data, and advances in AI. The lower count from 2025 is likely due to early-year data collection. Most studies appeared in peer-reviewed journals (more than two-thirds), while fewer were conference papers (less than one-third), and only 1 was a dissertation—illustrating both the academic rigor and fast-evolving nature of this field. Sample sizes varied widely, ranging from 11 to 5,73,634 participants (mean 18,187.4), yet nearly half of the studies had fewer than 5000 participants, raising concerns about generalizability and model performance. Among the 26 studies reporting participant age, the average was 31.08 years—consistent with the typical childbearing population—although the lack of demographic transparency in many studies limits comparability and clinical applicability. Among studies reporting participant age, the mean average was 31.08 (SD 3.42) years, aligning with the typical reproductive age. However, more than half of the studies did not report age, limiting comparability and model applicability across age groups.

Sample sizes varied greatly—from less than a dozen to more than half a million—with most studies enrolling fewer than 5000 participants. Smaller studies often used surveys or interviews, while larger ones relied on national registries. This underscores the importance of large datasets, especially for training DL models. Closed-source datasets dominated, with only about 25% of studies using open data. This limits reproducibility and hinders collaboration. Expanding open access datasets and standardized repositories would improve transparency and accelerate innovation. Most studies were retrospective, drawing on accessible surveys and EHR data. While more than two-thirds used surveys and many depended on structured clinical inputs, a recent shift toward prospective designs reflects growing interest in real-time, high-quality data for AI validation. Use of social media and sensor data is emerging, indicating a move toward passive, continuous monitoring. However, objective biomarkers—such as hormonal, genetic, or neuroimaging data—were underutilized, appearing in only 2 studies, underscoring a missed opportunity for clinical robustness. Moreover, nearly 90% of studies used unimodal inputs (eg, surveys and EHRs), with few incorporating multimodal data such as text, audio, or imaging. This limits the ability to capture the complex biopsychosocial nature of PPD. Research predominantly occurred in health care settings, reflecting strong clinical relevance. Around one-third took place in communities and fewer than 10% in academic contexts. Expanding into diverse settings could improve the inclusivity and generalizability of AI-based PPD interventions. Assessment timing for PPD varied widely, with 12-month evaluations being most common. Studies spanned short-, medium-, and long-term intervals, yet more than 40% failed to specify timing—revealing a major gap in methodological transparency. This variability underscores both evolving perspectives on PPD progression and the need for standardized follow-up periods to enhance comparability, reproducibility, and clinical relevance of AI models. The EPDS is the most widely used reference in PPD research, followed by *International Classification of Diseases* (*ICD*) codes and PHQ-9, highlighting its strong validation in postpartum populations. However, inconsistent use of diagnostic tools across studies hampers comparability and a unified understanding of PPD. While AI models show promise using varied data sources, few are benchmarked against tools such as EPDS or PHQ-9—limiting assessments of their real-world clinical use.

Feature counts varied widely (2-988), with an average of 44.9; nearly two-thirds of studies used fewer than 25 features, indicating a preference for simplicity and interpretability. These findings underscore the need for broader adoption of sophisticated feature selection and dimensionality reduction techniques (eg, PCA, SHAP, and recursive elimination) to enhance predictive performance and clinical relevance. The results of this scoping review underscore the central role of sociodemographic features, which were the most frequently used across included studies on PPD. Variables such as age (56.9%), education level (32.3%), and marital status (30.8%) were among the most common predictors, highlighting the consistent reliance on structured patient-reported or administrative health records. Obstetric features were the second most common group, particularly mode of delivery (23.1%), parity (16.9%), and gestational age (13.9%). These findings align with literature suggesting that birth experience and maternal clinical history offer critical information in predicting PPD onset. Psychological indicators such as maternal anxiety (20%) and history of depression (18.5%) were also well represented. Their inclusion reflects growing interest in integrating mental health history and current affective symptoms into predictive frameworks. Similarly, behavioral factors such as breastfeeding status and bonding issues were frequently used to enhance emotional and functional contextualization of risk. Less frequently used were linguistic features (eg, LIWC metrics and emotional expression) and biomarkers (eg, epigenetic markers and neurological proteins), suggesting a growing but underutilized frontier. Notably, sensor-derived features (eg, tweet metadata, wearable-derived activity, or heart rate) appeared only in a handful of studies, despite the increasing ubiquity of digital health data. This spectrum of feature types illustrates a multidomain data integration trend, particularly among recent studies that incorporate EHRs, digital behavioral traces, and physiological data to enhance model robustness and precision.

### Comparison With Previous Reviews

The findings of this review are broadly consistent with earlier literature, including reviews by Kwok et al [10], Fazraningtyas et al [15], Qi et al [17], Saqib et al [18], Fazraningtyas et al [30], and Acharya et al [13]. These prior studies similarly identified a reliance on retrospective study designs, structured demographic and clinical features (eg, age, parity, and psychiatric history), and traditional ML models such as RFs, support vector machines, and LogR. Most were applied to survey-based or EHR-derived datasets, reflecting the accessibility and interpretability of structured data in maternal mental health contexts.

However, this scoping review extends the current literature in several important ways. First, our review is not focused narrowly only on model types or performance metrics; it even systematically maps the entire AI modeling pipeline, from data characteristics and preprocessing techniques to model training, optimization, and validation strategies. For example, while earlier studies acknowledged preprocessing in general terms, our analysis quantifies the usage of basic methods (eg, scaling and label encoding) and highlights the underuse of advanced techniques such as SMOTE, SHAP, recursive feature elimination, and cost-sensitive learning.

Second, this review identifies the limited adoption of advanced AI methodologies, including DL, transformer-based NLP, and transfer learning, despite their growing success in related health care domains. While Fazraningtyas et al [15] and Qi et al [17] recognized these tools conceptually, few studies in their datasets applied them operationally to PPD detection tasks—an observation confirmed and quantified by our analysis.

Third, our review offers a granular classification of more than 45 features across 9 thematic domains, revealing persistent dependence on sociodemographic and self-reported data. This pattern, while accessible and interpretable, introduces potential biases and limits the generalizability of models. In contrast, passive and objective inputs—such as biosensors, electroencephalogram, speech signals, or real-time behavioral metrics—remain substantially underused, despite their promise for early and noninvasive detection of PPD.

Fourth, unlike previous studies that typically summarized trends descriptively, this review visualizes and quantitatively tracks the growth of literature over time, the global distribution of research output, and the evolution of study design types, using structured frameworks and stacked visualizations. For instance, we highlight that Bangladesh’s growing presence is largely driven by the reuse of public datasets, illustrating how open data democratizes research participation.

Finally, this review distinguishes itself by its methodological scope and rigor. It includes studies published through February 2025 across 8 multidisciplinary databases, covering both prospective and retrospective designs, a wide range of countries, and diverse data sources. This comprehensive coverage enables a more nuanced understanding of current capabilities and persistent gaps in AI-based maternal mental health research. In particular, our audit of model regularization, hyperparameter tuning, and evaluation practices offers insight into areas often overlooked by earlier reviews. Taken together, these contributions provide a stronger foundation for the development of transparent, reproducible, and clinically relevant AI tools in PPD research—addressing both methodological blind spots and equity concerns raised in prior literature.

### Implication and Further Works

To significantly advance the field of AI-driven PPD research, several key strategic priorities should be addressed. These priorities focus on improving methodological rigor, inclusivity of data, clinical applicability, and ethical implementation.

First, enhance the integration of multimodal and objective data sources. Currently, research predominantly relies on traditional sociodemographic and self-reported survey data. There is an urgent need to incorporate underutilized modalities such as linguistic data (eg, LIWC, sentiment analysis, and acoustic speech patterns), biosignals (eg, heart rate variability and activity monitoring), wearable technology outputs, and biological biomarkers (eg, hormonal, metabolic, genetic, or epigenetic markers). Leveraging these richer data types can significantly enhance the accuracy, personalization, and early detection capabilities of predictive models.

Second, expand and prioritize sharing of open access datasets. Only about 25% of studies included in our review used publicly available datasets, highlighting a substantial barrier to reproducibility, benchmarking, and international collaboration. Developing standardized, large-scale, anonymized datasets should become a priority. Techniques such as federated learning could facilitate collaborative research across different institutions while maintaining data privacy and security.

Third, increase the adoption of longitudinal and prospective research designs. Most existing AI models for PPD prediction are based on retrospective or immediate postpartum data, limiting their ability to capture evolving symptom patterns over time. Incorporating longitudinal data collection into future studies is essential to better understand symptom progression, delayed onset, and relapse scenarios, thus enhancing the clinical relevance and predictive accuracy of AI models.

Fourth, advance multimodal fusion frameworks. Given the complexity of PPD, future models must systematically integrate structured data (eg, EHRs) and unstructured inputs (eg, text, audio, and sensor signals). Developing robust multimodal fusion approaches that effectively combine diverse data sources will significantly enhance model interpretability, clinical effectiveness, and predictive power.

Fifth, standardize preprocessing and feature engineering pipelines. Variability and incomplete reporting in preprocessing methods currently limit model comparability and reproducibility. Adopting standardized protocols for data preprocessing—including feature extraction, transformation techniques, and class imbalance adjustments (eg, SMOTE and cost-sensitive learning)—is necessary. Transparent reporting of these processes should be enforced to enhance scientific rigor and validation.

Sixth, emphasize model explainability and ethical AI practices. Transparent and interpretable AI models are crucial for clinical adoption. Few studies currently apply advanced explainability methods such as SHAP, Local Interpretable Model-Agnostic Explanations, or counterfactual analyses. Integrating these interpretability techniques into AI pipelines will facilitate clinician trust and understanding. Moreover, ethical considerations—such as minimizing algorithmic bias (such as balanced datasets and resampling to correct imbalances in training data)—are considered in few studies, but preventing potential harms from false positives, safeguarding patient autonomy, and ensuring cultural sensitivity should be systematically addressed in all AI model developments.

Seventh, standardize evaluation and reporting metrics. While accuracy is often prioritized, metrics such as specificity, AUC, *F*_1_-score, and precision must be consistently reported to enable comprehensive evaluation and meaningful comparisons across studies. Furthermore, systematic reviews and meta-analyses are required to identify existing methodological inconsistencies, biases, and underrepresented findings to refine future AI-based research approaches.

Eighth, shift from subjective screening tools to objective validation measures. Current studies heavily rely on subjective instruments (eg, EPDS, PHQ, and *ICD* codes), which, despite validation, vary significantly across studies. Future research should validate AI models using objective clinical measures such as physiological indicators and behavioral markers, thus improving reliability and facilitating clinical implementation.

Ninth, the superior performance of ensemble models—particularly boosting techniques such as CatBoost, LightGBM, and XGBoost—suggests that they are promising candidates for clinical implementation in PPD screening. Their ability to consistently achieve high accuracy, *F*_1_-score, and AUC underscores their robustness in handling structured health data, including demographic and clinical features. Given the strong results of CatBoost in handling categorical variables and LightGBM’s efficiency in large-scale settings, future research should prioritize evaluating these models in real-world clinical workflows and mobile health platforms, where scalability and interpretability are critical. In addition, since performance varied across studies due to differences in data characteristics and preprocessing strategies, future work should aim to establish benchmark datasets and standardized pipelines for fair comparison.

Efforts should also be made to assess model performance across different population subgroups, ensuring that these algorithms do not inadvertently introduce or amplify bias. Finally, comparative studies should continue to assess whether boosting models maintain their advantage as datasets grow and diversify, particularly in longitudinal or multisite contexts.

Finally, foster global and interdisciplinary collaboration. PPD research remains unevenly distributed globally. Encouraging cross-regional and interdisciplinary collaboration—particularly with underrepresented regions and diverse professional backgrounds such as computer science, psychiatry, public health, and ethics—will foster equitable research practices and drive innovation in maternal mental health care globally.

### Limitations

Despite the comprehensive nature of this scoping review, several limitations should be acknowledged; first, we limited our inclusion to studies published in English. This language restriction may have resulted in the exclusion of relevant research published in other languages, particularly from non–English-speaking countries where maternal mental health may be a pressing issue.

Second, we prioritized peer-reviewed and indexed literature from 8 major databases and limited Google Scholar results to the first 100 entries ranked by relevance. Consequently, gray literature, including government reports, dissertations beyond ProQuest, and nonindexed conference proceedings, may have been underrepresented.

Third, our review focused specifically on studies using AI techniques for detection or prediction of PPD. As a result, studies that applied AI for monitoring, treatment delivery, or resource allocation in maternal mental health were excluded, which narrows the scope of applicability.

Finally, we did not conduct a quantitative meta-analysis or risk of bias assessment, as these are typically outside the scope of scoping reviews. Consequently, while we mapped methodological patterns and gaps, we did not evaluate effect sizes, statistical heterogeneity, or study-level quality in a standardized manner.

### Conclusions

This scoping review comprehensively maps the application of AI in PPD research, analyzing 87 studies published between 2009 and 2025. The review identifies a predominant emphasis on early prediction (∼80%) over detection, with ML methods—particularly RF (44.6%), LogR (35.4%), and XGBoost (23.1%)—used in 87.7% of studies. These models were favored for their compatibility with structured clinical data and interpretability. DL approaches, including CNNs and RNNs, were underutilized (16.9%), reflecting data limitations and interpretability concerns. NLP and RL were rarely applied, mirroring limited access to unstructured or sequential data sources.

More than 90% of studies focused on classification tasks, aligning with standard clinical workflows, while regression approaches remained limited. Basic preprocessing practices, such as normalization, were widely adopted (78.5%), but advanced strategies—such as imputation (44.6%), resampling (23.1%), cost-sensitive learning (6.2%), and feature selection techniques such as PCA or SHAP—were inconsistently applied. Most models lacked detailed reporting of optimization strategies or regularization methods, and only half used internal validation. External validation was rarely reported, complicating model comparability.

The comparative analysis between accuracy, *F*_1_-score, and AUC confirms that ensemble learning approaches, particularly boosting algorithms such as CatBoost and LightGBM, consistently outperform traditional models in predicting PPD, achieving superior accuracy, *F*_1_-scores, and AUC values across studies.

Geographic trends showed research dominance by North America, particularly the United States, with notable contributions from Asia, driven by access to public datasets. Most studies used retrospective designs and unimodal inputs—mainly survey or EHR data—while multimodal and objective data (eg, biomarkers and sensor data) were rarely incorporated. Assessment timing, feature selection, and dataset transparency varied widely. Sociodemographic and obstetric features were the most frequently used predictors, while linguistic, behavioral, and physiological data were underrepresented. This review offers the first detailed synthesis of preprocessing workflows and feature domains in PPD-AI research, underscoring both progress and methodological gaps across the literature.

## Supplementary material

10.2196/77376 Multimedia Appendix 1 Search strategy.

10.2196/77376 Multimedia Appendix 2 Data extraction form.

10.2196/77376 Multimedia Appendix 3 Characteristics of each included study.

10.2196/77376 Multimedia Appendix 4 Characteristics of the dataset.

10.2196/77376 Multimedia Appendix 5 Characteristics of preprocessing.

10.2196/77376 Multimedia Appendix 6 Feature characteristics.

10.2196/77376 Multimedia Appendix 7 Characteristics of artificial intelligence.

10.2196/77376 Multimedia Appendix 8 Characteristics of machine learning performances.

10.2196/77376 Multimedia Appendix 9 Prompts and responses.

10.2196/77376 Checklist 1 PRISMA-ScR (Preferred Reporting Items for Systematic Reviews and Meta-Analyses Extension for Scoping Reviews) checklist.

## Abbreviations

AI: artificial intelligence
AUC: area under the curve
CNN: convolutional neural network
DL: deep learning
EHR: electronic health record
EPDS: Edinburgh Postnatal Depression Scale
*ICD*: *International Classification of Diseases*
LIWC: linguistic inquiry and word count
LogR: logistic regression
ML: machine learning
PCA: principal component analysis
PHQ-9: Patient Health Questionnaire-9
PPD: postpartum depression
PRISMA-ScR: Preferred Reporting Items for Systematic Reviews and Meta-Analyses Extension for Scoping Reviews
RF: random forest
RL: reinforcement learning
RNN: recurrent neural network
