# Supplementary material for: AI for Detecting and Predicting Postpartum Depression: Scoping Review
Source: J Med Internet Res. 2026 Jan 8;28:e77376. doi: 10.2196/77376 (PMC12782538; doi:10.2196/77376)
Supplement: Multimedia Appendix 1 [file jmir-v28-e77376-s001.docx]

**Multimedia Appendix 1.** Search strategy.

Database(s): **Ovid MEDLINE(R) ALL**1946 to November 15, 2024
Search Strategy:

| **#** | **Searches** | **Results** |
| --- | --- | --- |
| 1 | exp Depression, Postpartum/ | 8184 |
| 2 | "Postpartum depression".tw. | 6026 |
| 3 | "Post-partum depression".tw. | 334 |
| 4 | "Postnatal depression".tw. | 5232 |
| 5 | "Post-natal Depression".tw. | 167 |
| 6 | "postpartum blues".tw. | 136 |
| 7 | "Post-delivery depression".tw. | 3 |
| 8 | "Baby blues".tw. | 99 |
| 9 | "Maternal depression".tw. | 3452 |
| 10 | exp Artificial Intelligence/ | 214578 |
| 11 | "Artificial Intelligence".tw. | 51304 |
| 12 | "Machine Learning".tw. | 113577 |
| 13 | "Deep Learning".tw. | 63908 |
| 14 | "Generative Pre-trained Transformer".tw. | 493 |
| 15 | "Generative Pre trained Transformer*".tw. | 538 |
| 16 | "Generative AI".tw. | 666 |
| 17 | "Large language model*".tw. | 3124 |
| 18 | "pre-trained transformer".tw. | 556 |
| 19 | "Decision Tree*".tw. | 17636 |
| 20 | "K-Nearest Neighbor*".tw. | 6380 |
| 21 | "Support vector machine*".tw. | 29796 |
| 22 | "Recurrent Neural Network*".tw. | 4876 |
| 23 | "Convolutional Neural Network*".tw. | 29733 |
| 24 | "Artificial neural network*".tw. | 19557 |
| 25 | "Deep Neural Network*".tw. | 11394 |
| 26 | "Naïve Bayes".tw. | 9 |
| 27 | "Naive Bayes".tw. | 3880 |
| 28 | "Bayesian Networks".tw. | 1651 |
| 29 | "Fuzzy Logic".tw. | 2662 |
| 30 | "K-Means".tw. | 8431 |
| 31 | "Random Forest*".tw. | 30094 |
| 32 | "Long Short-Term Memory*".tw. | 5919 |
| 33 | "Gradient Boost*".tw. | 8443 |
| 34 | AdaBoost.tw. | 1759 |
| 35 | "Multilayer Perceptron".tw. | 3280 |
| 36 | "Ensemble learning".tw. | 2395 |
| 37 | "Generative Adversarial Network*".tw. | 3991 |
| 38 | "Transfer Learning".tw. | 6449 |
| 39 | 1 or 2 or 3 or 4 or 5 or 6 or 7 or 8 or 9 | 15101 |
| 40 | 10 or 11 or 12 or 13 or 14 or 15 or 16 or 17 or 18 or 19 or 20 or 21 or 22 or 23 or 24 or 25 or 26 or 27 or 28 or 29 or 30 or 31 or 32 or 33 or 34 or 35 or 36 or 37 or 38 | 384558 |
| 41 | 39 and 40 | 89 |
| 42 | limit 41 to (english language ^a^ and humans ^b^) | 64 |
| ^a^ "Studies not published in English were excluded from this review."  ^b^ *"This review is limited to studies involving human female participants; animal studies were excluded."* | | |

Database(s): **Embase**1974 to 2024 Week 46
Search Strategy:

| **#** | **Searches** | **Results** |
| --- | --- | --- |
| 1 | exp Depression, Postpartum/ | 8917 |
| 2 | "Postpartum depression".tw. | 7917 |
| 3 | "Post-partum depression".tw. | 575 |
| 4 | "Postnatal depression".tw. | 6857 |
| 5 | "Post-natal Depression".tw. | 264 |
| 6 | "postpartum blues".tw. | 211 |
| 7 | "Post-delivery depression".tw. | 4 |
| 8 | "Baby blues".tw. | 137 |
| 9 | "Maternal depression".tw. | 4346 |
| 10 | exp Artificial Intelligence/ | 119916 |
| 11 | "Artificial Intelligence".tw. | 60183 |
| 12 | "Machine Learning".tw. | 132466 |
| 13 | "Deep Learning".tw. | 73371 |
| 14 | "Generative Pre-trained Transformer".tw. | 449 |
| 15 | "Generative Pre trained Transformer*".tw. | 488 |
| 16 | "Generative AI".tw. | 667 |
| 17 | "Large language model*".tw. | 3270 |
| 18 | "pre-trained transformer".tw. | 515 |
| 19 | "Decision Tree*".tw. | 24187 |
| 20 | "K-Nearest Neighbor*".tw. | 7353 |
| 21 | "Support vector machine*".tw. | 35134 |
| 22 | "Recurrent Neural Network*".tw. | 5468 |
| 23 | "Convolutional Neural Network*".tw. | 34499 |
| 24 | "Artificial neural network*".tw. | 22363 |
| 25 | "Deep Neural Network*".tw. | 12647 |
| 26 | "Naïve Bayes".tw. | 25 |
| 27 | "Naive Bayes".tw. | 4719 |
| 28 | "Bayesian Networks".tw. | 1891 |
| 29 | "Fuzzy Logic".tw. | 3136 |
| 30 | "K-Means".tw. | 11123 |
| 31 | "Random Forest*".tw. | 36208 |
| 32 | "Long Short-Term Memory*".tw. | 5892 |
| 33 | "Gradient Boost*".tw. | 9890 |
| 34 | AdaBoost.tw. | 2071 |
| 35 | "Multilayer Perceptron".tw. | 3590 |
| 36 | "Ensemble learning".tw. | 2626 |
| 37 | "Generative Adversarial Network*".tw. | 4446 |
| 38 | "Transfer Learning".tw. | 6921 |
| 39 | 1 or 2 or 3 or 4 or 5 or 6 or 7 or 8 or 9 | 19506 |
| 40 | 10 or 11 or 12 or 13 or 14 or 15 or 16 or 17 or 18 or 19 or 20 or 21 or 22 or 23 or 24 or 25 or 26 or 27 or 28 or 29 or 30 or 31 or 32 or 33 or 34 or 35 or 36 or 37 or 38 | 395528 |
| 41 | 39 and 40 | 134 |
| 42 | limit 41 to (english language ^a^and humans^b^) | 126 |
| 43 | limit 42 to "remove medline records ^c^" | 48 |
| ^a^ "Studies not published in English were excluded from this review."  ^b^ *"This review is limited to studies involving human female participants; animal studies were excluded."*  *^c^ "Removing MEDLINE records helps avoid duplication of studies already retrieved from previous databases.”* | | |

| Database | Query | Hits |
| --- | --- | --- |
| PsycInfo | AB ( "Postpartum depression" OR "Post-partum depression" OR "Postnatal depression" OR "Post-natal Depression" OR "postpartum blues" OR "Post-delivery depression" OR "Baby blues" OR "Maternal depression" ) AND AB ( "Artificial Intelligence" OR "Machine Learning" OR "Deep Learning" OR "Generative Pre-trained Transformer" OR "Generative Pre trained Transformer*" OR "Generative AI" OR "Large language model*" OR "pre-trained transformer" OR "Decision Tree*" OR "K-Nearest Neighbor*" OR "Support vector machine*" OR "Recurrent Neural Network*" OR "Convolutional Neural Network*" OR "Artificial neural network*" OR "Deep Neural Network*" OR "Naïve Bayes" OR "Naive Bayes" OR "Bayesian Networks" OR "Fuzzy Logic" OR "K-Means" OR "Random Forest*" OR "Long Short-Term Memory*" OR "Gradient Boost*" OR AdaBoost OR "Multilayer Perceptron" OR "Ensemble learning" OR "Generative Adversarial Network*" OR "Transfer Learning" ) **Narrow by Language:**- english | 26 |
| CINAHL | AB ( "Postpartum depression" OR "Post-partum depression" OR "Postnatal depression" OR "Post-natal Depression" OR "postpartum blues" OR "Post-delivery depression" OR "Baby blues" OR "Maternal depression" ) AND AB ( "Artificial Intelligence" OR "Machine Learning" OR "Deep Learning" OR "Generative Pre-trained Transformer" OR "Generative Pre trained Transformer*" OR "Generative AI" OR "Large language model*" OR "pre-trained transformer" OR "Decision Tree*" OR "K-Nearest Neighbor*" OR "Support vector machine*" OR "Recurrent Neural Network*" OR "Convolutional Neural Network*" OR "Artificial neural network*" OR "Deep Neural Network*" OR "Naïve Bayes" OR "Naive Bayes" OR "Bayesian Networks" OR "Fuzzy Logic" OR "K-Means" OR "Random Forest*" OR "Long Short-Term Memory*" OR "Gradient Boost*" OR AdaBoost OR "Multilayer Perceptron" OR "Ensemble learning" OR "Generative Adversarial Network*" OR "Transfer Learning" ) **Narrow by Language:**- english | 22 |
| IEEE Xplore | ("Abstract":"Postpartum depression" OR "Abstract":"Post-partum depression" OR "Abstract":"Postnatal depression" OR "Abstract":"Post-natal Depression" OR "Abstract":"postpartum blues" OR "Abstract":"Post-delivery depression" OR "Abstract":"Baby blues" OR "Abstract":"Maternal depression") AND ("Abstract":"Artificial Intelligence" OR "Abstract":"Machine Learning" OR "Abstract":"Deep Learning" OR "Abstract":"Generative Pre-trained Transformer" OR "Abstract":"Generative Pre trained Transformer*" OR "Abstract":"Generative AI" OR "Abstract":"Large language model*" OR "Abstract":"pre-trained transformer" OR "Abstract":"Decision Tree*" OR "Abstract":"K-Nearest Neighbor*" OR "Abstract":"Support vector machine*" OR "Abstract":"Recurrent Neural Network*" OR "Abstract":"Convolutional Neural Network*" OR "Abstract":"Artificial neural network*" OR "Abstract":"Deep Neural Network*" OR "Abstract":"Naïve Bayes" OR "Abstract":"Naive Bayes" OR "Abstract":"Bayesian Networks" OR "Abstract":"Fuzzy Logic" OR "Abstract":"K-Means" OR "Abstract":"Random Forest*" OR "Abstract":"Long Short-Term Memory" OR "Abstract":"Gradient Boost" OR "Abstract":"Gradient Boosting" OR "Abstract":AdaBoost OR "Abstract":"Multilayer Perceptron" OR "Abstract":"Ensemble learning" OR "Abstract":"Generative Adversarial Network" OR "Abstract":"Generative Adversarial Networks" OR "Abstract":"Transfer Learning") | 16 |
| ACM digital library | [[Abstract: "postpartum depression"] OR [Abstract: "post-partum depression"] OR [Abstract: "postnatal depression"] OR [Abstract: "post-natal depression"] OR [Abstract: "postpartum blues"] OR [Abstract: "post-delivery depression"] OR [Abstract: "baby blues"] OR [Abstract: "maternal depression"]] AND [[Abstract: "artificial intelligence"] OR [Abstract: "machine learning"] OR [Abstract: "deep learning"] OR [Abstract: "generative pre-trained transformer"] OR [Abstract: "generative pre trained transformer*"] OR [Abstract: "generative ai"] OR [Abstract: "large language model*"] OR [Abstract: "pre-trained transformer"] OR [Abstract: "decision tree*"] OR [Abstract: "k-nearest neighbor*"] OR [Abstract: "support vector machine*"] OR [Abstract: "recurrent neural network*"] OR [Abstract: "convolutional neural network*"] OR [Abstract: "artificial neural network*"] OR [Abstract: "deep neural network*"] OR [Abstract: "naïve bayes"] OR [Abstract: "naive bayes"] OR [Abstract: "bayesian networks"] OR [Abstract: "fuzzy logic"] OR [Abstract: "k-means"] OR [Abstract: "random forest*"] OR [Abstract: "long short-term memory"] OR [Abstract: "gradient boost"] OR [Abstract: "gradient boosting"] OR [Abstract: adaboost] OR [Abstract: "multilayer perceptron"] OR [Abstract: "ensemble learning"] OR [Abstract: "generative adversarial network"] OR [Abstract: "generative adversarial networks"] OR [Abstract: "transfer learning"]] | 2 |
| Scopus | ( TITLE-ABS-KEY ( "Postpartum depression" OR "Post-partum depression" OR "Postnatal depression" OR "Post-natal Depression" OR "postpartum blues" OR "Post-delivery depression" OR "Baby blues" OR "Maternal depression" ) AND TITLE-ABS-KEY ( "Artificial Intelligence" OR "Machine Learning" OR "Deep Learning" OR "Generative Pre-trained Transformer" OR "Generative Pre trained Transformer*" OR "Generative AI" OR "Large language model*" OR "pre-trained transformer" OR "Decision Tree*" OR "K-Nearest Neighbor*" OR "Support vector machine*" OR "Recurrent Neural Network*" OR "Convolutional Neural Network*" OR "Artificial neural network*" OR "Deep Neural Network*" OR "Naïve Bayes" OR "Naive Bayes" OR "Bayesian Networks" OR "Fuzzy Logic" OR "K-Means" OR "Random Forest*" OR "Long Short-Term Memory*" OR "Gradient Boost*" OR adaboost OR "Multilayer Perceptron" OR "Ensemble learning" OR "Generative Adversarial Network*" OR "Transfer Learning" ) ) AND ( EXCLUDE ( DOCTYPE , "re" ) OR EXCLUDE ( DOCTYPE , "ch" ) OR EXCLUDE ( DOCTYPE , "tb" ) OR EXCLUDE ( DOCTYPE , "ed" ) OR EXCLUDE ( DOCTYPE , "sh" ) OR EXCLUDE ( DOCTYPE , "no" ) OR EXCLUDE ( DOCTYPE , "le" ) OR EXCLUDE ( DOCTYPE , "cr" ) ) AND ( LIMIT-TO ( LANGUAGE , "English" ) ) AND ( EXCLUDE ( SRCTYPE , "k" ) ) | 145 |
| Web of Science | "Postpartum depression" OR "Post-partum depression" OR "Postnatal depression" OR "Post-natal Depression" OR "postpartum blues" OR "Post-delivery depression" OR "Baby blues" OR "Maternal depression" (Abstract) and "Artificial Intelligence" OR "Machine Learning" OR "Deep Learning" OR "Generative Pre-trained Transformer" OR "Generative Pre trained Transformer*" OR "Generative AI" OR "Large language model*" OR "pre-trained transformer" OR "Decision Tree*" OR "K-Nearest Neighbor*" OR "Support vector machine*" OR "Recurrent Neural Network*" OR "Convolutional Neural Network*" OR "Artificial neural network*" OR "Deep Neural Network*" OR "Naïve Bayes" OR "Naive Bayes" OR "Bayesian Networks" OR "Fuzzy Logic" OR "K-Means" OR "Random Forest*" OR "Long Short-Term Memory*" OR "Gradient Boost*" OR AdaBoost OR "Multilayer Perceptron" OR "Ensemble learning" OR "Generative Adversarial Network*" OR "Transfer Learning". (Abstract) and Article or Proceeding Paper (Document Types) and English (Languages) | 80 |
| Google Scholar | ("Postpartum depression" OR "Post-partum depression" OR "Postnatal depression" OR "Post-natal Depression" OR "postpartum blues" OR "Post-delivery depression" OR "Baby blues" OR "Maternal depression") AND ("Artificial Intelligence" OR "Machine Learning" OR "Deep Learning" OR "Generative Pre-trained Transformer" OR "Generative Pre trained Transformer*" OR "Generative AI" OR "Large language model*" OR "pre-trained transformer" OR "Decision Tree*" OR "K-Nearest Neighbor*" OR "Support vector machine*" OR "Recurrent Neural Network*" OR "Convolutional Neural Network*" OR "Artificial neural network*" OR "Deep Neural Network*" OR "Naïve Bayes" OR "Naive Bayes" OR "Bayesian Networks" OR "Fuzzy Logic" OR "K-Means" OR "Random Forest*" OR "Long Short-Term Memory" OR "Gradient Boost*" OR AdaBoost OR "Multilayer Perceptron" OR "Ensemble learning" OR "Generative Adversarial Network*" OR "Transfer Learning") | 100 |
