# Supplementary material for: AI for Detecting and Predicting Postpartum Depression: Scoping Review
Source: J Med Internet Res. 2026 Jan 8;28:e77376. doi: 10.2196/77376 (PMC12782538; doi:10.2196/77376)
Supplement: Multimedia Appendix 2 [file jmir-v28-e77376-s002.docx]

| **Extracted Data** | **Definition** |
| --- | --- |
| **Study Design and Population** |  |
| Author | The surname of the first author of the study. |
| Year of Publication | The year in which the study was published. |
| Publication Type | The type of publication: journal article, conference paper, dissertation. |
| Country of Publication | The country where the study was conducted or published. |
| Research Design | What are research designs used in the study: Prospective or Retrospective? |
| **Population Characteristics** |  |
| Sample Size | Total number of participants included in the study. |
| Mean Age | The average age of the participants. |
| Age Range | The age range of participants included in the study. |
| Setting | The setting from which the participants were recruited (e.g., healthcare, academic, or community settings). |
| **Data Characteristics** |  |
| Dataset Size | Total size of the dataset (e.g., number of records, samples, or observations). |
| Data Source | What is the source of the data: open or closed? |
| Data Type | Types of data used:(obstetric, medical history data, sociodemographic data, psychological data, behavioral data, sensor-based data, linguistic data,biomarker data,neonatal data) |
| Data format | The format: textual,tabular, image, audio, video. |
| Outcome measurement timing | when the depression status was assessed postpartum (in weeks) |
| Reference Standard | How the actual status (postpartum depression) of the participants was identified (e.g., EPDS, PHQ-9, ICD-10, PPDS, PDSS, PBQ, PSQI). |
| **Preprocessing Techniques** |  |
| Missing Data Handling | Methods used to address missing or incomplete data: ( Imputation, Excluded ) |
| Feature Transformation (e.g., Normalization, scaling, log-transform, Standarzation , Text cleaning , Toknezation, Data Cleaning , Augmenatation) | Techniques used to transform or modify the original features of the data (e.g., scaling (Normalization, Standardization), Power Transformations (Log Transformation), Tokenization, Discretization (Binning) |
| Unbalanced data | Techniques used to address class imbalance (e.g., oversampling, undersampling, SMOTE) |
| Feature Creation/ extraction ( e.g., Polynomial features, Interaction terms, Dimension reducation PCA, t-SNE, UMAP) | Techniques used to transform raw data into meaningful features: (e.g., Polynomial features, Interaction terms, Dimension reduction PCA, t-SNE, UMAP). |
| Feature Encoding | Encoding categorical variables (e.g., one-hot encoding, label encoding, target encoding, binary encoding). |
| Feature Selection | Techniques used to choose the most relevant and informative features (variables) from the dataset( e.g., Correlation filtering, Recursive Feature Elimination, LASSO, Tree-based importance). |
| **Features characteristics** |  |
| Number of Features | Total number of features used in the model (e.g., extracted attributes from datasets). |
| Data collection methodology | The methodology used to collect the data ( EHRs, Survey, social media,sensor-based, Laboratory-based data) |
| Features | What is the data provided to the AI models (Independent features/variables)? |
| **AI model characteristics** |  |
| Aim of AI Algorithm | Was the AI algorithm designed to predict the future occurrence of postpartum depression (Prediction) or to detect whether women currently have postpartum depression (Detection)? |
| AI Categories | What is the category of each algorithm used in the study (Machine learning, deep learning, transfer learning, reinforcement learning, etc.)? |
| Problem-Solving Approach | The type of prediction or analysis being performed by the algorithm: Regression, Classification, Clustering. |
| Algorithms Used | What are the main AI algorithms/models used in the paper to detect or predict postpartum depression (e.g., RF, SVM, ANN, CNN, RNN, DNN, k-NN, MLP, DBN, DBM, DPN BN, CRT, DT, LASSO, LR, MFA, MLR, MDL, NB, NN, NSC, RBFN)? |
| Validation Techniques | What is the approach that was used to validate the developed algorithm (e.g. Training-test split (Hold-out), K-fold cross validation, Nested Cross Validation, Leave One Out cross validation, Apparent validation, external validation)? |
| Performance Metrics | Metrics used to assess the performance of the algorithm (e.g., accuracy, sensitivity (recall), specificity, precision, AUC, etc). |
| Optimization Strategies | Methods to improve model accuracy (e.g., Gradient-Based, Learning Rates, Regularization, Loss Cross-Entropy, MSE, custom loss functions, Model Pruning, Quantization, Knowledge DistillationDistributed, Gradient Accumulation, Mixed-Precision, multi-GPU, Tuning, Grid/Random Search, Bayesian Optimization). |
