# Supplementary material for: AI for Detecting and Predicting Postpartum Depression: Scoping Review
Source: J Med Internet Res. 2026 Jan 8;28:e77376. doi: 10.2196/77376 (PMC12782538; doi:10.2196/77376)
Supplement: Multimedia Appendix 3 [file jmir-v28-e77376-s003.docx]

**Multimedia Appendix 3.** Characteristics of each included study.

| Study [References] | Author | Year of publication | Publication type | Country of publication | Research Design) | # participants | Mean Age | Age range |
| --- | --- | --- | --- | --- | --- | --- | --- | --- |
| Ajay et al [24] | Ajay | 2024 | Conference paper | United States | Retrospective | 137 | NR | NR |
| Sharma et al [21] | Sharma | 2024 | Conference paper | Bangladesh | Retrospective | 1503 | NR | 25-30 |
| Andersson et al [22] | Andersson | 2021 | Journal article | Sweden | Prospective | 4313 | 31 | 18–42 |
| Betts et al [23] | Betts | 2020 | Journal article | Australia | Retrospective | 75054 | NR | NR |
| Cai et al [25] | Cai | 2019 | Journal article | United States | Retrospective | 586 | NR | 14–42 |
| Carneiro et al [26] | Carneiro | 2021 | Conference paper | Brazil | Retrospective | 11 | NR | NR |
| Chen et al [27] | Chen | 2018 | Conference paper | China | Retrospective | 446 | NR | NR |
| Fanos et al [28] | Fanos | 2023 | Journal article | Italy | Prospective | 154 | 34.4 | 31-35 |
| Fatima et al [29] | Fatima | 2019 | Journal article | Saudi Arabia | Prospective | 3176 | NR | NR |
| Fazraningtyas et al [30] | Fazraningtyas | 2025 | Journal article | Indonesia | Retrospective | 317 | NR | NR |
| Gabrieli et al [31] | Gabrieli | 2020 | Journal article | United States | Prospective | 56 | 31.1 | NR |
| Gopalakrishnan et al [34] | Gopalakrishnan | 2022 | Journal article | India | Prospective | 217 | NR | 19-35 |
| Gopalakrishnan et al [33] | Gopalakrishnan a | 2023 | Journal article | India | Prospective | 314 | 25.99 | 19-35 |
| Gopalakrishnan et al [32] | Gopalakrishnan b | 2023 | Conference paper | India | Retrospective | 8547 | NR | 19-35 |
| Gupta et al [35] | Gupta | 2024 | Conference paper | Bangladesh | Retrospective | 1503 | NR | 25-30 |
| Horgen [36] | Horgen | 2022 | Dissertation | Norway | Prospective | 41807 | NR | NR |
| Hurwitz et al [37] | Hurwitz | 2024 | Journal article | United States | Retrospective | 59 | NR | NR |
| Jimenez-Serrano et al [38] | Jimenez-Serrano | 2015 | Journal article | Spain | Prospective | 1397 | NR | NR |
| Krishnamurti et al [39] | Krishnamurti | 2022 | Journal article | United States | Prospective | 666 | 30.3 | 18-45 |
| Lilhore et al [41] | Lilhore c | 2024 | Journal article | United States | Retrospective | 1745 | NR | NR |
| Lilhore et al [40] | Lilhore d | 2024 | Journal article | Bangladesh | Retrospective | 1503 | NR | 25-30 |
| Liu et al [43] | Liu | 2024 | Journal article | United States | Retrospective | 55303 | 32.4 | 18-45 |
| Liu et al [42] | Liu | 2023 | Journal article | China | Retrospective | 1436 | 30 | NR |
| Lyall et al [44] | Lyall | 2023 | Journal article | United Kingdom | Retrospective | 64353 | NR | 37-73 |
| Marshad et al [45] | Marshad | 2024 | Conference paper | Bangladesh | Retrospective | 1503 | NR | 25-30 |
| Matsuo et al [47] | Matsumura | 2022 | Journal article | Japan | Prospective | 84091 | 31.1 | NR |
| Matsuo et al [47] | Matsuo | 2022 | Journal article | Japan | Retrospective | 10013 | NR | NR |
| Mazumder and Baruah [48] | Mazumder and Baruah | 2021 | Conference paper | India | Retrospective | 96 | NR | 23-29 |
| Moreira et al [49] | Moreira | 2019 | Journal article | Portugal | Retrospective | 205 | NR | NR |
| Mustafa [50] | Mustafa | 2023 | Journal article | Pakistan | Prospective | 1258 | 31.78 | NR |
| Myneni et al [51] | Myneni | 2024 | Journal article | United States | Prospective, Retrospective | 9404 | NR | NR |
| Nasim et al [52] | Nasim | 2024 | Journal article | Bangladesh | Retrospective | 1503 | NR | 25-30 |
| Natarajan et al [53] | Natarajan | 2017 | Journal article |  |  |  |  |  |
| Osubor and Egwali [54] | Osubor and Egwali | 2018 | Conference paper | United States | Retrospective | 173 | NR | NR |
| Park et al [55] | Park | 2021 | Journal article | Nigeria | Retrospective | 59 | NR | NR |
| Paul et al [56] | Paul | 2023 | Journal article | United States | Retrospective | 573634 | 26.1 | 12-55 |
| Payne et al [57] | Payne | 2020 | Conference paper | India | Retrospective | 28755 | NR | NR |
| Prabhashwaree and Wagarachchi [58] | Prabhashwaree and Wagarachchi | 2022 | Journal article | United States | Prospective | 285 | NR | NR |
| Prabhashwaree and Wagarachchi [59] | Prabhashwaree and Wagarachchi | 2022 | Conference paper | Sri Lanka | Prospective | 704 | 29 | NR |
| Qasrawi et al [60] | Qasrawi | 2022 | Conference paper | Sri Lanka | Prospective | 686 | 29 | NR |
| Raisa et al [61] | Raisa | 2022 | Journal article | Palestine | Retrospective | 3569 | 28.5 | 21-40 |
| Reps et al [62] | Reps | 2022 | Conference paper | Bangladesh | Retrospective | 150 | NR | NR |
| Shen et al [63] | Shen | 2023 | Journal article | United States | Prospective | 858 | NR | NR |
| Shin et al [64] | Shin | 2020 | Journal article | China | Prospective | 4313 | 31 | 18–42 |
| Shivaprasad et al [65] | Shivaprasad | 2024 | Journal article | United States | Retrospective | 28755 | NR | NR |
| Srivatsav and Nanthini [66] | Srivatsav and Nanthini | 2024 | Journal article | Bangladesh | Retrospective | 1503 | NR | 25-30 |
| Suganthi and Geetha [67] | Suganthi and Geetha | 2024 | Conference paper | India | Retrospective | 40 | NR | NR |
| Susič et al [68] | Susič | 2023 | Journal article | Bangladesh | Prospective | 1503 | NR | 25-30 |
| Tang et al [69] | Tang | 2024 | Journal article | Slovenia | Prospective | 261 | 31.4 | NR |
| Tortajada et al [70] | Tortajada | 2009 | Journal article | China | Prospective | 500 | NR | NR |
| Valavani et al [71] | Valavani | 2020 | Journal article | Spain | Prospective | 1397 | NR | NR |
| Valdeolivar-Hernandez et al [72] | Valdeolivar-Hernandez | 2022 | Conference paper | United Kingdom | Retrospective | 144 | 32.5 | 20-40 |
| Wagay [73] | Wagay | 2023 | Conference paper | Mexico | Retrospective | 16 | NR | NR |
| Wakefield and Frasch [74] | Wakefield and Frasch | 2023 | Conference paper | Bangladesh | Retrospective | 1503 | NR | 25-30 |
| Wang et al [78] | Wang | 2024 | Journal article | United States | Retrospective | 8454 | NR | NR |
| Wang et al [76] | Wang | 2019 | Journal article | China | Prospective | 3175 | 32.46 | 21-47 |
| Wang et al [75] | Wang | 2018 | Journal article | United states | Retrospective | 9980 | 33.92 | NR |
| Wang et al [77] | Wang | 2025 | Conference paper | China | Retrospective | 740 | 44.5 | 30–60 |
| Xu et al [79] | Xu | 2023 | Journal article | China | Prospective | 121 | 31 | 20-45 |
| Xu and Sampson [80] | Xu | 2023 | Journal article | China | Prospective | 76 | 32.7 | NR |
| Yu et al [81] | Yu | 2022 | Journal article | United States | Retrospective | 2361 | 28.4 | 18-45 |
| Zhang et al [82] | Zhang | 2020 | Journal article | Japan | Prospective | 431 | 31.17 | NR |
| Zhang et al [83] | Zhang | 2024 | Journal article | China | Prospective | 508 | 28.64 | NR |
| Zhang et al [84] | Zhang | 2021 | Journal article | United states | Retrospective | 65197 | NR | 18–45 |
| Zhu et al [85] | Zhu | 2024 | Journal article | United States | Retrospective | 69169 | 30.75 | 18-45 |
| NR: Not reported |  |  |  |  |  |  |  |  |
