# Supplementary material for: AI for Detecting and Predicting Postpartum Depression: Scoping Review
Source: J Med Internet Res. 2026 Jan 8;28:e77376. doi: 10.2196/77376 (PMC12782538; doi:10.2196/77376)
Supplement: Multimedia Appendix 4 [file jmir-v28-e77376-s004.docx]

**Multimedia Appendix 4**. Characteristics of the dataset.

| Study [References] | Dataset Size | | Data sources | Data format | Data collection methodology | Setting | Outcome measurement time | Reference Standard |  |
| --- | --- | --- | --- | --- | --- | --- | --- | --- | --- |
| Ajay et al [24] | 137 | Closed | | Tabular | Survey | Academic | 48 | EPDS |  |
| Sharma et al [21] | 1503 | Open | | Tabular | Survey | Healthcare | NR | NR |  |
| Andersson et al [22] | 4313 | Closed | | Tabular | Survey | Healthcare | 48 | EPDS |  |
| Betts et al [23] | 75054 | Closed | | Tabular | EHRs | Healthcare | 48 | ICD-10 |  |
| Cai et al [25] | 586 | Closed | | Tabular | Survey | Community | NR | PDSS |  |
| Carneiro et al [26] | 297 | Closed | | Tabular | Survey | Academic | NR | EPDS |  |
| Chen et al [27] | 1170446 | Closed | | Tabular, Textual | Survey, Social media | Community | NR | EPDS |  |
| Fanos et al [28] | 154 | Open | | Audio, Tabular, Image | Survey | Healthcare | NR | EPDS |  |
| Fatima et al [29] | 3176 | Closed | | Textual | Social media | Community | NR | EPDS, ICD-10 |  |
| Fazraningtyas et al [30] | 317 | Closed | | Tabular | Survey | Healthcare | NR | EPDS |  |
| Gabrieli et al [31] | 715 | Closed | | Audio, Video | Survey | Community | NR | NR |  |
| Gopalakrishnan et al [32] | 217 | Open | | Tabular | Survey | Healthcare | 1 - 6 | EPDS, PDSS, PHQ-9 |  |
| Gopalakrishnan et al [33] | 314 | Open | | Tabular, Textual | Survey, Social Media | Healthcare | NR | NR |  |
| Gopalakrishnan et al [34] | 8547 | Closed | | Textual | Social media | Community | NR | PPDS |  |
| Gupta et al [35] | 1503 | Open | | Tabular | Survey | Healthcare | NR | NR |  |
| Horgen [36] | 41807 | Closed | | Tabular | Survey | Community | 24,72,144 | NR |  |
| Hurwitz et al [37] | 59 | Closed | | Tabular | EHRs, Sensor-based | Healthcare and academic | 96 | NR |  |
| Jimenez-Serrano et al [38] | 1397 | Closed | | Tabular | Survey, Sensor-based | Healthcare | 8, 32 | EPDS |  |
| Krishnamurti et al [39] | 1007 | Closed | | Tabular | Sensor- based | Healthcare | NR | EPDS |  |
| Lilhore et al [41] | 1745 | Open | | Audio, Tabular, Image | EHRs, Survey | Healthcare | NR | ICD-10 |  |
| Lilhore et al [40] | 1503 | Open | | Tabular | Survey | Healthcare | NR | EPDS, PHD-9 |  |
| Liu et al [43] | 55303 | Closed | | Tabular | EHRs | Healthcare | 48 | PHQ-9, PHQ-3 |  |
| Liu et al[42] | 1436 | Closed | | Tabular | EHRs, Survey | Healthcare | 6 | EPDS, ICD_9 , ICD_10 |  |
| Lyall et al [44] | 64353 | Closed | | Tabular | EHRs, Sensor-based, Survey | Academic | NR | ICD-10 |  |
| Marshad et al [45] | 1503 | Open | | Tabular | Survey | Healthcare | NR | NR |  |
| Matsumura et al [46] | 84091 | Closed | | Tabular | EHRs, Survey | Healthcare | 4, 24 | EPDS |  |
| Matsuo et al [47] | 10013 | Closed | | Tabular | EHRs | Healthcare | 4 | EPDS |  |
| Mazumder and Baruah [48] | 96 | Closed | | Tabular | Survey | Community | 48 | PDSS |  |
| Moreira et al [49] | 205 | Closed | | Tabular | EHRs,Sensor-based | Healthcare | NR | ICD-10 |  |
| Mustafa [50] | 1258 | Closed | | Tabular | EHRs, Survey | Healthcare | 8 , 32 | EPDS |  |
| Myneni et al [51] | 55341 | Closed | | Tabular, Textual, Image | Survey, Social media | Healthcare & Community | NR | NR |  |
| Nasim et al [52] | 1503 | Open | | Tabular | Survey | Healthcare | 4 | EPDS |  |
| Natarajan et al [53] | 173 | Closed | | Tabular, Textual | Survey, Social media | Community | 48 | EPDS |  |
| Osubor and Egwali [54] | 59 | Closed | | Tabular | Survey | Healthcare | NR | NR |  |
| Park et al [55] | 573634 | Closed | | Tabular | EHRs | Healthcare | 8.5 | EPDS |  |
| Paul et al [56] | 28755 | Open | | Tabular | Survey | Community | NR | EPDS |  |
| Payne et al [57] | 285 | Closed | | Tabular | Survey | Healthcare | 2, 6, 12, 20, and 36 | EPDS |  |
| Prabhashwaree and Wagarachchi [58] | 704 | Closed | | Tabular | Survey | Healthcare | 24 | EPDS |  |
| Prabhashwaree and Wagarachchi [59] | 686 | Closed | | Tabular | Survey | Healthcare | 24 | EPDS |  |
| Qasrawi et al [60] | 3569 | Closed | | Tabular | Survey | Community | NR | PHQ-9, Generalized Anxiety Disorder scale (GAD-7) |  |
| Raisa et al [61] | 150 | Closed | | Tabular | Survey | Community | NR | EPDS, PHQ-2 |  |
| Reps et al [62] | 858 | Closed | | Tabular | Survey | Community | 4, 12 | EPDS, Generalized Anxiety Disorder scale (GAD-7) |  |
| Shen et al [63] | 4313 | Closed | | Tabular | Survey | Healthcare | 6 , 24 and 48 | EPDS |  |
| Shin et al [64] | 28755 | Open | | Tabular | Survey | Community | 48 | PHQ-2 |  |
| Shivaprasad et al [65] | 1503 | Open | | Tabular | Survey | Healthcare | NR | NR |  |
| Srivatsav and Nanthini [66] | 140 | Open | | Textual | Social media | Academic | NR | NR |  |
| Suganthi and Geetha [67] | 1503 | Open | | Tabular, Textual | EHRs, Survey, Social media | Healthcare & Community | 6 | EPDS, PPDS, PHQ-9, |  |
| Susič et al [68] | 261 | Closed | | Tabular | EHRs, Survey | Healthcare | 6 | EPDS |  |
| Tang et al [69] | 4313 | Closed | | Tabular | EHRs, Survey | Healthcare | 52 | EPDS |  |
| Tortajada et al [70] | 1397 | Closed | | Tabular | EHRs, Survey, | Healthcare | 32 | EPDS , DIGS |  |
| Valavani et al [71] | 144 | Closed | | Tabular | Survey | Healthcare | 4 | EPDS |  |
| Valdeolivar-Hernandez et al [72] | 16 | Closed | | Tabular | Survey | Community | 12 - 28 | EPDS |  |
| Wagay [73] | 1503 | Open | | Tabular | EHRs, Survey | Healthcare | NR | NR |  |
| Wakefield and Frasch [74] | 10038 | Closed | | Tabular | EHRs | Healthcare | 4 | NR |  |
| Wang et al [78] | 3175 | Closed | | Tabular | EHRs, Survey | Healthcare | 4, 12, 24 | EPDS |  |
| Wang et al [76] | 9980 | Closed | | Tabular | EHRs | Healthcare | 48 | EPDS, ICD-9-CM, ICD-10-CM |  |
| Wang et al [75] | 21459 | Closed | | Audio | Survey | Healthcare | NR | NR |  |
| Wang et al [77] | 1500 | Closed | | Tabular | EHRs | Healthcare | 6 | EPDS |  |
| Xu et al [79] | 76 | Closed | | Tabular | EHRs, Survey, Laboratory-based | Healthcare | NR | NR |  |
| Xu and Sampson [80] | 2361 | Open | | Tabular | Survey | Community | 24 - 44 | EPDS |  |
| Yu et al [81] | 431 | Closed | | Tabular | EHRs, Survey, Laboratory-based | Community | 4 - 5 | EPDS |  |
| Zhang et al [82] | 508 | Closed | | Tabular | Survey | Healthcare | 6 | EPDS |  |
| Zhang et al [83] | 65197 | Closed | | Tabular | EHRs | Healthcare | 48 | ICD-10 |  |
| Zhang et al [84] | 69169 | Closed | | Tabular | EHRs | Healthcare | 48 | NR |  |
| Zhu et al [85] | 485 | Closed | | Tabular | EHRs, Survey | Healthcare | 1, 24, 48 | NR |  |
| NR: Not reported |  |  | |  |  |  |  |  |  |
