# Supplementary material for: AI for Detecting and Predicting Postpartum Depression: Scoping Review
Source: J Med Internet Res. 2026 Jan 8;28:e77376. doi: 10.2196/77376 (PMC12782538; doi:10.2196/77376)
Supplement: Multimedia Appendix 5 [file jmir-v28-e77376-s005.docx]

**Multimedia Appendix 5.** Characteristics of preprocessing.

| Study [References] | Missing Data Handling | Feature Transformation | | Unbalanced data | Feature Creation/ extraction | Feature Encoding | Feature selection |
| --- | --- | --- | --- | --- | --- | --- | --- |
| Ajay et al [24] | Imputation (mean) | Scaling, Standardization | | NR | NR | Label encoding | CoxPHFitter model and Kaplan-Meier curves for survival analysis |
| Sharma et al [21] | NR | NR | | NR | NR | NR | Chi-Square Test |
| Andersson et al [22] | Imputation (mean) | Normalization | | NR | NR | Binary encoding | Tree-based importance |
| Betts et al [23] | Excluded outliers | Standardization | | Resampling parallelized | NR | Label encoding | Tree-based importance, L1/L2 Regularization |
| Cai et al [25] | Attributes Filters | Pruning | | NR | NR | Label encoding | Tree-based importance, Information gain ratio (entropy) |
| Carneiro et al [26] | NR | Parsing and text classification, Tokenization, stopword, steming | | NR | NR | one-hot encoding | NR |
| Chen et al [27] | Excluded outliers | Tokenization | | NR | NR | one-hot encoding | NR |
| Fanos et al [28] | Excluded outliers | NR | | NR | NR | NR | L1/L2 Regularization |
| Fatima et al [29] | Excluded outliers | Normalization, text cleaning | | Resampling (SMOTE) | LIWC-generated features | Label encoding | Lasso ( Non zero coefficients) |
| Fazraningtyas et al [30] | NR | Standardization | | NR | NR | NR | Recursive Feature Elimination, Information gain ratio (entropy) |
| Gabrieli et al [31] | Imputation (balanced two-way tables) | Scalling, Data Augmentation | | Resampling ( White Gaussian Noise (AWGN)) | Acoustic feature extraction | Label encoding | NR |
| Gopalakrishnan et al [34] | Excluded, Imputation (MICE, KNN) | Standardization, attribute distributions, elimination | | NR | NS | Binary encoding | Gini Index or Mean Decrease in Impurity (MDI) |
| Gopalakrishnan et al [33] | Imputation | Stemming, Lemmatization, Tokenization, Stop data removal (elimination) | | NR | N-gram characteristics, LDA topics, t-SNE | Label encoding | Pearson correlation |
| Gopalakrishnan b et al. [32] | NR | Normalization, Scaling, text cleaning, Tokenization | | NR | NR | one-hot encoding | NR |
| Gupta et al [35] | NR | NR | | NR | NR | NR | NR |
| Horgen [36] | Excluded, Imputation (MICE, KNN) | Normalization, Standardization, text cleaning, Scaling | | Former undersampling & Latter as oversampling | Principal Component Analysis (PCA) | one-hot encoding | Correlation analysis, L1/L2 Regularization |
| Hurwitz et al [37] | Excluded outliers | Normalization, Stemming | | NR | NR | NR | SHAP values (Differential Evolution) |
| Jimenez-Serrano et al [38] | Imputation (Mode), Excluded | Standardization | | NR | NR | Label encoding | NR |
| Krishnamurti et al [39] | NR | NR | | NR | NR | NR | NR |
| Lilhore et al [41] | Imputation | Normalization, Standardization, text cleaning, Scaling | | NR | NR | one-hot encoding, Target Encoding | "Correlation analysis, Gini Index or Mean Decrease in Impurity (MDI), |
| Lilhore et al [40] | Elimnation, Excluded outliers | Normalization, Tokenization, Augmentation, emmbeding, Stop data, Interaction Terms | | NR | NR | Binary encoding | information gain (entropy)" |
| Liu et al [43] | Imputation (KNN) | Normalization, Interaction Terms | | NR | NR | one-hot encoding | Recursive Feature Elimination, Tree-based Importance |
| Liu et al [42] | NR | NR | | NR | NR | Label encoding | SHAP values (Differential Evolution) |
| Lyall et al [44] | Imputation | NR | | NR | NR | NR | L1/L2 Regularization |
| Marshad et al [45] | Excluded outliers | EDA | | NR | NR | NR | Correlation analysis, L1/L2 Regularization |
| Matsumura et al [46] | Imputation (Mode) | HPSPLIT, Prunning | | NR | NR | NR | Correlation analysis |
| Matsuo et al [47] | Excluded outliers | text cleaning | | Resampling (SMOTE) | NR | Dummy encoding | Tree-based importance, Information gain ratio (entropy) |
| Mazumder and Baruah [48] | NR | NR | | NR | NR | Label encoding | NR |
| Moreira et al [49] | NR | Normalization, Standardization | | NR | NR | NR | Correlation analysis |
| Mustafa [50] | Excluded outliers | Normalization, Standardization, text cleaning | | Resampling | NR | Label encoding, Binary encoding | NR |
| Myneni et al [51] | Excluded, Imputation | Normalization, text cleaning, Tokenization | | cascading classification and fine-tuned BERT models | Domain-Specific Analysis, Dimension Reduction | Label encoding | NR |
| Nasim et al [52] | Imputation | Normalization, Standardization, text cleaning | | Resampling (SMOTE) | Principal Component Analysis (PCA) | One-hot encoding | NR |
| Natarajan et al [53] | Imputation (gradient methods) | NR | | Resampling (SMOTE) | NR | Label encoding | Tree-based importance |
| Osubor and Egwali [54] | Excluded, Imputation | layer normalization | | NR | NR | NR | NR |
| Park et al [55] |  |  | | Reweighing | NR | NR | Correlation analysis |
| Paul et al [56] | Implied by Sparse Array Conversion | Normalization, Scaling | | Oversampling (Synthetic Minority ), Resampling (SMOTE) | Domain-Specific Analysis, Dimension Reduction | one-hot encoding | NR |
| Payne et al [57] | NR | Statistical analysis (non parametric test) | | NR | Linear Discriminate Analysis (LDA) | NR | NR |
| Prabhashwaree and Wagarachchi [58] | Imputation (mean) | MinMaxScaler (ANFIS-GA), StandardScaler (FFANN) | | Resampling (feature scaling) | NR | NR | NR |
| Prabhashwaree and Wagarachchi [59] | Imputation (mean) | MinMaxScaler (ANFIS-GA), StandardScaler (FFANN) | | NR | NR | target encoding | Correlation analysis (spearman) |
| Qasrawi et al [60] | Imputation | NR | | Resampling (evaluation metrics like MCC) | NR | NR | Correlation analysis (spearman) |
| Raisa et al [61] | NR | NR | | Resampling (SMOTE) | NR | Label encoding | NR |
| Reps et al [62] | Imputation (Mode) | NR | | NR | NR | NR | Correlation analysis |
| Shen et al [63] | NR | NR | | Mechanisms (reinforcement learning), Reweighting | NR | Label encoding | SHAP values (Differential Evolution) |
| Shin et al [64] | Excluded outliers | text cleaning | | Resampling (SMOTE) | Relief algorithm (reduced features from 126 to 99) | Label encoding | SHAP values (Differential Evolution) |
| Shivaprasad et al [65] | Imputation (median) | text cleaning | | NR | NR | One-hot encoding | Recursive feature elimination, GlmStepAIC, bagging-based selection-by-filter methods |
| Srivatsav and Nanthini [66] | Imputation (median) | Embedding layer | | NR | Spatial feature extraction | Label encoding | Chi-square test |
| Suganthi and Geetha [67] | Imputation | Normalization, text cleaning, Tokenization | | NR | Latent Semantic Analysis (LSA) | Label encoding | NR |
| Susič et al [68] | NR | Normalization, Scaling | | NR | NR | Label encoding | NR |
| Tang et al [69] | NR | Normalization | | Mechanisms (Proximal Policy Optimization (PPO)) | NR | NR | NR |
| Tortajada et al [70] | Imputation (mean) | Normalization, Pruning | | Resampling (metrics like geometric mean and AUC) | NR | Dummy encoding | NR |
| Valavani et al [71] | NR | Standardization, normalization | | Resampling ( feature selection) | Dimension reduction | NR | NR |
| Valdeolivar-Hernandez et al [72] | NR | Tokenization, lemmatization, stopword removal | | Handled by clustering participants into low, medium, and high-score groups | Latent Dirichlet Allocation (LDA) | NR | ReliefF, Tree-based importance, Boruta |
| Wagay [73] | Imputation | NR | | Resampling (SMOTE) | NR | Label encoding | NR |
| Wakefield and Frasch [74] | Excluded outliers | Normalization | | NR | NR | Binary encoding | Recursive Feature Elimination (RFE), Tree-based importance |
| Wang et al [78] | Imputation (mean, mode), Excluded | Normalization (Minmaxscaler), Data cleaning | | NR | NR | Dummy encoding | Recursive Feature Elimination (RFE) |
| Wang et al [76] | Excluded outliers | Standardization | | Oversampling of the minority class | NR | Binary encoding (Univariate Logistic Regression (p < 0.05), median frequency filtering) | Gini Index or Mean Decrease in Impurity (MDI) |
| Wang et al [75] | Excluded outliers | Normalization, standardization | | NR | Dimension reduction (SFFS) | NR | L1/L2 Regularization |
| Wang et al [77] | Excluded outliers | NR | | NR | Principal Component Analysis (PCA) | NR | Sequential Floating Forward Selection (SFFS) |
| Xu et al [79] | Excluded outliers | Standard-scale normalization | NR | | NR | NR | Lasso |
| Xu and Sampson [80] | Imputation(KNN) | Normalization, Partial dependence plots | | NR | NR | NR | NR |
| Yu et al [81] | Excluded GC-MS data (>50%) | Normalization | | NR | Principal Component Analysis (PCA), Latent Structures Discriminant Analysis (OPLS-DA) | NR | NR |
| Zhang et al [82] | Imputation | Normalization | | NR | Dimension reduction | NR | NR |
| Zhang et al [83] | NR | NR | | NR | NR | NR | Tree-based importance, Filter feature selection (FFS-RF) |
| Zhang et al [84] | Imputation (mean) | Normalization | | NR | NR | Dummy encoding | L1/L2 Regularization |
| Zhu et al [85] | Imputation | Normalization | | NR | NR | NR | Sequential Forward Selection (SFS) |
| NR: Not reported |  |  | |  |  |  |  |
