# Supplementary material for: AI for Detecting and Predicting Postpartum Depression: Scoping Review
Source: J Med Internet Res. 2026 Jan 8;28:e77376. doi: 10.2196/77376 (PMC12782538; doi:10.2196/77376)
Supplement: Multimedia Appendix 6 [file jmir-v28-e77376-s006.docx]

**Multimedia Appendix 6.** Characteristics of features.

| Study [References] | Data Types | Nr of Features | Data Input |
| --- | --- | --- | --- |
| Ajay et al [24] | obstetric, sociodemographic, psychological | 11 | Age, Antidepressant usage, Education level, Gestational age, Gravida, Medicine intake, Depression during pregnancy, Weight. |
| Sharma et al [21] | sociodemographic, psychological | 11 | Age, Crying, Maternal anxiety, Feeling of guilt, Feeling sad, Irritable towards baby & partner, Overeating or loss of appetite, Problems bonding with baby, Problems concentrating, Suicide attempt, Trouble sleeping at night. |
| Andersson et al [22] | medical history, obstetric, sociodemographic, psychological, behavioral, neonatal | 30 | Age, Alcohol use, Allergies, BMI, Contact with a psychiatrist, Contact with a psychologist, Depression history, Education level, Employment status, Endocrine problems, Height, History of abortions, Hypertension, Hypomanic episodes, Intimate partner violence, Irritable bowel syndrome, Marital status, Migraine, Pain problems, Parity, Premenstrual syndrome history, Residence, Sleep before pregnancy, smoking status, and Snuff before pregnancy. |
| Betts et al [23] | medical history, obstetric, sociodemographic, psychological | 20 | Age, Breastfeeding status, Cognitive and/or behavioral therapy, Crisis situation/event counselling, Enrolled as a private patient, Indigenous status, Mental or nervous disorder impacting pregnancy, Neuraxial block, Obstetric complications, Personal history of other mental or behavioral disorders, Postpartum hemorrhage, Prenatal anxiety disorder diagnosis, Prenatal psychotic and bipolar disorders admission, Prenatal psychotic and bipolar disorders diagnosis, Prenatal substance use disorders admission, Prenatal unipolar mood disorder admission, Primary prenatal health care provider – general practitioner, Prior psychiatric hospitalizations, smoking status. |
| Cai et al [25] | behavioral | 10 | Body image change, Cognitive impairment, Contemplating, Emotional lability, Feeling of guilt, Loss of self, Maternal anxiety, Overeating or loss of appetite, Self-harm tendency, Sleeping disorders, Weight loss, and Work difficulty. |
| Carneiro et al [26] | psychological, linguistic | 2 | Happiness Phrases, Sadness Phrases. |
| Chen et al [27] | linguistic | 10 | Coping ability, Crying, Emotions, Fear, Fun, Feeling of guilt, Sleeping disorders, Maternal anxiety, feeling sad, Self-inflict. |
| Fanos et al [28] | sociodemographic, psychological, neonatal | 10 | Age, Breastfeeding status, Education level, Employment status, Health of baby, Nationality, Negative emotions, Positive emotions, Support network. |
| Fatima et al [29] | psychological, linguistic | 11 | Absolutist words, Drives and needs, Function words, Other grammar, Perceptual processes, Personal concerns, Psychological processes, Social words, Summary variables, Time orientations, Word count (WC). |
| Fazraningtyas et al [30] | obstetric, sociodemographic, psychological, neonatal | 31 | Age, Birth weight, Blood type, Bond with husband, Desire to be pregnant, Duration of delivery, Education level, Ethnicity, Fear of delivery, Gestational age, Gravida, Health of baby, History of abortion, Depression history, Depression history in the family, Knowledge of parenting, Marital status, Medicine intake, Menstrual problems, Mode of delivery, Monthly income, Newborn gender, Number of children, Employment status, Parity, Position during pregnancy, Relationship with family, Religion, Support network, Tradition in family, Weight gain. |
| Gabrieli et al [31] | sociodemographic, linguistic | 8 | Age, Formant frequencies 1–4, Fundamental frequency, Intensity, Newborn gender. |
| Gopalakrishnan et al [34] | obstetric, sociodemographic , psychological, behavioral | 26 | Age, Birth weight, Breastfeeding status, being worried about returning to work, Education level, Gestational age, History of anxiety, Depression history, Induction of labour, Issues with infertility, Marital status, Maternal tolerance, Mode of delivery, Monthly income, Motherhood feeling, Number of children, Obstetric complications, Relationship With Family, Paternal thoughts on pregnancy, Planned pregnancy, Postpartum depression history, Antidepressant usage, Ready to leave the hospital, Residence, Work stress. |
| Gopalakrishnan et al [33] | linguistic | 4 | Depressive symptom words, Psycholinguistic style, Ruminative response style, Sentimental words. |
| Gopalakrishnan et al [32] | sociodemographic, psychological, behavioral | 11 | Age, Being worried about returning to work, Current life stressors, Education level, Maternal tolerance, Mode of delivery, Planned pregnancy, Relationship with family, Ready to leave the hospital, and Socio-demographic factors. |
| Gupta et al [35] | sociodemographic, psychological | 10 | Age, Crying,Maternal anxiety, Feeling of guilt, Feeling sad, Irritability towards the baby and partner, Overeating or loss of appetite, Problem in decision-making, Problems bonding with baby, Problems concentrating, Trouble sleeping at night. |
| Horgen [36] | obstetric, sociodemographic, psychological, behavioral | 25 | Abuse, Alcohol use, Civil status, Drug use, Education level, Experience of assault, Experience of specific emotions, Feelings about weight, Height, weight, Housing, Life events after delivery, Lifetime history of major depression, Living situation, Monthly income, Native language, Parity, Relationship satisfaction, Satisfaction with life, Self-efficacy, Self-esteem, Sick leave, Social support, Work situation, and Work stress. |
| Hurwitz et al [37] | sensor-based | 16 | Activity intensity, Calories burned, Heart rate, Sum of steps. |
| Jimenez-Serrano et al [38] | sociodemographic, psychological, neonatal | 11 | Age, Depressive symptoms, Educational level, Emotional lability, Labor situation during pregnancy, Life events after delivery, Monthly income, Newborn gender, Neuroticism, Number of family members living together, Psychiatric history. |
| Krishnamurti et al [39] | linguistic | 354 | Daily mood ratings, LDA topic modeling, LIWC features, Sent wordnet features, Word2Vec features. |
| Lilhore et al [41] | obstetric, sociodemographic, psychological | 16 | Age, Birth week, Birth weight, Education level, Depression history, Hospital distance, Induction of labor, Issues with infertility, Marital status, Mode of delivery, Monthly income, Motherhood feelings, Number of children, Paternal thoughts on pregnancy, Relationship with family, Work stress, Being worried about returning to work. |
| Lilhore et al [40] | sociodemographic, psychological | 9 | Age, Crying, Maternal anxiety, Feeling of guilt, Feeling sad, Irritable towards baby and partner, Overeating or loss of appetite, Problem in decision-making, Problems bonding with baby, Problems concentrating, Trouble sleeping at night. |
| Liu et al [43] | medical history, obstetric, sociodemographic, psychological, behavioral | 31 | Age, Bipolar disorder history, Diagnoses, Domestic violence, Gestational diabetes, History of abortions, History of anxiety, Depression history, Hypertension, Insurance type, Lab results, Marital status, Maternal mental health history, Medication prescriptions, Medications, Obstetric complications, Employment status, Parity, Postpartum hemorrhage, Pre-eclampsia, Preterm birth history, Prior psychiatric hospitalizations or self-harm tendency, Schizophrenia or psychotic disorders, Social support indicators, Socioeconomic status, Substance use disorder history, Use of anti-anxiety medications, Use of antipsychotic medications, Antidepressant usage. |
| Liu et al [42] | sociodemographic, psychological, behavioral | 6 | Domestic violence, Emotional lability, Emotional well-being, Relation with in-laws, Self-harm tendency. |
| Lyall et al [44] | obstetric, sociodemographic, psychological, behavioral, sensor-based | 51 | Greater inactivity during the daytime, Depression history, Individuals experiencing difficulty getting up in the morning, Lower morning activity, Sleep disorders, and Worse depression outcomes. |
| Marshad et al [45] | sociodemographic, psychological | 9 | Age, Crying, Maternal anxiety, Feeling of guilt, Feeling sad, Irritable towards baby and partner, Overeating or loss of appetite, Problem in decision-making, Problems bonding with baby, Problems concentrating, Trouble sleeping at night. |
| Matsumura et al [46] | medical history, obstetric, sociodemographic, psychological, biomarker | 84 | Alcohol use, Antibacterial drug use, Autistic traits, Bodily pain, Diastolic blood pressure, Education level, Emotional social support, Employment status, Fish intake, Frequency of taking folic acid supplements, General health perception, Generalized trust, History of physician-diagnosed anxiety disorder, History of physician-diagnosed autism, Asperger’s syndrome, or pervasive developmental disorder, History of physician-diagnosed depression, History of physician-diagnosed dysautonomia, History of physician-diagnosed learning disability, History of physician-diagnosed schizophrenia, History of taking oral contraceptives, Hopelessness, Husband's education, Husband's smoking status, Infertility treatment before current pregnancy, Intimate partner violence, Living situation, Marital status, Maternal age, Maternal feelings, Maternal income, Maternal smoking status, Mental health, Morning sickness, Neighborhood trust, Nervousness, Overall feeling when waking up during the past month, Parity, Physical activity, Physical functioning, Pre-pregnancy BMI, Pregnancy complications, Psychotropic drug use, Residence, Role emotional, Role physical, Serum insulin-like growth factor-1 concentration, Sleep depth during the past month, Sleeping soundly until morning during the past month, Stressful life events, Systolic blood pressure, Total blood cholesterol, Total blood triglycerides, Total energy intake, Trouble falling asleep, Sleeping disorders, Vitamin D intake, Vitality, and Residence, Social functioning. |
| Matsuo et al [47] | medical history, obstetric, sociodemographic, behavioral, neonatal | 41 | Age, Alcohol use, Apgar scores, Birth weight, Blood loss, Breastfeeding status, Congenital abnormalities, Duration of education, Epidural delivery, Gestational age, Height, History of mental disorders, History of spontaneous abortion, Husband’s age, Husband’s smoking during pregnancy, Hypertensive disorders, Induction of labor, Infertility treatment type, Marital status, Maternal transfer after delivery, Mental disorder, Mode of delivery, Newborn gender, Number of abortions, Perineal laceration severity, Placenta abruption, Placenta previa, Primipara status, Prolonged labor, Sleep duration, smoking status, Sleeping disorders, Umbilical arterial blood pH, Weight, and Weight gain. |
| Mazumder and Baruah [48] | obstetric, sociodemographic, psychological | 31 | Age, Anger issues, Bond with husband, Breastfeeding status, Depression history, Education level, Employment status, Family type, Hospital type, Loss of appetite, Maternal anxiety, Marital status, Medicine intake, Mode of delivery, Overeating or loss of appetite, Planned pregnancy, Preterm birth, Problem in decision-making, Problems bonding with baby, Residence, and Sleeping tendency. |
| Moreira et al [49] | medical history, obstetric, sociodemographic | 19 | Age, Blurred vision, Edema, Family history of hypertensive disorders, Gestational age, Gestational diabetes, Gravida, Headache, HELLP syndrome indicators, Hemolysis, High blood pressure, Hypertension, ICU admission, Lifestyle factors, Nausea, Oliguria, Proteinuria, Symptoms experienced during pregnancy, Thrombocytopenia. |
| Mustafa [50] | sociodemographic, psychological, behavioral, neonatal | 9 | Age, Educational level, Emotional lability, Labor situation during pregnancy, Life events after delivery, Monthly income, Newborn gender, Number of family members living together, Psychiatric history. |
| Myneni et al [51] | obstetric, psychological, behavioral, sensor-based | 14 | Accessing patient portals for healthcare records, Automated annotation, Blood glucose/blood pressure logs, Depression literacy assessments, Health education modules, Image-based health library, Maintaining a glucose diary, PPD 101 educational videos, Pregnancy tracker app for monitoring health metrics, Social support forums, Storing and managing personal health data through a personal storage app, Support network and information pamphlets, Tracking and managing medications, and Utilizing web and social media resources for health-related information. |
| Nasim et al [52] | sociodemographic, psychological | 9 | Age, Crying,Maternal anxiety, Feeling of guilt, Feeling sad, Irritable towards baby and partner, Overeating or loss of appetite, Problem in decision-making, Problems bonding with baby, Problems concentrating, Trouble sleeping at night. |
| Natarajan et al [53] | Sociodemographic, psychological, behavioral, neonatal | 46 | Breastfeeding status, Child care stress, Health of baby, History of anxiety, Depression history, Infant temperament, Maternity blues, Marital status, Maternal mental health, Self-esteem, Social support, Stressful life event,Sleeping disorders, Unplanned pregnancy, and Worry for baby. |
| Osubor and Egwali [54] | psychological | 6 | Feeling sad, Lack of interest in activities previously enjoyed, Problem in decision-making,Sleeping disorders, Suicide attempt, Worries about harming the baby or partner. |
| Park et al [55] | obstetric, sociodemographic, behavioral | 18 | Age, Any mental health-related visits, Baseline service utilization factors like routine pregnancy screenings, psychiatric therapy or counseling, outpatient visits, and emergency department visits, Depression history, HEDIS-qualifying visits, Insurance plan, Mode of delivery, Opioid use disorders, Postpartum depression, Substance use disorders, Bipolar disorders, and Anxiety-related disorders. |
| Paul et al [56] | medical history, obstetric, sociodemographic, behavioral, neonatal | 12 | Asthma, BMI, Breastfeeding status, Depression, Diabetes, Hypertension, Infant sleep patterns, Intimate partner violence, Maternal weight before pregnancy, Nutrition status, Oral health, Social support, and Unwanted pregnancy. |
| Payne et al [57] | obstetric, psychological, biomarker | 6 | Antenatal depression status (estimated via biomarkers), Cell type ratios, DNA methylation,psychiatric history. |
| Prabhashwaree and Wagarachchi [58] | obstetric, sociodemographic, behavioral | 18 | Age, Covid-19 exposure during pregnancy/postpartum, Current life stressors, Education level, Family relationship issues, Fear of delivery, History of drug abuse, Loss during pregnancy, Marital status, Newborn gender, Neonatal intensive care unit (NICU), Number of children, Preterm birth, Serious physical disability after childbirth, Socioeconomic status, Support network. |
| Prabhashwaree and Wagarachchi [59] | obstetric, sociodemographic, behavioral | 18 | Age, Covid-19 exposure during pregnancy/postpartum, Current life stressors, Education level, Family relationship issues, Fear of delivery, History of drug abuse, Loss during pregnancy, Marital status, Newborn gender, Neonatal Intensive Care Unit (NICU), Number of children, Preterm birth, Serious physical disability after childbirth, Socioeconomic status, Support network. |
| Qasrawi et al [60] | obstetric, sociodemographic, psychological, behavioral, sensor-based | 23 | Age, Age at marriage, Cancellation of clinic visits due to lockdown, Covid-19 exposure during pregnancy/postpartum, Education level, Family relationship issues, Financial problems, Food consumption patterns, Gravida, Health problems, Locality, Monthly income, Physical activity, Psychological problems, Residence, Sleep duration, smoking status, Social problems, Stress during pregnancy, Work status, Work stress. |
| Raisa et al [61] | obstetric, sociodemographic, psychological, behavioral, neonatal | 46 | Age range, Abuse, Age of latest child, Age of previous baby, Addiction, Anger issues, Problems bonding with baby, Bond with husband, Breastfeeding status, Checkups during pregnancy, Current Employment status, Disease before pregnancy, Diseases during pregnancy, Education level, Family type, Fear of pregnancy, Gestational age, Husband and baby relationship, Husband’s education, Husband’s income, Income before pregnancy, Loss during pregnancy, Marital status, Mode of delivery, Motherhood feeling, Monthly income, Newborn gender, Number of children, Number of family members living together, Number of latest pregnancies, Obstetric complications, Planned pregnancy, Psychological resilience, Received support, Relation with in-laws, Residence, Rest when baby sleeps, Rest while baby is monitored, Sharing feelings with friends, Want support, Worry for baby, Work feeling after delivery. |
| Reps et al [62] | sociodemographic, psychological | 202 | Fear, Maternal anxiety, and Sleeping disorders. |
| Shen et al [63] | medical history, obstetric, sociodemographic, psychological, behavioral, neonatal | 30 | Age, Alcohol use, Allergies, BMI, Contact with psychiatrist, Contact with psychologist, Depression history, Education level, Employment status, Endocrine problems, Height, History of abortions, Hypertension, Hypomanic episodes, Intimate partner violence, Irritable bowel syndrome, Marital status, Migraine, Pain problems, Parity, Premenstrual syndrome history, Residence, Sleep before pregnancy, smoking status, and Snuff before pregnancy. |
| Shin et al [64] | obstetric, sociodemographic, psychological, behavioral, neonatal | 47 | Age, Alcohol use, Attendance at childbirth classes, Birth weight, BMI, Depression before pregnancy, Dental care advice, Dental hygiene before pregnancy, Drinking behavior three months before pregnancy, Education level, Employment status, Ethnicity, Family relationship issues, Financial problems, Flu vaccination during pregnancy, Health worker discussions on depression screening, Height, History of abortions, HIV testing, Insurance type, Kotelchuck Index, Marital status, Maternal work hours during pregnancy, Medical risk factors, Mode of survey contact, Month of last menstrual period, Monthly income, Multivitamin use, Need for nutritional counseling during pregnancy, Need for smoking cessation support during pregnancy, Newborn gender, Number of dependents, Parity, Perceived discrimination in healthcare, Physical activity, Postpartum dieting, Pre-pregnancy anxiety screening, Pre-pregnancy exercise, Pregnancy intention, Prenatal care visits, smoking status, Stress from substance abuse, Total stress during pregnancy, Weight, Weight gain, Breastfeeding status, and Work stress. |
| Shivaprasad et al [65] | sociodemographic, psychological | 9 | Age, Crying, Maternal anxiety, Feeling of guilt, Feeling sad, Irritable towards baby and partner, Overeating or loss of appetite, Problem in decision-making, Problems bonding with baby, Problems concentrating, Suicide attempt, Trouble sleeping at night. |
| Srivatsav and Nanthini [66] | linguistic, sensor-based | 7 | Metadata tags, Polarity of the tweet, The query, The tweet text, The tweet's date, The tweet's ID, The user who tweeted. |
| Suganthi and Geetha [67] | medical history, obstetric, sociodemographic, psychological, behavioral, linguistic | 10 | Demographic status, Mental health ,Behavioral changes, Mental health history, Vital signs . |
| Susič et al [68] | medical history, obstetric, sociodemographic, biomarker | 22 | Age, BMI, C-reactive protein (CRP), Education level, Ferritin, Gestational age, Gravida, Hemoglobin levels, History of abortions, Marital status, Medication type (oral or intravenous iron supplementation), Mode of delivery, Number of children, Parity, Phosphate, Serum iron, Total iron-binding capacity (TIBC), Transferrin saturation, Transfusion status (binary). |
| Tang et al [69] | obstetric, sociodemographic , biomarker | 5 | Age, Depression history, Neuroimaging data, Obstetric complications, Socioeconomic status. |
| Tortajada et al [70] | obstetric, sociodemographic, psychological, neonatal | 16 | Age, Depressive symptoms, Educational level, Emotional lability, Employment status, Life events after delivery, Life events during pregnancy, Marital status, Medical perinatal risk, Monthly income, Newborn gender, Neuroticism, Number of children, Number of people living together, Psychiatric history, Social support. |
| Valavani et al [71] | medical history, obstetric, sociodemographic, psychological, neonatal | 13 | Age, Apgar scores, BMI, Birth weight, Bronchopulmonary dysplasia, Days of intubation, Days of supplemental oxygen, Educational level, Ethnicity, Gestational age, Infant feeding at discharge, Maternal anxiety, Maternal asthma, Occipitofrontal circumference, Previous live births, Retinopathy of prematurity, Socioeconomic status, Temperament traits. |
| Valdeolivar-Hernandez et al [72] | psychological, neonatal | 30 | Words |
| Wagay [73] | psychological | 5 | Feeling of guilt, Irritable towards baby and partner, Overeating or loss of appetite, Problem in decision-making, Problems bonding with baby, Problems concentrating. |
| Wakefield and Frasch [74] | obstetric, sociodemographic, psychological | 9 | Age, Being worried about returning to work, Current life stressors, Education level, Maternal tolerance, Mode of delivery, Planned pregnancy, Relationship with family, Ready to leave the hospital, and Socio-demographic factors. |
| Wang et al [78] | medical history, obstetric, sociodemographic, psychological, neonatal | 15 | Apgar scores, Birth weight, Breastfeeding status, Gestational age, Gestational diabetes, Hypertension, Maternal mental health history, Mode of delivery, Monthly income, Parity, Postpartum hemorrhage, Socioeconomic factors, Weight. |
| Wang et al [76] | medical history, obstetric, sociodemographic, psychological | 26 | Abdominal pain, Anti-inflammatory agents, Antidepressant usage, Backache, Depressive disorder, Diarrhea, Ethnicity, History of spontaneous abortion, Hypertensive disorder, Major depression, Marital status, Maternal anxiety, Mental disorder, Muscle pain, Obesity, Palpitations, Preterm birth, Vomiting in pregnancy, and Weight gain. |
| Wang et al [75] | linguistic | 988 | Derivatives and statistical measures, Fundamental frequency (F0), F0 envelope, Intensity, Linear spectral pair (LSP) frequencies, Loudness, Mel-frequency cepstral coefficients (MFCC), Voicing probability, Zero cross rate. |
| Wang et al [77] | obstetric, sociodemographic, biomarker | 32 | Age, Birth weight, BMI, Height, Neonatal intensive care unit, Preterm birth, Protein-protein interaction, Proteomics, Socioeconomic status, Weight. |
| Xu et al [79] | biomarker | 6 | Degree Centrality (DC), Low-Frequency Fluctuation (ALFF), Regional homogeneity (Reho), The Left Inferior Occipital Gyrus (IOG.L), The Right Anterior Cingulate Cortex (ACC.R). |
| Xu and Sampson [80] | medical history, obstetric, sociodemographic, psychological, behavioral | 34 | Age, Attendance at childbirth classes, BMI, Current life stressors, Depression during pregnancy, Diabetes, Education level, Ethnicity, Gestational diabetes, Healthcare utilization patterns, Holding back questions or concerns during provider communication, Hypertension, Insurance status, Mode of delivery, Need for nutritional counseling during pregnancy, Need for smoking cessation support during pregnancy, Number of children, Parity, Patient-provider communication scores, Perceived discrimination, Perceived quality of maternity care in the U.S., Planned pregnancy, Postpartum pain scores, Pre-pregnancy health conditions, Satisfaction with U.S. medical care, Self-rated health status, Socioeconomic status, Time of prenatal care initiation, and Use of pain relief medications during childbirth. |
| Yu et al [81] | biomarker | 170 | Alanine, aspartate, and glutamate metabolism, Aminoacyl-tRNA biosynthesis, Arginine biosynthesis, Azelaic acid, Cysteine, Citrate cycle, Cholesterol, Cytosine and erythrulose, Glutamic acid, Hydroxylamine, Monostearin and phenylacetic acid, Pantothenate and CoA biosynthesis, Postpartum metabolites, Tryptophan, Valine, leucine, and isoleucine biosynthesis. |
| Zhang et al [82] | sociodemographic, psychological, behavioral | 25 | Age, Bond with husband, Childhood abuse history, Education level, Depression history, Depression history in the family, Husband’s education, Husband’s monthly income, Monthly income, Mother’s menopausal symptoms, maternal mental health history, History of family members, Psychiatric history, Premenstrual syndrome history, Psychological resilience,Intimate partner violence. |
| Zhang et al [83] | medical history, obstetric,sociodemographic, psychological | 32 | Abdominal pain, Abnormality of organs and/or soft tissues of pelvis affecting pregnancy, Acute pharyngitis, Antidepressant usage, Antihistamines for systemic use, Beta blocking agents, Diarrhea, Diastolic blood pressure, Direct acting antivirals, Emergency department visits, False labor at or after 37 completed weeks of gestation, Hemorrhage in early pregnancy antepartum, History of anxiety, Hypertensive disorder, Hypothyroidism, Marital status, Maternal anxiety, Mental disorder, Migraine, Mode of delivery, Mood disorder history, Other antibacterials, Other disorder history, Palpitations, Placental infarct, Pre-eclampsia, Primigravida, Spontaneous abortion, and Vomiting in pregnancy. |
| Zhang et al [84] | medical history, obstetric, sociodemographic, psychological | 32 | Abdominal pain, Acute pharyngitis, Abnormality of organs and/or soft tissues of pelvis affecting pregnancy, Anxiety in pregnancy, Antidepressant usage, Antihistamines for systemic use, Beta blocking agents, Depression during pregnancy, Diarrhea, Diastolic blood pressure in third trimester, Direct acting antivirals, Emergency department visits, Ethnicity, False labor at or after 37 completed weeks of gestation, Hemorrhage in early pregnancy antepartum, History of anxiety, Hypertensive disorder, Hypothyroidism, Marital status, Mental disorder, Migraine, Mode of delivery, Mood disorder history, Other antibacterials, Other disorder history, Palpitations, Placental infarct, Pre-eclampsia, Primigravida, Spontaneous abortion, and Vomiting in pregnancy. |
| Zhu et al [85] | medical history, obstetric, sociodemographic, neonatal | 19 | Age, Bond with husband, Breastfeeding status, Diabetes, Education level, Employment status, Health of baby, Iron deficient anemia, Marital status, Mode of delivery, Monthly income, Newborn gender, Obstetric complications, Parity, Planned pregnancy, Residence, Thyroid function, Obesity. |
| NR: Not reported |  |  |  |
