# Supplementary material for: AI for Detecting and Predicting Postpartum Depression: Scoping Review
Source: J Med Internet Res. 2026 Jan 8;28:e77376. doi: 10.2196/77376 (PMC12782538; doi:10.2196/77376)
Supplement: Multimedia Appendix 7 [file jmir-v28-e77376-s007.docx]

**Multimedia Appendix 7**. Characteristics of artificial intelligence.

| Study [References] | | Aim of AI algorithm | AI category | Problem solving approach | Algorithms | AI Model Type | Best-Performing Classification Model | Validation Techniques | ML Performance measures | Optimization Strategies & Regularization & Model Stabilization |
| --- | --- | --- | --- | --- | --- | --- | --- | --- | --- | --- |
| Ajay et al [24] | | Prediction | ML | Classification | Ensemble Methods (Bagging) | RF, DT, XGBoost | Random Forest | Hold-out | Accuracy,Precision, Sensitivity, F_1 score, AUC | NR |
| Sharma et al [21] | | Prediction | ML | Classification | Ensemble Methods (Boosting) | ANN, DT, KNN, LogR, SVM, RF, XGBoost, Stacking Ensemble | Stacking Ensemble | Hold-out | Accuracy, Precision, Sensitivity, F1-Score | NR |
| Andersson et al [22] | | Prediction | ML | Classification | Ensemble Methods (Bagging) | GB, LASSO, Ridge, XRT | Decision Tree | K-fold-Cross-validation | Accuracy, Sensitivity, Specificity, PPV, NPV, AUC | Tunning Parameter |
| Betts et al [23] | | Prediction | ML | Classification | Ensemble Methods (Boosting) | EN, GB, LogR | Gradient Boosting | K-fold-Cross-validation | AUC | Learning Rate, L1/L2 regularisation, Tunning Parameter |
| Cai et al [25] | | Prediction | ML | Classification | Ensemble Methods (Bagging) | DT | Decision Tree | K-fold-Cross-validation | Precision, Sensitivity, AUC | NR |
| Carneiro et al [26] | | Prediction | DL\TL | Classification | Neural Networks (NN) | LSTM, SVM | LSTM | Hold-out | Accuracy, Precision, Sensitivity, F_1 score | NR |
| Chen et al [27] | | Prediction | DL | Classification | Neural Networks (NN) | LSTM, Attention mechanism | LSTM | Hold-out | Accuracy | Adam, Dropout, Learning Rate, SGD, Attention Mechanism, binary_crossentropy |
| Fanos et al [28] | | Detection | DL | Classification | Neural Networks (NN) | NN | Neural Networks (NN) | Hold-out | Accuracy | AdamW, Early Stopping, L1/L2 Regularization, SGD |
| Fatima et al [29] | | Prediction | ML | Classification | Neural Networks (NN) | LogR, ML, SVM | ML | K-fold-Cross-validation, Hold-out | Accuracy,Precision, Sensitivity | L1/L2 regularization, LASSO, Learning Rate, SGD, Tuning Parameter |
| Fazraningtyas et al [30] | | Prediction | ML | Classification | Ensemble Methods (Boosting) | DT, GB, XGBoost | XGBoost | K-fold-Cross-validation | Accuracy, Precision, Sensitivity, Specificity | NR |
| Gabrieli et al [31] | | Detection | ML | Classification | Neural Networks (NN) | NN | Neural Networks (NN) | Hold-out | Accuracy, Precision, Sensitivity, Specificity, AUC | NR |
| Gopalakrishnan et al [34] | | Prediction | ML | Classification | Ensemble Methods (Bagging) | Lasso, Ridge, Naive Bayes, RF | Random Forest | Hold-out | Accuracy, Sensitivity, Specificity, PPV, NPV, AUC | Tunning Parameter |
| Gopalakrishnan et al [33] | | Prediction | ML\NLP | Classification | Classification | LogR, SVM, RF, ADAboost | Support Vector Machine | Hold-out | Accuracy, Precision, Sensitivity, F_1 score | NR |
| Gopalakrishnan et al [32] | | Prediction | DL\ML | Classification | Neural Networks (NN) | RNN , SVM, Naive Bayes | Recursive Neural Network (RNN) | Hold-out | Accuracy ,Precision, Sensitivity, PPV, NPV, F_1 score | Adam, Learning Rate,SGD, Tuning Parameter (Grid Search), Attention Mechanism |
| Gupta et al [35] | | Prediction | ML | Classification | Ensemble Methods (Boosting) | CatBoost, LightGBM | CatBoost | K-fold-Cross-validation | Accuracy, Precision, Sensitivity , F1-Score | Tunning Parameter |
| Horgen [36] | | Prediction | ML | Regression | Ensemble Methods (Boosting) | EN, NN, XGBoost | XGBoost | Hold-out | MSE, RMSE, MAE | SGD, Adam, Batch Norm, Dropout, Early Stopping, Momentum, L1/L2 Regularization, SGD, Weight Decay, Tuning Parameter (Grid Search) |
| Hurwitz et al [37] | | Detection | ML | Classification | Ensemble Methods (Bagging) | KNN, GLM, RF, SVM | Random Forest | K-fold-Cross-validation | Precision, Sensitivity, Specificity, AUC | NR |
| Jimenez-Serrano et al [38] | | Detection | ML | Classification | Probabilistic Classification | Naive Bayes, SVM, ANN, LogR | Naive Bayes | Hold-out | Accuracy, Sensitivity, Specificity, AUC, G-Mean | Cutoff point, Tuning Parameter (Grid Search) |
| Krishnamurti et al [39] | | Prediction | ML\NLP | Classification | Linear Regression (LinR) | LASSO | LASSO | K-fold-Cross-validation, Hold-out | AUC | NR |
| Lilhore et al [41] | | Prediction | DL\ML\TL | Classification | Neural Networks (NN) | AdaBoost, DT, LSTM (TL), RF, XGBoost, CatBoost | LSTM (TL) | Hold-out | Accuracy,Precision, Sensitivity, F_1 score, AUC | Adam, Learning Rate, Batch Norm, Dropout, Batch size |
| Lilhore et al [40] | | Prediction | DL\TL | Classification | Neural Networks (NN) | LSTM ( Attention Mechanisms), CNN | LSTM | Hold-out | Accuracy,Precision, Sensitivity, F_1 score, AUC | AdamW, Batch Normalization Layer, Cosine Annealing, Dropout, Fully linked Layer, RMSProp, SGD, Attention Mechanism |
| Liu et al [43] | | Prediction | ML | Classification | Ensemble Methods (Boosting) | LogR, MLP, RF, SVM, XGBoost | XGBoost | K-fold-Cross-validation, Hold-out | Accuracy,Precision, Sensitivity, AUC | Tunning Parameter ( Grid Search) |
| Liu et al [42] | | Prediction | ML | Classification | Probabilistic Classification | LogR | Logistic Regression | Hold-out | Sensitivity, Specificity, AUC | Cuttof Point, L1/L2 Regularization |
| Lyall et al [44] | | Prediction | ML | Regression | Linear Regression (LinR) | Lasso, LogR, Ridge, EN | LASSO | Nested Cross-Validation | Accuracy, Sensitivity, Specificity, PPV, NPV, AUC | Cutoff point, L1/L2 Regularization, Tuning Parameter (Grid Search) |
| Marshad et al [45] | | Prediction | ML | Classification | Ensemble Methods (Boosting) | XGBoost, LightGBM, CatBoost, AdaBoost | XGBoost | Hold-out | Accuracy, Sensitivity, Specificity, PPV, NPV, AUC | NR |
| Matsumura et al [46] | | Prediction | ML | Classification | Ensemble Methods (Bagging) | DT | Decision Tree | Hold-out | Accuracy, Sensitivity, Specificity, F_1 score, AUC | NR |
| Matsuo et al [47] | | Prediction | ML | Classification | Probabilistic Classification | EN, LogR, Ridge, RF, SVM | Logistic regression | K-fold-Cross-Validation, Hold-out | AUC | Tunning Parameter |
| Mazumder and Baruah [48] | | Detection | ML | Classification | Ensemble Methods (Bagging) | DT, RF, Ada Boost, Bagging | Random Forest | K-fold-Cross-Validation | Accuracy, Specificity, AUC | Adam, Cosine Annealing, Dropout |
| Moreira et al [49] | | Prediction | ML | Classification | Ensemble Methods (Bagging) | DT, KNN, SVM | Decision Tree | K-fold-Cross-Validation | Accuracy, Sensitivity, FPR, AUC | Tunning Parameter |
| Mustafa [50] | | Prediction | ML | Classification | Probabilistic Classification | LogR, Naive Bayes, SVM, ANN | Naive Bayes | Hold-out | Precision, Sensitivity, G-Mean, AUC | Tunning Parameter |
| Myneni et al [51] | | Detection | ML | Classification | NLP | DT, GB, KNN, RF, SVM, BERT-based | NLP( BERT) | Hold-out | Sensitivity, Specificityy, G-Mean, AUC | Tunning Parameter( cascading classifiers) |
| Nasim et al [52] | | Prediction | ML | Classification | Neural Networks (NN) | Ridge, DT, KNN, RF, MLP (Meta-Learning), Stacking Model | Stacking Model | K-fold-Cross-Validation | Accuracy, Sensitivity, F_1 Score | Tunning Parameter |
| Natarajan et al [53] | | Prediction | ML | Classification | Ensemble Methods (Boosting) | "AdaBoost, DT, Naive Bayes, LogR, SVM |  |  | Precision, Sensitivity, F_1 score, AUC | RMSProp |
| Osubor and Egwali [54] | | Prediction | ML | Regression | " | Gradient Boosting | K-fold-Cross-Validation, Hold-out |  | Accuracy, Sensitivity | Cuttof point |
| Park et al [55] | | Prediction | ML | Classification | Neural Networks (NN) | ANFIS | Artificial Neural Network (ANN) | Hold-out | Accuracy, Sensitivity, Specificity, AUC | NR |
| Paul et al [56] | | Detection | ML | Classification | Ensemble Methods (Boosting) | LogR, RF, XGBoost | XGBoost | Hold-out | Accuracy,Precision, Sensitivity, F_1 score, AUC | Adam, Learning Rate, SGD, Tuning parameter (Kernel Type), Weight Decay (decay rate), Dropout (Hidden Layer) |
| Payne et al [57] | | Prediction | ML | Classification | Neural Networks (NN) | AdaBoost, DT, GB, KNN, LogR, MLP, RF, SVM, TabN | TabNet | Hold-out | Accuracy,Precision, Sensitivity, F_1 score, AUC | NR |
| Prabhashwaree and Wagarachchi [58] | | Prediction | DL\ML | Classification | Classification | SVM | Support Vector Machine | LooCV | Accuracy, Sensitivity, F1-Score, MAE, MSE, RMSE | Tunning Parameter |
| Prabhashwaree and Wagarachchi [59] | | Prediction | DL\ML | Classification | Neural Networks (NN) | ANFIS-GA,FFANN, RF, SVM | Artificial Neural Network (ANN) | Hold-out | Accuracy,Precision, Sensitivity, F_1 score, AUC | Dropout, RMSProp, Categorical_crossentropy |
| Qasrawi et al [60] | | Prediction | ML | Classification | Neural Networks (NN) | ANFIS-GA, FFANN, RF, SVM | Artificial Neural Network (ANN) | Hold-out | Accuracy,Precision, Sensitivity, F_1 score, AUC | NR |
| Raisa et al [61] | | Detection | ML | Classification | Ensemble Methods (Boosting) | DT, GB, RF, Naive Bayes, SVM | Gradient Boosting | Hold-out | Accuracy,Precision, Sensitivity, F_1 score, AUC, G-Mean | NR |
| Reps et al [62] | | Prediction | ML | Classification | Ensemble Methods (Bagging) | GB, LogR, RF, SVM, XGBoost | Random Forest | K-fold-Cross-Validation | Accuracy,Precision, Sensitivity, F_1 score, G-means | NR |
| Shen et al [63] | | Detection | DL\RL | Classification | Ensemble Methods (Boosting) | GB | Gradient Boosting | K-fold-Cross-Validation | Accuracy, Sensitivity, Specificity | NR |
| Shin et al [64] | | Prediction | ML | Classification | Neural Networks (NN) | ANN, Reinforcement Learning | Artificial Neural Network (ANN) | K-fold-Cross-Validation | Accuracy, Precision, Sensitivity, Specificity, F_1 score, AUC | Dropout, SGD |
| Shivaprasad et al [65] | | Prediction | DL | Classification | Ensemble Methods (Bagging) | AdaBoost, KNN, LogR, NN,Naive Bayes,RF, SVM, Recursive Partitioning | Random Forest | K-fold-Cross-Validation | Accuracy,Precision, Sensitivity, F_1 score | Tunning Parameter ( Grid Search) |
| Srivatsav and Nanthini [66] | | Prediction | ML | Classification | Ensemble Methods (Boosting) | RF, LogR, DT, KNN, AdaBoost, CatBoost, LightGBM, XGBoost | Stacking Ensemble | K-fold-Cross-Validation | Accuracy | Adam, SGD, categorical cross-entropy |
| Suganthi and Geetha [67] | | Prediction | ML | Classification | Neural Networks (NN) | LSTM, Logistic Regression | LSTM | NR | Accuracy,Precision, Sensitivity, F_1 score | Tunning Parameter (Osprey Optimization Algorithm (OOA)) |
| Susič et al [68] | | Prediction | ML | Regression | Neural Networks (NN) | MLP | MLP | NR | MAE, RMSE | NR |
| Tang et al [69] | | Prediction & Detection | ML\RL | Classification | Linear Regression (LinR) | EN, RidgeR, KernelR, SVR, GB, XGBoost, LightGBM, CatBoost, LR | Elastic Net Regression | K-fold-Cross-Validation | Accuracy, F1-Score, G-Mean | Mutual Learning-based Artificial Bee Colony (ML-ABC) |
| Tortajada et al [70] | | Detection | ML | Classification | Ensemble Methods (Bagging) | ANN | Artificial Neural Network (ANN) | K-fold-Cross-Validation | Accuracy, Sensitivity, Specificity, G-mean, AUC | Tunning Parameter |
| Valavani et al [71] | | Detection | DL | Classification | Neural Networks (NN) | MLP, LogR | MLP | Hold-out | Accuracy, Sensitivity, Specificity, F_1 score | NR |
| Valdeolivar-Hernandez et al [72] | | Prediction | NLP | Classification | Ensemble Methods (Bagging) | RF | Random Forest | K-fold-Cross-Validation | NR | NR |
| Wagay [73] | | Prediction | ML | Classification | NLP | NLP | NLP | NR | Accuracy, Precision, Sensitivity, Specificity, F1-Score | Tunning Parameter |
| Wakefield and Frasch [74] | | Prediction | ML | Classification | Ensemble Methods (Bagging) | RF, GB, LinR, XTR, AdaBoost, CatBoost, Stacking, Nested Stacking | Random Forest | K-fold-Cross-Validation | Accuracy, AUC | NR |
| Wang et al [78] | | Prediction | ML | Classification | Ensemble Methods (Bagging) | LogR, RF | Random Forest | K-fold-Cross-Validation | Accuracy, Precision, F_1 score, AUC | Tunning Parameter |
| Wang et al [76] | | Prediction | ML | Classification | Ensemble Methods (Bagging) | DT, KNN, RF, SVM | Random Forest | Hold-out | Accuracy, Sensitivity, Specificity, AUC | L1/L2 Regularization |
| Wang et al [75] | | Detection | ML | Classification | Classification | DT, LogR, Navie Bayes, RF, SVM, XGBoost | Support Vector Machine | K-fold-Cross-Validation | Sensitivity,Specificity, AUC | NR |
| Wang et al [77] | | Prediction | ML | Regression | Classification | SVM | Support Vector Machine | K-fold-Cross-Validation | Accuracy, AUC | L1/L2 Regularization |
| Xu et al [79] | | Detection | ML | Classification | Probabilistic Classification | Lasso, LogR | Logistic Regression | K-fold-Cross-Validation | Accuracy, Sensitivity, Specificity, AUC | Tunning Parameter ( Grid Search) |
| Xu and Sampson [80] | | Prediction | ML | Classification | Classification | SVM | Support Vector Machine | K-fold-Cross-validation | Accuracy, AUC | NR |
| Yu et al [81] | | Prediction | ML | Classification | Ensemble Methods (Boosting) | RF, NN, XGBoost | XGBoost | K-fold-Cross-Validation | AUC | Tunning Parameter ( Grid Search) |
| Zhang et al [82] | | Prediction | ML | Classification | Ensemble Methods (Bagging) | RF | Random Forest | Hold-out | Accuracy, Sensitivity, Specificity, PPV, NPV, AUC | NR |
| Zhang et al [83] | | Prediction | ML | Classification | Ensemble Methods (Bagging) | SVM, RF | Random Forest | K-fold-Cross-validation | AUC, Sensitivity, PPV | L1/L2 Regularization |
| Zhang et al [84] | | Prediction | ML | Classification | Probabilistic Classification | LogR | Logistic Regression | K-fold-Cross-validation | AUC, Sensitivity, Specificity, PPV, NPV | Tunning Parameter (Grid Search) |
| Zhu et al [85] | | Prediction | ML | Classification | Probabilistic Classification | DT, LogR, MLP, RF, XGBoost | Logistic Regression | K-fold-Cross-validation | Accuracy, Precision | Tunning Parameter |
| NR: Not reported | |  |  |  |  |  |  |  |  |  |
|  |  | | |  |  |  |  |  |  |  |
