# Supplementary material for: AI for Detecting and Predicting Postpartum Depression: Scoping Review
Source: J Med Internet Res. 2026 Jan 8;28:e77376. doi: 10.2196/77376 (PMC12782538; doi:10.2196/77376)
Supplement: Multimedia Appendix 8 [file jmir-v28-e77376-s008.docx]

Multimedia Appendix 8. Characteristics of ML performances (accuracy, F_1_-score, AUC).

**Characteristics 1: Characteristics of ML Performances (Accuracy)**

| ID | Author | Year | Author & Year | Algorithm | Accuracy % |
| --- | --- | --- | --- | --- | --- |
| 1 | Ajay et al [24] | 2024 | Ajay 2024 | Random Forest (RF) | 96.00 |
|  |  |  | Ajay 2024 | XGBoost | 92.00 |
|  |  |  | Ajay 2024 | Decision Tree (DT) | 89.00 |
| 2 | Sharma et al [21] | 2024 | Sharma 2024 | Logistic Regression | 77.26 |
|  |  |  | Sharma 2024 | Decision Tree (DT) | 90.64 |
|  |  |  | Sharma 2024 | Random Forest (RF) | 91.30 |
|  |  |  | Sharma 2024 | Support Vector Machine (SVM) | 91.52 |
|  |  |  | Sharma 2024 | K-nearest neighbor (KNN) | 92.64 |
|  |  |  | Sharma 2024 | XGBoost | 93.08 |
|  |  |  | Sharma 2024 | Voting Classifier (dt, knn, svc, rf) | 93.65 |
|  |  |  | Sharma 2024 | Neural Network (NN) | 93.75 |
|  |  |  | Sharma 2024 | Multi-layered Stacking (proposed model) | 94.31 |
| 3 | Andersson et al [22] | 2021 | Andersson 2021 | Ridge Regression | 67 |
|  |  |  | Andersson 2021 | LASSO Regression | 70 |
|  |  |  | Andersson 2021 | Gradient Boosted Machines | 68 |
|  |  |  | Andersson 2021 | Distributed Random Forests | 71 |
|  |  |  | Andersson 2021 | Extreme Randomized Forest | 73 |
|  |  |  | Andersson 2021 | Naïve Bayes (NB) | 69 |
|  |  |  | Andersson 2021 | Stacked Ensemble | 65 |
| 4 | Betts et al [23] | 2020 | Betts 2020 | NR | NR |
| 5 | Cai et al [25] | 2019 | Cai 2019 | NR | NR |
| 6 | Carneiro et al [26] | 2020 | Carneiro 2020 | Naïve Bayes (NB) | 86.36 |
| 7 | Chen et al [27] | 2018 | Chen 2018 | LSTM Neural Network | NR |
| 8 | Fanos et al [28] | 2023 | Fanos 2023 | NR | NR |
| 9 | Fatima et al [29] | 2019 | Fatima 2019 | Support Vector Machine (SVM) | 76.86 |
|  |  |  | Fatima 2019 | Multilayer Perceptron (MLP) | 86.91 |
|  |  |  | Fatima 2019 | Logistic Regression | 79.76 |
| 10 | Fazraningtyas et al [30] | 2025 | Fazraningtyas 2025 | C4.5 DT | 68.77 |
|  |  |  | Fazraningtyas 2025 | XGBoost | 87.57 |
|  |  |  | Fazraningtyas 2025 | C4.5 DT + FS | 78.27 |
| 11 | Gabrieli et al [31] | 2020 | Gabrieli 2020 | Google AutoML Tables | 89.5 |
| 12 | Gopalakrishnan et al [34] | 2022 | Gopalakrishnan 2022 | Extremely Randomized Trees | 73 |
|  |  |  | Gopalakrishnan 2022 | LASSO Regression | 70 |
|  |  |  | Gopalakrishnan 2022 | Gradient Boosted Machines | 68 |
|  |  |  | Gopalakrishnan 2022 | Naïve Bayes (NB) | 69 |
|  |  |  | Gopalakrishnan 2022 | Ridge Regression | 67 |
| 13 | Gopalakrishnan et al [33] | 2023 | Gopalakrishnan 2023 | NR | NR |
|  |  |  | Gopalakrishnan 2023 | NR | NR |
| 14 | Gopalakrishnan et al [32] | 2023 | Gopalakrishnan 2023 | Support Vector Machine (SVM) | 89 |
|  |  |  | Gopalakrishnan 2023 | Random Forest (RF) | 82 |
| 15 | Gupta et al [35] | 2024 | Gupta 2024 | LightGBM | 98.37 |
|  |  |  | Gupta 2024 | CatBoost | 99.46 |
| 16 | Horgen [36] | 2022 | Horgen 2022 | NR | NR |
| 17 | Hurwitz et al [37] | 2024 | Hurwitz 2024 | NR | NR |
| 18 | Jimenez-Serrano et al [38] | 2015 | Jimenez-Serrano 2015 | Naïve Bayes (NB) | 0.73 (0.68, 0.78) |
|  |  |  | Jimenez-Serrano 2015 | Logistic regression | 74 |
|  |  |  | Jimenez-Serrano 2015 | Support Vector Machine (SVM) | 73 |
|  |  |  | Jimenez-Serrano 2015 | ANN | 79 |
| 19 | Krishnamurti et al [39] | 2022 | Krishnamurti 2022 | NR | NR |
| 20 | Lilhore et al [41] | 2024 | Lilhore 2024 | CNN (AlexNet) with Transfer Learning combined with an improved Bi-LSTM | 98.188 |
|  |  |  | Lilhore 2024 | CNN Model | 90.501 |
|  |  |  | Lilhore 2024 | VGG-16 | 92.348 |
|  |  |  | Lilhore 2024 | CNN-LSTM | 93.741 |
| 21 | Lilhore et al [40] | 2024 | Lilhore 2024 | CNN-BLSTM with a TL-based model | 100 |
|  |  |  | Lilhore 2024 | XGBoost | 100 |
|  |  |  | Lilhore 2024 | CatBoost | 99 |
|  |  |  | Lilhore 2024 | LightGBM | 97 |
|  |  |  | Lilhore d 2024 | Random Forest (RF) | 88 |
|  |  |  | Lilhore d 2024 | Decision Tree (DT) | 87 |
|  |  |  | Lilhore d 2024 | AdaBoost | 85 |
| 22 | Liu et al [43] | 2024 | Liu 2024 | NR | NR |
| 23 | Liu et al [42] | 2023 | Liu 2023 | Logistic Regression | 67.2 |
|  |  |  | Liu 2023 | Support Vector Machine (SVM) | 71.1 |
|  |  |  | Liu 2023 | Random Forest Classifier | 59 |
|  |  |  | Liu 2023 | XGBoost | 67.6 |
|  |  |  | Liu 2023 | LightGBM | 70.2 |
|  |  |  | Liu 2023 | Multilayer Perceptron (MLP) | 68 |
| 24 | Lyall et al [44] | 2023 | Lyall 2023 | Ridge Regression | 67.76 |
|  |  |  | Lyall 2023 | LASSO Regression | 68.16 |
| 25 | Marshad et al [45] | 2024 | Marshad 2024 | AdaBoost | 76.5 |
|  |  |  | Marshad 2024 | XGBoost | 99.15 |
|  |  |  | Marshad 2024 | Gradient Boosting (GB) | 89.74 |
|  |  |  | Marshad 2024 | LightGBM | 96.15 |
|  |  |  | Marshad 2024 | CatBoost | 94.02 |
| 26 | Matsumura et al [46] | 2024 | Matsumura 2024 | Decision Tree (DT) | 77.2 |
| 27 | Matsuo et al [47] | 2022 | Matsuo 2022 | NR | NR |
| 28 | Mazumder and Baruah [48] | 2021 | Mazumder and Baruah 2021 | AdaBoost + Bagging | 94 |
| 29 | Moreira et al [49] | 2019 | Moreira 2019 | Ensemble (Bagged Trees) | 95.8 |
| 30 | Mustafa [50] | 2023 | Mustafa 2023 | Naïve Bayes (NB) | 75 |
|  |  |  | Mustafa 2023 | Logistic regression | 75 |
|  |  |  | Mustafa 2023 | Support Vector Machine (SVM) | 77 |
|  |  |  | Mustafa 2023 | ANN | 78 |
| 31 | Myneni et al [51] | 2024 | Myneni 2024 | NR | NR |
| 32 | Nasim et al [52] | 2024 | Nasim 2024 | Stochastic Gradient Descent (SGD) | 75 |
|  |  |  | Nasim 2024 | Linear Regression (LiR) | 78 |
|  |  |  | Nasim 2024 | Random Forest (RF) | 86 |
|  |  |  | Nasim 2024 | Bayesian Ridge (BR) | 79 |
|  |  |  | Nasim 2024 | Support Vector Machine (SVM) | 78 |
|  |  |  | Nasim 2024 | K-Neighbors Classifier (KNC) | 89 |
|  |  |  | Nasim 2024 | Multilayer Perceptron (MLP) | 92 |
|  |  |  | Nasim 2024 | Logistic regression | 79 |
|  |  |  | Nasim 2024 | Gaussian Naive Bayes (GNB) | 77 |
|  |  |  | Nasim 2024 | Gradient Boosting Classifier (GBC) | 92 |
|  |  |  | Nasim 2024 | Decision Tree (DT) | 88 |
| 33 | Natarajan et al[53] | 2017 | Natarajan 2017 | NR | NR |
| 34 | Osubor and Egwali [54] | 2018 | Osubor and Egwali 2018 | ANFIS | 97 |
|  |  |  | Osubor and Egwali 2018 | ANN | 92 |
| 35 | Park et al [55] | 2021 | Park 2021 | Logistic regression | 73 |
|  |  |  | Park 2021 | Random Forest (RF) | 73 |
|  |  |  | Park 2021 | XGBoost | 73 |
|  |  |  | Park 2021 | Logistic regression | 78 |
|  |  |  | Park 2021 | Random Forest (RF) | 78 |
|  |  |  | Park 2021 | XGBoost | 78 |
| 36 | Paul et al [56] | 2023 | Paul 2023 | Random Forest (RF) | 71.19 |
|  |  |  | Paul 2023 | K-nearest neighbor (KNN) | 61.5 |
|  |  |  | Paul 2023 | Logistic regression | 69.57 |
|  |  |  | Paul 2023 | AdaBoost | 68.98 |
|  |  |  | Paul 2023 | Support Vector Machine (SVM) | 74.41 |
|  |  |  | Paul 2023 | Multilayer Perceptron (MLP) | 70.64 |
|  |  |  | Paul 2023 | TabNet | 70.72 |
|  |  |  | Paul 2023 | Recursive Partitioning (RPART) | 70.59 |
|  |  |  | Paul 2023 | C4.5 DT | 70.55 |
|  |  |  | Paul 2023 | Gradient Boosted Machines | 70.92 |
| 37 | Payne et al [57] | 2020 | Payne 2020 | NR | NR |
| 38 | Prabhashwaree and Wagarachchi [58] | 2022 | Prabhashwaree and Wagarachchi 2022 | FFANN- (25 epoch) | 95 |
|  |  |  | Prabhashwaree and Wagarachchi 2022 | Support Vector Machine (SVM) | 93.57 |
|  |  |  | Prabhashwaree and Wagarachchi 2022 | Random Forest (RF) | 92.14 |
| 39 | Prabhashwaree and Wagarachchi [59] | 2022 | Prabhashwaree and Wagarachchi2022 | FFANN- (25 epoch) | 97.08 |
|  |  |  | Prabhashwaree and Wagarachchi 2022 | Support Vector Machine (SVM) | 94.89 |
|  |  |  | Prabhashwaree and Wagarachchi 2022 | Random Forest (RF) | 93.43 |
| 40 | Qasrawi et al [60] | 2022 | Qasrawi 2022 | K-nearest neighbor (KNN) | 78.7 |
|  |  |  | Qasrawi 2022 | Decision Tree (DT) | 79.4 |
|  |  |  | Qasrawi 2022 | Support Vector Machine (SVM) | 74.7 |
|  |  |  | Qasrawi 2022 | Random Forest (RF) | 81.3 |
|  |  |  | Qasrawi 2022 | Neural Network (NN) | 80.1 |
|  |  |  | Qasrawi 2022 | Naïve Bayes (NB) | 81.3 |
|  |  |  | Qasrawi 2022 | Gradient Boosting (GB) | 82.9 |
| 41 | Raisa et al [61] | 2022 | Raisa 2022 | Random Forest (RF) | 89 |
|  |  |  | Raisa 2022 | XGBoost | 86 |
|  |  |  | Raisa 2022 | Support Vector Machine (SVM) | 84 |
|  |  |  | Raisa 2022 | Gradient Boosting (GB) | 84 |
|  |  |  | Raisa 2022 | Logistic regression | 82 |
| 42 | Reps et al [62] | 2022 | Reps 2022 | NR |  |
| 43 | Shen et al [63] | 2023 | Shen 2023 | Naïve Bayes (NB) | 68.04 |
|  |  |  | Shen 2023 | K-nearest neighbor (KNN) | 81.01 |
|  |  |  | Shen 2023 | Support Vector Machine (SVM) | 78.03 |
|  |  |  | Shen 2023 | Random Forest (RF) | 69.01 |
|  |  |  | Shen 2023 | Logistic regression | 81.05 |
|  |  |  | Shen 2023 | Decision Tree (DT) | 83.05 |
|  |  |  | Shen 2023 | Deep Reinforcement Learning (RL)+Differential Evolution (DE)(Proposed model) | 89.07 |
|  |  |  | Shen 2023 | Proposed+random weights | 81.04 |
|  |  |  | Shen 2023 | Proposed+random weights+RL | 86.15 |
| 44 | Shin et al [64] | 2020 | Shin 2020 | Random Forest (RF) | 79.1 |
|  |  |  | Shin 2020 | Support Vector Machine (SVM) | 78.9 |
|  |  |  | Shin 2020 | Gradient Boosted Machines | 78.1 |
|  |  |  | Shin 2020 | AdaBoost | 77.8 |
|  |  |  | Shin 2020 | Naïve Bayes (NB) | 67.5 |
|  |  |  | Shin 2020 | Recursive partitioning (RPART) | 73.1 |
|  |  |  | Shin 2020 | K-nearest neighbor (KNN) | 64.1 |
|  |  |  | Shin 2020 | Logistic regression | 65.5 |
|  |  |  | Shin 2020 | Neural Network (NN) | 65 |
| 45 | Shivaprasad et al [65] | 2024 | Shivaprasad 2024 | Random Forest (RF) | 85 |
|  |  |  | Shivaprasad 2024 | Logistic regression | 68 |
|  |  |  | Shivaprasad 2024 | Decision Tree (DT) | 85 |
|  |  |  | Shivaprasad 2024 | K-nearest neighbor (KNN) | 97 |
|  |  |  | Shivaprasad 2024 | AdaBoost | 66 |
|  |  |  | Shivaprasad 2024 | CatBoost | 77 |
|  |  |  | Shivaprasad 2024 | LightGBM | 96 |
|  |  |  | Shivaprasad 2024 | XGBoost | 96 |
|  |  |  | Shivaprasad 2024 | Stack | 97 |
| 46 | Srivatsav and Nanthini [66] | 2024 | Srivatsav and Nanthini 2024 | LSTM-CNN | 77.26 |
|  |  |  | Srivatsav and Nanthini 2024 | Logistic Regression | 72.36 |
| 47 | Suganthi and Geetha [67] | 2024 | Suganthi and Geetha 2024 | Multilayer Perceptron (MLP) | 91.02 |
|  |  |  | Suganthi and Geetha 2024 | Extremely Randomized Trees (XRT) | 92.35 |
|  |  |  | Suganthi and Geetha 2024 | Decision Tree (DT) | 93.57 |
|  |  |  | Suganthi and Geetha 2024 | Logistic Regression | 94.29 |
|  |  |  | Suganthi and Geetha 2024 | XGBoost | 95.01 |
|  |  |  | Suganthi and Geetha 2024 | Osprey Parameter Optimized MLP (OPOMLP) | 96.12 |
| 48 | Susič et al [68] | 2023 | Susič 2023 | Support Vector Classifier | 64 |
| 49 | Tang et al [69] | 2024 | Tang 2024 | Mutual Learning-based Artificial Bee Colony (ML-ABC) with Proximal Policy Optimization (PPO) | 91 |
| 50 | Tortajada et al [70] | 2009 | Tortajada 2009 | Multilayer Perceptron (MLP) (Neural Net) | 81 |
|  |  |  | Tortajada 2009 | Multilayer Perceptron (MLP) (Neural Net) | 84 |
|  |  |  | Tortajada 2009 | Multilayer Perceptron (MLP) (Neural Net) | 78 |
|  |  |  | Tortajada 2009 | Multilayer Perceptron (MLP) (Neural Net) | 84 |
| 51 | Valavani et al [71] | 2022 | Valavani 2022 | Random Forest (RF) | 75 |
| 52 | Valdeolivar-Hernandez et al [72] | 2022 | Valdeolivar-Hernandez 2022 | NR | NR |
| 53 | Wagay [73] | 2023 | Wagay 2023 | Random Forest (RF) | 82.3 |
|  |  |  | Wagay 2023 | ExtraTree Classifier | 98.13 |
|  |  |  | Wagay 2023 | Light Gradient Boosting (LGB) | 97.8 |
|  |  |  | Wagay 2023 | CatBoost | 98.4 |
|  |  |  | Wagay 2023 | XGBoost | 97.73 |
|  |  |  | Wagay 2023 | AdaBoost | 82.7 |
|  |  |  | Wagay 2023 | Hard Voting Ensemble Classifier(NN) | 98.27 |
|  |  |  | Wagay 2023 | Soft Voting Ensemble Classifier(NN) | 95.14 |
|  |  |  | Wagay 2023 | Weighted Majority Voting(NN) | 97.71 |
|  |  |  | Wagay 2023 | Stacking | 97.8 |
|  |  |  | Wagay 2023 | Nested Stacking | 99.84 |
| 54 | Wakefield and Frasch [74] | 2023 | Wakefield 2023 | NR | NR |
| 55 | Wang et al [78] | 2024 | Wang 2024 | NR | NR |
| 56 | Wang et al [76] | 2019 | Wang 2019 | NR | NR |
| 57 | Wang et al [75] | 2017 | Wang-TRAJECTORY 2017 | K-nearest neighbor (KNN) | 72.1 |
|  |  |  | Wang-TRAJECTORY 2018 | Decision Tree (DT) | 71.2 |
|  |  |  | Wang-TRAJECTORY 2018 | Random Forest (RF) | 78.2 |
|  |  |  | Wang-TRAJECTORY 2018 | Support Vector Machine (SVM) | 77.1 |
| 58 | Wang et al [77] | 2025 | Wang-PLASMA 2025 | NR | NR |
| 59 | Xu et al [79] | 2023 | Xu 2023 | Support Vector Machine (SVM) | 76 |
| 60 | Xu and Sampson [80] | 2023 | Xu 2023 | NR | NR |
| 61 | Yu et al [81] | 2022 | Yu 2022 | NR | NR |
| 62 | Zhang et al [82] | 2021 | Zhang 2020 | NR | NR |
| 63 | Zhang et al [83] | 2024 | Zhang 2024 | NR | NR |
| 64 | Zhang et al [84] | 2020 | Zhang 2020 | NR | NR |
| 65 | Zhu et al [85] | 2021 | Zhu 2021 | XGBoost | 92.53 |
|  |  |  | Zhu 2021 | Logistic Regression | 68.57 |
|  |  |  | Zhu 2021 | Random Forest (RF) | 78.09 |
|  |  |  | Zhu 2021 | Support Vector Machine (SVM) | 87.63 |

**Characteristics 2:Characteristics of ML Performances (F1_Score)**

| ID | Author | Year | Author & Year | Algorithm | F1 score (%) |
| --- | --- | --- | --- | --- | --- |
| 1 | Ajay et al [24] | 2024 | Ajay 2024 | Random Forest | 85 |
|  |  |  | Ajay 2024 | XGBoost | 66 |
|  |  |  | Ajay 2024 | Decision Tree (DT) | 66 |
| 2 | Sharma et al [21] | 2024 | Sharma 2024 | Logistic Regression | 84.95 |
|  |  |  | Sharma 2024 | Decision Tree (DT) | 93 |
|  |  |  | Sharma 2024 | Random Forest | 93.66 |
|  |  |  | Sharma 2024 | Support Vector Machine (SVM) | 93.56 |
|  |  |  | Sharma 2024 | K-nearest neighbor (KNN) | 94.58 |
|  |  |  | Sharma 2024 | XGBoost | 94.77 |
|  |  |  | Sharma 2024 | Voting Classifier (dt, knn, svc, rf) | 95.26 |
|  |  |  | Sharma 2024 | Neural Network (NN) | 95.22 |
|  |  |  | Sharma 2024 | Multi-layered Stacking (proposed model) | 95.84 |
| 3 | Andersson et al [22] | 2021 | Andersson 2021 | NR | NR |
| 4 | Betts et al [23] | 2020 | Betts 2020 | NR | NR |
| 5 | Cai et al [25] | 2019 | Cai 2019 | NR | NR |
| 6 | Carneiro et al [26] | 2020 | Carneiro 2020 | Naïve Bayes (NB) | 88.66 |
| 7 | Chen et al [27] | 2018 | Chen 2018 | LSTM Neural Network | NR |
| 8 | Fanos et al [28] | 2023 | Fanos 2023 | NR | NR |
| 9 | Fatima et al [29] | 2019 | Fatima 2019 | NR | NR |
| 10 | Fazraningtyas et al [30] | 2025 | Fazraningtyas 2025 | NR | NR |
| 11 | Gabrieli et al [31] | 2020 | Gabrieli 2020 | NR | NR |
| 12 | Gopalakrishnan et al [34] | 2022 | Gopalakrishnan 2022 | NR | NR |
| 13 | Gopalakrishnan a et al [33] | 2023 | Gopalakrishnan a 2023 | NR | NR |
|  |  |  | Gopalakrishnan a 2023 | NR | NR |
|  |  |  | Gopalakrishnan a 2023 | NR | NR |
| 14 | Gopalakrishnan b et al [32] | 2023 | Gopalakrishnan 2023 | Support Vector Machine (SVM) | 91 |
|  |  |  | Gopalakrishnan 2023 | Random Forest | 80 |
| 15 | Gupta et al [35] | 2024 | Gupta 2024 | LightGBM | 98.56 |
|  |  |  | Gupta 2024 | CatBoost | 99.06 |
| 16 | Horgen et al [36] | 2022 | Horgen 2022 | NR | NR |
| 17 | Hurwitz et al [37] | 2024 | Hurwitz 2024 | Random Forest | 81 |
|  |  |  | Hurwitz 2024 | Binomial classifier | 52 |
| 18 | Jimenez-Serrano et al [38] | 2015 | Jimenez-Serrano 2015 | NR | NR |
| 19 | Krishnamurti et al [39] | 2022 | Krishnamurti 2022 | NR | NR |
| 20 | Lilhore et al [41] | 2024 | Lilhore 2024 | CNN (AlexNet) with Transfer Learning combined with an improved Bi-LSTM | 98.46 |
|  |  |  | Lilhore 2024 | CNN Model | 91.108 |
|  |  |  | Lilhore 2024 | VGG-16 | 92.478 |
|  |  |  | Lilhore 2024 | CNN-LSTM | 93.625 |
| 21 | Lilhore et al [40] | 2024 | Lilhore 2024 | CNN-BLSTM with a TL-based model | 100 |
|  |  |  | Lilhore 2024 | XGBoost | 100 |
|  |  |  | Lilhore 2024 | CatBoost | 90 |
|  |  |  | Lilhore 2024 | LightGBM | 98 |
|  |  |  | Lilhore 2024 | Random Forest | 91 |
|  |  |  | Lilhore 2024 | Decision Tree (DT) | 91 |
|  |  |  | Lilhore 2024 | AdaBoost | 89 |
| 22 | Liu et al [43] | 2024 | Liu 2024 | NR | NR |
| 23 | Liu et al [42] | 2023 | Liu 2023 | Logistic Regression | 38.2 |
|  |  |  | Liu 2023 | Support Vector Machine (SVM) | 42.2 |
|  |  |  | Liu 2023 | Random Forest Classifier | 39.3 |
|  |  |  | Liu 2023 | Extreme Gradient Boosting | 45 |
|  |  |  | Liu 2023 | LightGBM | 41.6 |
|  |  |  | Liu 2023 | Multilayer Perceptron (MLP) | 40.6 |
| 24 | Lyall et al [44] | 2023 | Lyall 2023 | NR | NR |
| 25 | Marshad et al [45] | 2024 | Marshad 2024 | AdaBoost | 72.64 |
|  |  |  | Marshad 2024 | XGBoost | 99 |
|  |  |  | Marshad 2024 | Gradient Boosting (GB) | 88.11 |
|  |  |  | Marshad 2024 | LightGBM | 95.48 |
|  |  |  | Marshad 2024 | CatBoost | 92.86 |
| 26 | Matsumura et al [46] | 2024 | Matsumura 2024 | NR | NR |
| 27 | Matsuo et al [47] | 2022 | Matsuo 2022 | NR | NR |
| 28 | Mazumder and Baruah [48] | 2021 | Mazumder and Baruah 2021 | Random forest | 94.8 |
| 29 | Moreira et al [49] | 2019 | Moreira 2019 | NR | NR |
| 30 | Mustafa et al [50] | 2023 | Mustafa 2023 | NR | NR |
| 31 | Myneni et a. [51] | 2024 | Myneni 2024 | BERT model | 96 |
| 32 | Nasim et al [52] | 2024 | Nasim 2024 | Stochastic Gradient Descent (SGD) | 74 |
|  |  |  | Nasim 2024 | Linear Regression (LiR) | 76 |
|  |  |  | Nasim 2024 | Random forest | 85 |
|  |  |  | Nasim 2024 | Bayesian Ridge (BR) | 77 |
|  |  |  | Nasim 2024 | Support Vector Machine (SVM) | 76 |
|  |  |  | Nasim 2024 | K-Neighbors Classifier (KNC) | 88 |
|  |  |  | Nasim 2024 | Multilayer Perceptron (MLP) | 91 |
|  |  |  | Nasim 2024 | Logistic regression | 77 |
|  |  |  | Nasim 2024 | Gaussian Naive Bayes (GNB) | 75 |
|  |  |  | Nasim 2024 | Gradient Boosting Classifier (GBC) | 92 |
|  |  |  | Nasim 2024 | Decision Tree (DT) | 88 |
| 33 | Natarajan et al [53] | 2017 | Natarajan 2017 | NR | NR |
| 34 | Osubor and Egwali [54] | 2018 | Osubor and Egwali 2018 | NR | NR |
| 35 | Park et al [55] | 2021 | Park 2021 | NR | NR |
| 36 | Paul et al [56] | 2023 | Paul 2023 | Random forest | 71 |
|  |  |  | Paul 2023 | K-nearest neighbor (KNN) | 57 |
|  |  |  | Paul 2023 | Logistic regression | 71.08 |
|  |  |  | Paul 2023 | AdaBoost | 69 |
|  |  |  | Paul 2023 | Support Vector Machine (SVM) | 72 |
|  |  |  | Paul 2023 | Multilayer Perceptron (MLP) | 71 |
|  |  |  | Paul 2023 | TabNet | 71.72 |
|  |  |  | Paul 2023 | Recursive Partitioning (RPART) | 71 |
|  |  |  | Paul 2023 | C4.5 DT | 71 |
|  |  |  | Paul 2023 | Gradient Boosted Machines | 71 |
| 37 | Payne et al [57] | 2020 | Payne 2020 | NR | NR |
| 38 | Prabhashwaree and Wagarachchi [58] | 2022 | Prabhashwaree and Wagarachchi 2022 | FFANN- (25 epoch) | 93 |
|  |  |  | Prabhashwaree and Wagarachchi 2022 | Support Vector Machine (SVM) | 94 |
|  |  |  | Prabhashwaree and Wagarachchi 2022 | Random forest | 95 |
| 39 | Prabhashwaree and Wagarachchi [59] | 2022 | Prabhashwaree and Wagarachchi 2022 | FFANN- (25 epoch) | 93 |
|  |  |  | Prabhashwaree and Wagarachchi 2022 | Support Vector Machine (SVM) | 90 |
|  |  |  | Prabhashwaree and Wagarachchi 2022 | Random Forest | 88 |
| 40 | Qasrawi et al [60] | 2022 | Qasrawi 2022 | K-nearest neighbor (KNN) | 76.7 |
|  |  |  | Qasrawi 2022 | Decision Tree (DT) | 78.9 |
|  |  |  | Qasrawi 2022 | Support Vector Machine (SVM) | 74.4 |
|  |  |  | Qasrawi 2022 | Random Forest (RF) | 81.4 |
|  |  |  | Qasrawi 2022 | Neural Network (NN) | 80 |
|  |  |  | Qasrawi 2022 | Naïve Bayes (NB) | 82.4 |
|  |  |  | Qasrawi 2022 | Gradient Boosting (GB) | 82.9 |
| 41 | Raisa et al [61] | 2022 | Raisa 2022 | Random Forest (RF) | 89 |
|  |  |  | Raisa 2022 | XGBoost | 85 |
|  |  |  | Raisa 2022 | Support Vector Machine (SVM) | 84 |
|  |  |  | Raisa 2022 | Gradient Boosting (GB) | 82 |
|  |  |  | Raisa 2022 | Logistic regression | 82 |
| 42 | Reps et al [62] | 2022 | Reps 2022 | NR |  |
| 43 | Shen et al [63] | 2023 | Shen 2023 | Naïve Bayes (NB) | 56.01 |
|  |  |  | Shen 2023 | K-nearest neighbor (KNN) | 74.71 |
|  |  |  | Shen 2023 | Support Vector Machine (SVM) | 67.02 |
|  |  |  | Shen 2023 | Random Forest (RF) | 55.02 |
|  |  |  | Shen 2023 | Logistic regression | 73.02 |
|  |  |  | Shen 2023 | Decision Tree (DT) | 79.02 |
|  |  |  | Shen 2023 | Deep Reinforcement Learning (RL)+Differential Evolution (DE)(Proposed model) | 88.44 |
|  |  |  | Shen 2023 | Proposed+random weights | 79.18 |
|  |  |  | Shen 2023 | Proposed+random weights+RL | 85.06 |
| 44 | Shin et al [64] | 2020 | Shin 2020 | Random Forest (RF) | 77.6 |
|  |  |  | Shin 2020 | Support Vector Machine (SVM) | 78.9 |
|  |  |  | Shin 2020 | Gradient boosting machine | 76 |
|  |  |  | Shin 2020 | AdaBoost | 76.5 |
|  |  |  | Shin 2020 | Naïve Bayes (NB) | 64.7 |
|  |  |  | Shin 2020 | Recursive partitioning (RPART) | 70.8 |
|  |  |  | Shin 2020 | K-nearest neighbor (KNN) | 71.5 |
|  |  |  | Shin 2020 | Logistic regression | 64.6 |
|  |  |  | Shin 2020 | Neural Network (NN) | 65.1 |
| 45 | Shivaprasad et al [65] | 2024 | Shivaprasad 2024 | Random Forest (RF) | 86 |
|  |  |  | Shivaprasad 2024 | Logistic regression | 64 |
|  |  |  | Shivaprasad 2024 | Decision Tree (DT) | 84 |
|  |  |  | Shivaprasad 2024 | K-nearest neighbor (KNN) | 97 |
|  |  |  | Shivaprasad 2024 | AdaBoost | 58 |
|  |  |  | Shivaprasad 2024 | CatBoost | 72 |
|  |  |  | Shivaprasad 2024 | LightGBM | 95 |
|  |  |  | Shivaprasad 2024 | XGBoost | 96 |
|  |  |  | Shivaprasad 2024 | Stack | 97 |
| 46 | Srivatsav and Nanthini [66] | 2024 | Srivatsav and Nanthini 2024 | NR |  |
| 47 | Suganthi and Geetha [67] | 2024 | Suganthi and Geetha 2024 | Multilayer Perceptron (MLP) | 91.68 |
|  |  |  | Suganthi and Geetha 2024 | Extremely Randomized Trees (XRT) | 92.31 |
|  |  |  | Suganthi and Geetha 2024 | Decision Tree (DT) | 93.67 |
|  |  |  | Suganthi and Geetha 2024 | Logistic Regression | 94.13 |
|  |  |  | Suganthi and Geetha 2024 | XGBoost | 95.52 |
|  |  |  | Suganthi and Geetha 2024 | Osprey Parameter Optimized MLP (OPOMLP) | 96.38 |
| 48 | Susič et al [68] | 2023 | Susič 2023 | NR | NR |
| 49 | Tang et al [69] | 2024 | Tang 2024 | Mutual Learning-based Artificial Bee Colony (ML-ABC) with Proximal Policy Optimization (PPO) | 88 |
| 50 | Tortajada et al [70] | 2009 | Tortajada 2009 | NR | NR |
| 51 | Valavani et al [71] | 2022 | Valavani 2022 | Random Forest (RF) | 80 |
| 52 | Valdeolivar-Hernandez et al [72] | 2023 | Valdeolivar-Hernandez 2023 | NR | NR |
| 53 | Wagay [73] | 2023 | Wagay 2023 | Random Forest (RF) | 87.22 |
|  |  |  | Wagay 2023 | ExtraTree Classifier | 98.58 |
|  |  |  | Wagay 2023 | Light Gradient Boosting (LGB) | 98.31 |
|  |  |  | Wagay 2023 | CatBoost | 98.78 |
|  |  |  | Wagay 2023 | XGBoost | 98.27 |
|  |  |  | Wagay 2023 | AdaBoost | 87.11 |
|  |  |  | Wagay 2023 | Hard Voting Ensemble Classifier(NN) | 98.42 |
|  |  |  | Wagay 2023 | Soft Voting Ensemble Classifier(NN) | 98.27 |
|  |  |  | Wagay 2023 | Weighted Majority Voting(NN) | 98.38 |
|  |  |  | Wagay 2023 | Stacking | 98.43 |
|  |  |  | Wagay 2023 | Nested Stacking | 99.21 |
| 54 | Wakefield and Frasch [74] | 2023 | Wakefield 2023 | NR | NR |
| 55 | Wang et al [78] | 2018 | Wang 2024 | SVM with SFFS-selected features | 69 |
| 56 | Wang et al [76] | 2019 | Wang 2019 | NR | NR |
| 57 | Wang et al [75] | 2017 | Wang-TRAJECTORY 2017 | K-nearest neighbor (KNN) | 67.2 |
|  |  |  | Wang-TRAJECTORY 2017 | Decision Tree (DT) | 71.7 |
|  |  |  | Wang-TRAJECTORY 2017 | Random Forest (RF) | 76.9 |
|  |  |  | Wang-TRAJECTORY 2017 | Support Vector Machine (SVM) | 75.2 |
| 58 | Wang et al [77] | 2025 | Wang-PLASMA 2025 | NR | NR |
| 59 | Xu et al [79] | 2023 | Xu 2023 | NR | NR |
| 60 | Xu and Sampson [80] | 2023 | Xu 2023 | NR | NR |
| 61 | Yu et al [81] | 2022 | Yu 2022 | NR | NR |
| 62 | Zhang et al [82] | 2021 | Zhang 2020 | NR | NR |
| 63 | Zhang et al [83] | 2024 | Zhang 2024 | NR | NR |
| 64 | Zhang et al [84] | 2020 | Zhang 2020 | NR | NR |
| 65 | Zhu et al [85] | 2021 | Zhu 2021 | NR | NR |

**Characteristics 3: Characteristics of ML Performances (AUC)**

| ID | Author | Year | Author & Year | Algorithm | AUC (%) |
| --- | --- | --- | --- | --- | --- |
| 1 | Ajay et al [24] | 2024 | Ajay 2024 | NR | NR |
| 2 | Sharma et al [21] | 2024 | Sharma 2024 | NR | NR |
| 3 | Andersson et al [22] | 2021 | Andersson 2021 | Ridge Regression | 79 |
|  |  |  | Andersson 2021 | LASSO Regression | 80 |
|  |  |  | Andersson 2021 | Gradient Boosted Machines | 73 |
|  |  |  | Andersson 2021 | Distributed Random Forests | 80 |
|  |  |  | Andersson 2021 | Extreme Randomized Forest | 81 |
|  |  |  | Andersson 2021 | Naïve Bayes (NB) | 78 |
|  |  |  | Andersson 2021 | Stacked Ensemble | 80 |
| 4 | Betts et al [23] | 2020 | Betts 2020 | Boosted Trees(XGBoost) | 80 |
|  |  |  | Betts 2020 | Elastic Net | 78 |
|  |  |  | Betts 2020 | Logistic Regression | 76 |
| 5 | Cai et al [25] | 2019 | Cai 2019 | C4.5 Decision Tree classifiers | 85.2 |
|  |  |  | Cai 2019 | Random Forest | 95.8 |
| 6 | Carneiro et al [26] | 2020 | Carneiro 2020 | NR | NR |
| 7 | Chen et al [27] | 2018 | Chen 2018 | LSTM Neural Network | NR |
| 8 | Fanos et al [28] | 2023 | Fanos 2023 | NR | NR |
| 9 | Fatima et al [29] | 2019 | Fatima 2019 | NR | NR |
| 10 | Fazraningtyas et al [30] | 2025 | Fazraningtyas 2025 | NR | NR |
| 11 | Gabrieli et al [31] | 2020 | Gabrieli 2020 | Google AutoML Tables | 96.9 |
| 12 | Gopalakrishnan et al [34] | 2022 | Gopalakrishnan 2022 | Extremely Randomized Trees | 81 |
|  |  |  | Gopalakrishnan 2022 | LASSO Regression | 80 |
|  |  |  | Gopalakrishnan 2022 | Gradient Boosted Machines | 73 |
|  |  |  | Gopalakrishnan 2022 | Naïve Bayes (NB) | 78 |
|  |  |  | Gopalakrishnan 2022 | Ridge Regression | 79 |
| 13 | Gopalakrishnan et al [33] | 2023 | Gopalakrishnan2023 | NR | NR |
| 14 | Gopalakrishnan et al [32] | 2023 | Gopalakrishnan 2023 | NR | NR |
| 15 | Gupta et al [35] | 2024 | Gupta 2024 | NR | NR |
| 16 | Horgen et al [36] | 2022 | Horgen 2022 | NR | NR |
| 17 | Hurwitz et al [37] | 2024 | Hurwitz 2024 | Random Forest | 85 |
|  |  |  | Hurwitz 2024 | Generalized Linear Models (GLM) | 82 |
|  |  |  | Hurwitz 2024 | Support Vector Machine (SVM) | 75 |
|  |  |  | Hurwitz 2024 | K-nearest neighbor (KNN) | 74 |
| 18 | Jimenez-Serrano et al [38] | 2015 | Jimenez-Serrano 2015 | Naïve Bayes (NB) | 75 |
|  |  |  | Jimenez-Serrano 2015 | Logistic regression | 77 |
|  |  |  | Jimenez-Serrano 2015 | Support Vector Machine (SVM) | 75 |
|  |  |  | Jimenez-Serrano 2015 | ANN | 66 |
| 19 | Krishnamurti et al [39] | 2022 | Krishnamurti 2022 | LASSO regression | 87 |
| 20 | Lilhore et al [41] | 2024 | Lilhore 2024 | NR | NR |
| 21 | Lilhore et al [40] | 2024 | Lilhore 2024 | CNN-BLSTM with a TL-based model | 100 |
|  |  |  | Lilhore 2024 | XGBoost | 100 |
|  |  |  | Lilhore 2024 | CatBoost | 99 |
|  |  |  | Lilhore 2024 | LightGBM | 98 |
|  |  |  | Lilhore 2024 | Random Forest | 85 |
|  |  |  | Lilhore 2024 | Decision Tree | 87 |
|  |  |  | Lilhore 2024 | AdaBoost | 82 |
| 22 | Liu et al [43] | 2024 | Liu 2024 | Logistic regression | 96 |
|  |  |  | Liu 2024 | Logistic regression | 97 |
|  |  |  | Liu 2024 | Logistic regression | 94 |
| 23 | Liu et al [42] | 2023 | Liu 2023 | Logistic Regression | 71.5 |
|  |  |  | Liu 2023 | Support Vector Machine (SVM) | 74.3 |
|  |  |  | Liu 2023 | Random Forest Classifier | 73.9 |
|  |  |  | Liu 2023 | Extreme Gradient Boosting | 74.4 |
|  |  |  | Liu 2023 | Light Gradient Boosting Machine | 72.7 |
|  |  |  | Liu 2023 | Multilayer Perceptron MLP | 70.6 |
| 24 | Lyall et al [44] | 2023 | Lyall 2023 | Ridge Regression | 74 |
|  |  |  | Lyall 2023 | Lasso Regression | 67 |
| 25 | Marshad et al [45] | 2024 | Marshad 2024 | AdaBoost | 76.13 |
|  |  |  | Marshad 2024 | XGBoost | 99.26 |
|  |  |  | Marshad 2024 | Gradient Boosting (GB) | 95.8 |
|  |  |  | Marshad 2024 | LightGBM | 96.13 |
|  |  |  | Marshad 2024 | CatBoost | 98.71 |
| 26 | Matsumura et al [46] | 2024 | Matsumura 2024 | Decision Tree | 84 |
| 27 | Matsuo et al [47] | 2022 | Matsuo 2022 | Logistic regression | 69.7 |
|  |  |  | Matsuo 2022 | Ridge regression | 70.2 |
|  |  |  | Matsuo 2022 | Elastic net | 70.1 |
|  |  |  | Matsuo 2022 | Kernel-based SVM | 64.2 |
|  |  |  | Matsuo 2022 | Random forest | 68.8 |
| 28 | Mazumder and Baruah [48] | 2021 | Mazumder and Baruah 2021 | Random forest | 94.7 |
| 29 | Moreira et al [49] | 2019 | Moreira 2019 | Weighted kNN | 95.7 |
| 30 | Mustafa [50] | 2023 | Mustafa 2023 | Naïve Bayes (NB) | 78 |
|  |  |  | Mustafa 2023 | Logistic regression | 78 |
|  |  |  | Mustafa 2023 | Support Vector Machine (SVM) | 77 |
|  |  |  | Mustafa 2023 | ANN | 68 |
| 31 | Myneni et al [51] | 2024 | Myneni 2024 | NR | NR |
| 32 | Nasim et al [52] | 2024 | Nasim 2024 | Stochastic Gradient Descent (SGD) | 74.5 |
|  |  |  | Nasim 2024 | Linear Regression (LiR) | 77 |
|  |  |  | Nasim 2024 | Random forest | 85.5 |
|  |  |  | Nasim 2024 | Bayesian Ridge (BR) | 77.5 |
|  |  |  | Nasim 2024 | Support Vector Machine (SVM) | 76.5 |
|  |  |  | Nasim 2024 | K-Neighbors Classifier (KNC) | 88.2 |
|  |  |  | Nasim 2024 | Multilayer Perceptron MLP | 91.2 |
|  |  |  | Nasim 2024 | Logistic regression | 77.5 |
|  |  |  | Nasim 2024 | Gaussian Naive Bayes (GNB) | 75.2 |
|  |  |  | Nasim 2024 | Gradient Boosting Classifier (GBC) | 91.7 |
|  |  |  | Nasim 2024 | Decision Tree | 87.7 |
| 33 | Natarajan et al [53] | 2017 | Natarajan 2017 | Naïve Bayes (NB) | 68.4 |
|  |  |  | Natarajan 2017 | Decision-trees (J48) | 90.2 |
|  |  |  | Natarajan 2017 | Support Vector Machine (SVM) | 70.9 |
|  |  |  | Natarajan 2017 | AdaBoost | 78.4 |
|  |  |  | Natarajan 2017 | Bagging | 56.5 |
|  |  |  | Natarajan 2017 | Logistic Regression | 75.6 |
|  |  |  | Natarajan 2017 | Gradient boosted tree (FGB | 95.2 |
| 34 | Osubor and Egwali [54] | 2018 | Osubor and Egwali 2018 | NR | NR |
| 35 | Park et al [55] | 2021 | Park 2021 | Logistic regression | 72.7 |
|  |  |  | Park 2021 | Random forest | 72.3 |
|  |  |  | Park 2021 | XGBoost | 73 |
|  |  |  | Park 2021 | Logistic regression | 77.9 |
|  |  |  | Park 2021 | Random forest | 77.8 |
|  |  |  | Park 2021 | XGBoost | 78.2 |
| 36 | Paul et al [56] | 2023 | Paul 2023 | Random forest | 71.18 |
|  |  |  | Paul 2023 | K-nearest neighbor (KNN) | 61.47 |
|  |  |  | Paul 2023 | Logistic regression | 69.56 |
|  |  |  | Paul 2023 | AdaBoost | 68.97 |
|  |  |  | Paul 2023 | Support Vector Machine (SVM) | 71.66 |
|  |  |  | Paul 2023 | Multilayer Perceptron MLP | 70.64 |
|  |  |  | Paul 2023 | TabNet | 77.79 |
|  |  |  | Paul 2023 | Recursive Partitioning (RPART) | 70.58 |
|  |  |  | Paul 2023 | C4.5 DT | 70.54 |
|  |  |  | Paul 2023 | Gradient Boosted Machines | 70.92 |
| 37 | Payne et al [57] | 2020 | Payne 2020 | Support Vector Machine (SVM) | 87 |
|  |  |  | Payne 2020 | Linear Model | 80 |
|  |  |  | Payne 2020 | Latent Dirichlet Allocation (LDA) | 72 |
| 38 | Prabhashwaree and Wagarachchi [58] | 2022 | Prabhashwaree and Wagarachchi 2022 | NR | NR |
| 39 | Prabhashwaree and Wagarachchi [59] | 2022 | Prabhashwaree and Wagarachchi 2022 | NR | NR |
| 40 | Qasrawi et al [60] | 2022 | Qasrawi 2022 | K-nearest neighbor (KNN) | 87.4 |
|  |  |  | Qasrawi 2022 | Decision Tree (DT) | 75.1 |
|  |  |  | Qasrawi 2022 | Support Vector Machine (SVM) | 86.7 |
|  |  |  | Qasrawi 2022 | Random Forest (RF) | 91.9 |
|  |  |  | Qasrawi 2022 | Neural Network (NN) | 90.8 |
|  |  |  | Qasrawi 2022 | Naïve Bayes (NB) | 92.1 |
|  |  |  | Qasrawi 2022 | Gradient Boosting (GB) | 93.5 |
| 41 | Raisa et al [61] | 2022 | Raisa 2022 | Random Forest (RF) | 98 |
|  |  |  | Raisa 2022 | XGBoost | 94 |
|  |  |  | Raisa 2022 | Support Vector Machine (SVM) | 88 |
|  |  |  | Raisa 2022 | Gradient Boosting (GB) | 91 |
|  |  |  | Raisa 2022 | Logistic regression | 91 |
| 42 | Reps et al [62] | 2022 | Reps 2022 | Gradient boosting machine | 73 |
|  |  |  | Reps 2022 | Gradient boosting machine | 70 |
| 43 | Shen et al [63] | 2023 | Shen 2023 | Naïve Bayes (NB) | 65.62 |
|  |  |  | Shen 2023 | K-nearest neighbor (KNN) | 80.02 |
|  |  |  | Shen 2023 | Support Vector Machine (SVM) | 75.03 |
|  |  |  | Shen 2023 | Random Forest (RF) | 65.05 |
|  |  |  | Shen 2023 | Logistic regression | 80.06 |
|  |  |  | Shen 2023 | Decision Tree (DT) | 82.02 |
|  |  |  | Shen 2023 | Deep Reinforcement Learning (RL)+Differential Evolution (DE)(Proposed model) | 90.66 |
|  |  |  | Shen 2023 | Proposed+random weights | 83.03 |
|  |  |  | Shen 2023 | Proposed+random weights+RL | 86.09 |
| 44 | Shin et al [64] | 2020 | Shin 2020 | Random Forest (RF) | 88.4 |
|  |  |  | Shin 2020 | Support Vector Machine (SVM) | 86.4 |
|  |  |  | Shin 2020 | Gradient boosting machine | 85.9 |
|  |  |  | Shin 2020 | AdaBoost | 85.7 |
|  |  |  | Shin 2020 | Naïve Bayes (NB) | 79.3 |
|  |  |  | Shin 2020 | Recursive partitioning (RPART) | 78.9 |
|  |  |  | Shin 2020 | K-nearest neighbor (KNN) | 77.6 |
|  |  |  | Shin 2020 | Logistic regression | 70.7 |
|  |  |  | Shin 2020 | Neural Network (NN) | 70.4 |
| 45 | Shivaprasad et al [65] | 2024 | Shivaprasad 2024 | NR | NR |
| 46 | Srivatsav and Nanthini [66] | 2024 | Srivatsav and Nanthini 2024 | NR | NR |
| 47 | Suganthi and Geetha [67] | 2024 | Suganthi and Geetha 2024 | NR | NR |
| 48 | Susič et al [68] | 2023 | Susič 2023 | NR | NR |
| 49 | Tang et al [69] | 2024 | Tang 2024 | NR | NR |
| 50 | Tortajada et al [70] | 2009 | Tortajada 2009 | Multilayer Perceptron MLP (Neural Net) | 82 |
|  |  |  | Tortajada 2009 | Multilayer Perceptron MLP (Neural Net) | 84 |
|  |  |  | Tortajada 2009 | Multilayer Perceptron MLP (Neural Net) | 80 |
|  |  |  | Tortajada 2009 | Multilayer Perceptron MLP (Neural Net) | 84 |
| 51 | Valavani et al [71] | 2022 | Valavani 2022 | NR | NR |
| 52 | Valdeolivar-Hernandez et al [72] | 2022 | Valdeolivar-Hernandez 2022 | NR | NR |
| 53 | Wagay [73] | 2023 | Wagay 2023 | Random Forest (RF) | 77.7 |
|  |  |  | Wagay 2023 | ExtraTree Classifier | 97.6 |
|  |  |  | Wagay 2023 | Light Gradient Boosting (LGB) | 97.51 |
|  |  |  | Wagay 2023 | CatBoost | 97.97 |
|  |  |  | Wagay 2023 | Extreme Gradient Boost (XGB) | 97.28 |
|  |  |  | Wagay 2023 | AdaBoost | 79.65 |
|  |  |  | Wagay 2023 | Hard Voting Ensemble Classifier(NN) | 97.76 |
|  |  |  | Wagay 2023 | Soft Voting Ensemble Classifier(NN) | 97.28 |
|  |  |  | Wagay 2023 | Weighted Majority Voting(NN) | 97.42 |
|  |  |  | Wagay 2023 | Stacking | 97.95 |
|  |  |  | Wagay 2023 | Nested Stacking | 98.95 |
| 54 | Wakefield and Frasch [74] | 2023 | Wakefield 2023 | Distributed Random Forest (DRF) | 91 |
|  |  |  | Wakefield 2023 | Logistic regression | 93 |
| 55 | Wang et al [78] | 2024 | Wang 2024 | NR | NR |
| 56 | Wang et al [76] | 2019 | Wang 2019 | Support Vector Machine (SVM) | 79 |
|  |  |  | Wang 2019 | L2-regularized Logistic Regression | 78 |
|  |  |  | Wang 2019 | Random Forest (RF) | 78 |
|  |  |  | Wang 2019 | Naïve Bayes (NB) | 78 |
|  |  |  | Wang 2019 | XGBoost | 77 |
|  |  |  | Wang 2019 | Decision Tree (DT) | 69 |
| 57 | Wang et al [75] | 2017-TRAJECTORY | Wang-TRAJECTORY 2017 | K-nearest neighbor (KNN) | 86.1 |
|  |  |  | Wang-TRAJECTORY 2017 | Decision Tree (DT) | 78.4 |
|  |  |  | Wang-TRAJECTORY 2017 | Random Forest (RF) | 91.7 |
|  |  |  | Wang-TRAJECTORY 2017 | Support Vector Machine (SVM) | 90.3 |
| 58 | Wang et al [77] | 2025-PLASMA | Wang-plasma 2025 | Logistic Regression | 93.75 |
| 59 | Xu et al [79] | 2023 | Xu 2023 | Support Vector Machine (SVM) | 81 |
| 60 | Xu and Sampson [80] | 2023 | Xu 2023 | NR | NR |
| 61 | Yu et al [81] | 2022 | Yu 2022 | random forest | 87.8 |
|  |  |  | Yu 2022 | random forest | 88.5 |
| 62 | Zhang et al [82] | 2020 | Zhang 2020 | Logistic regression | 88.6 |
|  |  |  | Zhang 2020 | Random Forest (RF) | 86 |
|  |  |  | Zhang 2020 | Decision Tree (DT) | 85.6 |
|  |  |  | Zhang 2020 | XGBoost | 86.4 |
|  |  |  | Zhang 2021 | Multilayer Perceptron MLP | 88.7 |
| 63 | Zhang et al [83] | 2024 | Zhang 2024 | Logistic regression | 91.8 |
| 64 | Zhang et al [84] | 2020 | Zhang 2020 | E-RF model | 75 |
|  |  |  | Zhang 2020 | E-SVM model | 81 |
|  |  |  | Zhang 2020 | F-RF model | 70 |
|  |  |  | Zhang 2020 | F-SVM model | 78 |
| 65 | Zhu et al [85] | 2021 | Zhu 2021 | NR | NR |
