# Supplementary material for: AI for Detecting and Predicting Postpartum Depression: Scoping Review
Source: J Med Internet Res. 2026 Jan 8;28:e77376. doi: 10.2196/77376 (PMC12782538; doi:10.2196/77376)
Supplement: Multimedia Appendix 9 [file jmir-v28-e77376-s009.docx]

## Multimedia Appendix 9. Generative AI transcripts.

**Prompt**: Please rephrase the following text for clarity while keeping technical accuracy:

Postpartum depression is affecting around 20% of mothers around the world. To find it early is very important for better results, but the current screening is not so scalable and dont have strong prediction. Using artificial inteligence like machine learning, deep learning and also natural language process can help to detect mothers who are at risk more acurately.

**Responses**: Postpartum depression affects up to 20% of mothers globally. Early detection is vital for better outcomes, yet screening lacks scalability and predictive power. Artificial intelligence—through machine, deep learning, and natural language processing—enhance the early identification of mothers at risk with greater accuracy.

**Prompt**: Rephrase the following paragraph for clarity while keeping technical accuracy:

We did not include studies if they were not empirical (for example reviews, editorials, or only abstracts), not writen in English, talking about perinatal mental health in general without specific focus on PPD, or if they used AI only for monitoring or treatment and not for prediction or detection. We made a systematic search in eight databases: MEDLINE, EMBASE, PsycINFO, CINAHL, Scopus, IEEE Xplore, ACM Digital Library and also Google Scholar, from the start untill Febuary 28, 2025.

**Response**: Studies were excluded if they were non-empirical (e.g., reviews, editorials, abstracts), not published in English, focused on general perinatal mental health without a specific emphasis on PPD, or used AI solely for monitoring or treatment rather than prediction or detection. We systematically searched eight databases—MEDLINE, EMBASE, PsycINFO, CINAHL, Scopus, IEEE Xplore, ACM Digital Library, and Google Scholar—from inception through February 28, 2025.

**Prompt**: Rephrase the following text for clarity while keeping technical accuracy:

The most common data used was sociodemographic (49 studies, 75.4%), then psychological data (44 studies, 67.7%) and obstetric data (36 studies, 55.4%). For data preprocessing, most of them only did simple scaling (78.5%) and some used missing data imputation (44.6%). Machine learning was used the most (80%), with Random Forest, SVM and logistic regression very common. For validation, internal methods like k-fold and hold-out were normal, but external validation was very rare. Ensemble boosting models showed better results in many metrics, showing good potential for accurate and scaleable prediction of postpartum depression.

**Response:** Sociodemographic data was most frequently used (49 studies, 75.4%), followed by psychological data (44 studies, 67.7%), obstetric data (36 studies, 55.4%). Data preprocessing mostly relied on basic scaling (78.5%) and some missing-data imputation (44.6%). Machine learning dominated (80%), especially Random Forest, SVMs, and logistic regression. Internal validation (k-fold, hold-out) was standard, while external validation was scarce. Ensemble-based boosting models consistently demonstrated superior performance across key metrics, highlighting their potential for accurate and scalable postpartum depression prediction.

**Prompt**: Polish the following paragraph for grammar and academic tone.

The main goal of this review is to show the AI methods that are used for PPD detection and prediction, and also to check the main research trends, methods used, and the gaps in evidence. This review try to answer the following questions:
• What kind of AI models are used to detect or predict PPD, and how they are different in way and complexity?
• What types of data (like structured, unstructured, physiological or digital) are used in these works?
• How the studies deal with model building like choosing features, validation and making it interpretable?
• What are the important limitations, problems, and also the future chance for using AI in PPD detection in real clinical and community environments?

**Response**: The primary aim of this review is to map the landscape of AI methodologies used in PPD detection and prediction and to identify key research trends, methodological features, and evidence gaps. Specifically, this review is guided by the following research sub-questions:

- What types of AI models have been used to detect or predict PPD, and how do they differ in approach and complexity?
- What data modalities (e.g., structured, unstructured, physiological, digital) have been employed in these studies?
- How have studies handled model development processes such as feature selection, validation, and interpretability?
- What are the key limitations, challenges, and future opportunities for applying AI to PPD detection in real-world clinical and community settings?

By addressing these questions, this review provides a structured, up-to-date, and integrative overview of AI in postpartum mental health—highlighting opportunities for innovation, responsible deployment, and policy translation in maternal care.

**Prompt**: Please proofread the following text:

As shown in Table 2, the average dataset size was 37,338.5 (SD = 160,309.6), but it range from only 16 up to 1,170,446. Around 28 studies (43.1%) had size between 500 and 5,000. Most studies (49, 75.4%) used closed-source data, and only 24.6% used open-source dataset. The data format was different like text, tables, audio, video and images. Majority (57, 87.7%) used only one type (unimodal), but 8 studies (12.3%) used many formats together (multimodal). Survey was the most used way to collect data (50 studies, 76.9%), then data from Electronic Health Records (EHRs) (25 studies, 38.5%). Most of the studies (45, 69.2%) were done in healthcare enviroments. For outcome measurement of PPD, 20 studies (30.8%) checked it in first 12 weeks after delivery (short term), 14 studies (21.5%) between 12 and 36 weeks (medium term), and 17 studies (26.2%) after 36 weeks (long term). The most used standard to label outcome was Edinburgh Postnatal Depression Scale (EPDS) (37, 56.9%).

**Response**: As depicted in Table 2, the average dataset size was 37,338.5 (SD = 160,309.6), with a range spanning from 16 to 1,170,446. The dataset size fell between 500 and 5,000 in 28 studies (43.1%). Most studies (49, 75.4%) utilized closed-source data, while the remaining 24.6% relied on open-source datasets. The studies included various data formats, including textual, tabular, audio, video, and images. The majority (57, 87.7%) used a single data format (unimodal), while the remaining 8 studies (12.3%) integrated multiple data formats (multimodal). The survey was the most common data collection approach (50 studies, 76.9%), followed by data sourced from Electronic Health Records (EHRs) (25 studies, 38.5%). Most studies (45, 69.2%) were conducted in healthcare settings. Regarding the timing of outcome measurement for PPD, 20 studies (30.8%) assessed outcomes within 12 weeks of delivery (short-term), 14 studies (21.5%) between 12 and 36 weeks (medium-term), and 17 studies (26.2%) after more than 36 weeks (long-term). The most common reference standard used for labeling the data (outcomes) was the Edinburgh Postnatal Depression Scale (EPDS) (37, 56.9%).

**Prompt**: Please improve the language of the text blow:

From Table 6, ensemble methods were the best models, with average accuracy 93.4%, F1-score 92.5% and AUC 89.4%. In the gradient boosting, CatBoost was highest with AUC 98.6% and also very strong accuracy and F1. LightGBM also show good result, with 92.6% accuracy, F1-score 87.8% and AUC 91.1%, showing it is scaleable and effective. XGBoost had also good performance, accuracy 89.1% and AUC 86.8%. Convolutional Neural Networks (CNNs) were also very strong, especially accuracy (92%) and F1-score (95.1%), but tested in less studies. Traditional tree models like Random Forest and recursive partitioning were more moderate. Random Forest reach 80.5% accuracy and 82.4% AUC, while other tree-based classifiers average 82.8% accuracy and 82.6% AUC. Recursive partitioning was lower, with 71.8% accuracy and 74.7% AUC. Overall, the average performance of all models was 81.7% ± 11.05 for accuracy, 80.51% ± 15.44 for F1-score and 81.0% ± 12.0 for AUC. These result show that deep learning and ensemble boosting models are very strong for PPD detection, usually better than normal machine learning models in most metrics. For more details about accuracy, F1 and AUC see Multimedia Appendix 9.

**Response**: Among the AI models evaluated in Table 6, ensemble methods emerged as the top performers, with an average accuracy of 93.4%, an F1-score of 92.5%, and an AUC of 89.4%. Among gradient boosting techniques, CatBoost achieved the highest AUC of 98.6%, alongside robust accuracy and F1-score metrics. LightGBM also demonstrated strong performance, recording 92.6% accuracy, an F1-score of 87.8%, and an AUC of 91.1%, highlighting its scalability and effectiveness. XGBoost delivered competitive results, with an accuracy of 89.1% and an AUC of 86.8%. Convolutional Neural Networks (CNNs) showed excellent performance as well—particularly in accuracy (92%) and F1-score (95.1%)—though they were evaluated in a smaller subset of studies.

Traditional tree-based models, including Random Forests and recursive partitioning, also showed moderate to strong performance. Random Forests achieved an average accuracy of 80.5% and an AUC of 82.4%, while broader tree-based classifiers averaged 82.8% accuracy and 82.6% AUC. Recursive partitioning, however, showed lower accuracy (71.8%) and AUC (74.7%) across the few studies assessed.

Across all models included, the mean performance was 81.7% ± 11.05 for accuracy, 80.51% ± 15.44 for F1-score, and 81.0% ± 12.0 for AUC. Collectively, these findings underscore the strong predictive capabilities of deep learning architectures and ensemble-based approaches—especially boosting models—in detecting postpartum depression, consistently outperforming conventional machine learning algorithms across most evaluation metrics. or detailed information on performance metrics (accuracy, F1-score, and AUC), see Multimedia Appendix 9.

**Prompt**: Please proofread the following text while keeping technical accuracy:

Looking at the results for accuracy, F1 and AUC, it is clear that ensemble models, specialy boosting methods like CatBoost, LightGBM and XGBoost, perform better than other AI methods for prediction of PPD. They have high accuracy and AUC, showing good generalization and robustness, because they can fix mistakes step by step and also learn complex non-linear patters, which is very useful in healthcare data that is noisy and not balance.

CatBoost was the best with AUC 98.6%, since it manage categorical variables well and have built-in control for overfitting, so it works very good for structured health data. LightGBM was also very strong, with 92.6% accuracy and very fast training using gradient-based sampling, so it is good for big data or real-time use. XGBoost also show competitive results (89.1% accuracy, 86.8% AUC) and is still very used because it give more transparency and feature importance.

**Response**: By considering the results of performance evaluation across accuracy, F1 score and AUC indicate that ensemble models, especially boosting techniques like CatBoost, LightGBM, and XGBoost, consistently outperformed other AI methods in predicting postpartum depression (PPD). Their high accuracy and AUC reflect strong generalization and robustness, owing to their ability to iteratively correct misclassifications and capture complex, non-linear patterns—particularly valuable in noisy, imbalanced healthcare datasets.

CatBoost led with an AUC of 98.6%, benefiting from its advanced handling of categorical variables and built-in overfitting control, making it highly suited for structured health data. LightGBM followed closely, offering high accuracy (92.6%) and efficiency due to its gradient-based sampling and fast training, making it ideal for large-scale or real-time applications. XGBoost also performed competitively (89.1% accuracy, 86.8% AUC), and remains popular for its transparency and feature importance tools

**Prompt**: Please proofread the following text while keeping technical accuracy:

To move forward in AI research for PPD, there are many important implications need to be improved. These include better methods, more data sharing, clinical use, and also ethical issues.

First, we need to add different kinds of data. Currenly, most studies only use sociodemographic and self-report surveys. But it is important to include things like language data (like sentiment analysis or speech patterns), biosignals (heart rate, activity), wearable devices, and biological markers (hormones, genetics, etc). This can make prediction more acurate and early.

Second, more open-access dataset should be shared. Only around 25% of studies use public data, which makes it hard to compare and reproduce. We need large and standard datasets, maybe with federated learning to keep privacy.

Third, more studies should use longitudinal designs. Most models use only retrospective or short-term postpartum data. But to understand symptoms over time and relapse, we need long-term data.

Fourth, multimodal fusion is important. PPD is complex, so models should combine structured data (like EHRs) with unstructured data (text, audio, sensors).

Fifth, preprocessing and feature engineering should be more standard. Now it is very different between studies, making hard to compare. Standard pipelines for feature extraction and imbalance adjustment (like SMOTE) are needed, with better reporting.

Sixth, explainability and ethics must be stronger. Models should be transparent and use methods like SHAP or LIME so clinicians can trust them. Also, bias should be reduced, false positives avoided, and cultural issues respected.

Seventh, evaluation metrics should be more consistent. Not only accuracy but also specificity, AUROC, F1 and precision should be reported. Reviews and meta-analyses are also needed.

Eighth, we should move from subjective screening tools like EPDS or PHQ to more objective measures, for example physiological or behavioral signals, so the validation is more reliable.

Ninth, ensemble models (CatBoost, LightGBM, XGBoost) show best results and should be tested in real-world use like clinics and mobile apps. CatBoost works well with categorical data and LightGBM is fast for big data. But because results change depending on dataset and preprocessing, benchmark data and standard pipelines are important.

Also, we must check performance in different groups of people, so there is no bias. More studies should see if boosting models stay strong when datasets become larger and more diverse.

Finally, global and interdisciplinary collaboration is needed. Research on PPD is not equal across the world. Working together between countries and also between computer science, psychiatry, public health and ethics can bring more fair and inovative solutions.

**Response**: To significantly advance the field of AI-driven postpartum depression (PPD) research, several key strategic priorities should be addressed. These priorities focus on improving methodological rigor, inclusivity of data, clinical applicability, and ethical implementation.

First, enhance the integration of multimodal and objective data sources: Currently, research predominantly relies on traditional sociodemographic and self-reported survey data. There is an urgent need to incorporate underutilized modalities such as linguistic data (e.g., LIWC, sentiment analysis, acoustic speech patterns), biosignals (e.g., heart rate variability, activity monitoring), wearable technology outputs, and biological biomarkers (e.g., hormonal, metabolic, genetic, or epigenetic markers). Leveraging these richer data types can significantly enhance the accuracy, personalization, and early detection capabilities of predictive models.

Second, expand and prioritize sharing of open-access datasets: Only about 25% of studies included in our review used publicly available datasets, highlighting a substantial barrier to reproducibility, benchmarking, and international collaboration. Developing standardized, large-scale, anonymized datasets should become a priority. Techniques such as federated learning could facilitate collaborative research across different institutions while maintaining data privacy and security.

Third, increase the adoption of longitudinal and prospective research designs: Most existing AI models for PPD prediction are based on retrospective or immediate postpartum data, limiting their ability to capture evolving symptom patterns over time. Incorporating longitudinal data collection into future studies is essential to better understand symptom progression, delayed onset, and relapse scenarios, thus enhancing the clinical relevance and predictive accuracy of AI models.

Fourth, advance multimodal fusion frameworks: Given the complexity of postpartum depression, future models must systematically integrate structured data (e.g., electronic health records) and unstructured inputs (e.g., text, audio, sensor signals). Developing robust multimodal fusion approaches that effectively combine diverse data sources will significantly enhance model interpretability, clinical effectiveness, and predictive power.

Fifth, standardize preprocessing and feature engineering pipelines: Variability and incomplete reporting in preprocessing methods currently limit model comparability and reproducibility. Adopting standardized protocols for data preprocessing—including feature extraction, transformation techniques, and class imbalance adjustments (e.g., SMOTE, cost-sensitive learning)—is necessary. Transparent reporting of these processes should be enforced to enhance scientific rigor and validation.

Sixth, emphasize model explainability and ethical AI practices: Transparent and interpretable AI models are crucial for clinical adoption. Few studies currently apply advanced explainability methods such as SHAP, LIME, or counterfactual analyses. Integrating these interpretability techniques into AI pipelines will facilitate clinician trust and understanding. Moreover, ethical considerations—such as minimizing algorithmic bias (such as balanced data sets, resampling to ccorrect imbalances in training data) are considered in few studies, but preventing potential harms from false positives, safeguarding patient autonomy, and ensuring cultural sensitivity—should be systematically addressed in all AI model developments.

Seventh, standardize evaluation and reporting metrics: While accuracy is often prioritized, metrics such as specificity, AUROC, F1-score, and precision must be consistently reported to enable comprehensive evaluation and meaningful comparisons across studies. Furthermore, systematic reviews and meta-analyses are required to identify existing methodological inconsistencies, biases, and underrepresented findings to refine future AI-based research approaches.

Eighth, shift from subjective screening tools to objective validation measures. Current studies heavily rely on subjective instruments (e.g., EPDS, PHQ, ICD codes), which, despite validation, vary significantly across studies. Future research should validate AI models using objective clinical measures such as physiological indicators and behavioral markers, thus improving reliability and facilitating clinical implementation.

Ninth, the superior performance of ensemble models—particularly boosting techniques such as CatBoost, LightGBM, and XGBoost—suggests they are promising candidates for clinical implementation in PPD screening. Their ability to consistently achieve high accuracy, F1-score, and AUC underscores their robustness in handling structured health data, including demographic and clinical features. Given the strong results of CatBoost in handling categorical variables and LightGBM’s efficiency in large-scale settings, future research should prioritize evaluating these models in real-world clinical workflows and mobile health platforms, where scalability and interpretability are critical. Additionally, since performance varied across studies due to differences in data characteristics and preprocessing strategies, future work should aim to establish benchmark datasets and standardized pipelines for fair comparison. Efforts should also be made to assess model performance across different population subgroups, ensuring that these algorithms do not inadvertently introduce or amplify bias. Finally, comparative studies should continue to assess whether boosting models maintain their advantage as datasets grow and diversify, particularly in longitudinal or multi-site contexts.

Last, foster global and interdisciplinary collaboration: PPD research remains unevenly distributed globally. Encouraging cross-regional and interdisciplinary collaboration—particularly with underrepresented regions and diverse professional backgrounds such as computer science, psychiatry, public health, and ethics—will foster equitable research practices and drive innovation in maternal mental healthcare globally.

**Prompt**: Please proofread the following text while keeping technical accuracy:

Even though this scoping review is comprehensive, there are some limitations that we have to acknowledge. First, we only include studies published in English. This may exclude some important research in other languages, specially from countries where maternal mental health is also very important.

Second, we focus on peer-reviewed and indexed papers from eight big databases, and from Google Scholar we only take the first 100 by relevance. Because of this, grey literature like government reports, dissertations not in ProQuest, or some conference works not indexed may be missed.

Third, our review only look at studies using AI for prediction or detection of postpartum depression. Studies that use AI for monitoring, treatment or resource allocation in maternal health are not included, so the scope is more narrow.

Last, we did not do a quantitative meta-analysis or risk of bias analysis, because this is usually not in scoping reviews. So even if we show the methods and the gaps, we did not measure effect size, heterogenity or quality of each study in a systematic way.

**Response**: Despite the comprehensive nature of this scoping review, several limitations should be acknowledged, first, we limited our inclusion to studies published in English. This language restriction may have resulted in the exclusion of relevant research published in other languages, particularly from non-English-speaking countries where maternal mental health may be a pressing issue.

Second, we prioritized peer-reviewed and indexed literature from eight major databases and limited Google Scholar results to the first 100 entries ranked by relevance. Consequently, grey literature, including government reports, dissertations beyond ProQuest, and non-indexed conference proceedings, may have been underrepresented.

Third, our review focused specifically on studies using AI techniques for detection or prediction of postpartum depression. As a result, studies that applied AI for monitoring, treatment delivery, or resource allocation in maternal mental health were excluded, which narrows the scope of applicability.

Lastly, we did not conduct a quantitative meta-analysis or risk of bias assessment, as these are typically outside the scope of scoping reviews. Consequently, while we mapped methodological patterns and gaps, we did not evaluate effect sizes, statistical heterogeneity, or study-level quality in a standardized manner.

**Prompt**: Please proofread the following text while keeping technical accuracy:

This scoping review gives a map of how AI is used in postpartum depression (PPD) research, looking at 87 studies from 2009 to 2025. The review show that most focus is on early prediction (around 80%) more than detection. Machine learning (ML) was used in most of the studies (87.7%), with Random Forest (44.6%), logistic regression (35.4%) and XGBoost (23.1%) the most common. These models were prefered because they fit well with structured clinical data and are more easy to interpret. Deep learning (DL) methods like CNN and RNN were used less (16.9%), maybe because of data limits and problem with interpretability. Natural language processing and reinforcement learning were very rare, because there is not much unstructured or sequence data available.

**Response**: This scoping review comprehensively maps the application of artificial intelligence (AI) in postpartum depression (PPD) research, analyzing 87 studies published between 2009 and 2025. The review identifies a predominant emphasis on early prediction (∼80%) over detection, with machine learning (ML) methods—particularly Random Forest (44.6%), logistic regression (35.4%), and XGBoost (23.1%)—used in 87.7% of studies. These models were favored for their compatibility with structured clinical data and interpretability. Deep learning (DL) approaches, including CNNs and RNNs, were underutilized (16.9%), reflecting data limitations and interpretability concerns. Natural language processing and reinforcement learning were rarely applied, mirroring limited access to unstructured or sequential data sources.
